# Stakeholder participation, indicators, assessment, and decision-making: applying adaptive management at the watershed scale

**DOI:** 10.1007/s10661-021-09741-4

**Published:** 2022-02-08

**Authors:** Adriana A. Zuniga-Teran, Larry A. Fisher, Thomas Meixner, François-Michel Le Tourneau, Frank Postillion

**Affiliations:** 1 School of Geography, Development & Environment, University of Arizona, Tucson, USA; 2 Udall Center for Studies in Public Policy, University of Arizona, Tucson, USA; 3 School of Natural Resources and the Environment, University of Arizona, Tucson, USA; 4 Department of Hydrology and Atmospheric Sciences, University of Arizona, Tucson, USA; 5 iGLOBES International Research Laboratory Centre National de la Recherche Scientifique, Paris, France and University of Arizona, Tucson, USA; 6 Postillion ERC Inc., Tucson, USA

**Keywords:** Adaptive management, Watershed health assessment, Environmental monitoring, Ecological and social indicators, Stakeholder participation

## Abstract

Climate change, population growth, and declining federal budgets are threatening the health of ecosystems, and the services they provide. Under these changing conditions, managing landscapes and resources assumes new and unprecedented challenges. Adaptive management has been identified as a natural resource management approach that allows practitioners to incorporate change and uncertainty into decision-making through an iterative process that involves long-term monitoring and continued review and adjustment of management actions. However, the success of these efforts in watershed health relies on the collective and sustained monitoring of indicators, which is seldom studied. The purpose of this analysis is to examine (1) the practical challenge of choosing a list of indicators for long-term monitoring, (2) the negotiation process among stakeholders around the selection and interpretation of indicators, and (3) the communication tools that can be used to convey the assessment’s results and findings. To do this, we analyze our ongoing work in the Cienega Watershed in southern Arizona. Our analysis shows that the selective use of indicators, regular assessment and review, and establishment of partnerships among stakeholders are all important elements in establishing effective adaptive management efforts. The selection of indicators and data sources is a moving target that requires regular consensus and review among stakeholders. The assessment itself is also a powerful engagement tool with the public at large, providing legitimacy and support to land management decision-making. Here, we outline some lessons learned that can be transferred to other cases and identify potential barriers for engagement, decision-making, and project success.

**Supplementary Information:**

The online version contains supplementary material available at 10.1007/s10661-021-09741-4.

## Introduction

Watershed health is critical for the provision of a wide range of ecosystem services. However, there is no one-size-fits-all approach to watershed management (Porzecanski et al., 2012). Optimum watershed function depends on the coupling of social and ecological systems and the outcomes derived from these interactions (Ostrom, 2009). In the USA, as well as in most of the western world, watersheds are increasingly threatened by urban expansion, population growth, land use change, fire, invasive species, and climate change. Environmental change can be unpredictable and unrecognizable as a consequence of the stochasticity in ecological processes (Williams, 2011). Managing natural resources under these conditions of change and uncertainty therefore becomes a major challenge. Management decisions at the watershed scale are critical in addressing the challenges and tensions between supply and demand of resources (Sadeghi et al., 2020).

Changes in hydrological response to land use, land cover change, and climate change are context specific, as the characteristics of ecohydrological systems and their responses to human and environmental changes are unique (Gorelick et al., 2020). In addition, global environmental change adds more uncertainty to decision-making processes. As cities grow and climate change threatens the availability of water resources and the provision of ecosystem services, it becomes important to manage resources under unprecedented uncertainty.

Given these dynamic interactions between social and ecological systems, adaptive management (AM) has been identified as a useful approach to natural resource management (Brownson & Fowler, 2020; Porzecanski et al., 2012; Sadeghi et al., 2020; Varady et al., 2016; Williams, 2011). AM is needed to assess “the impacts of hydrologic change on water availability” (Gorelick et al., 2020: 24), as it allows land and water managers to *learn by doing*, testing hypotheses, and assessing outcomes in an iterative process (Varady et al., 2016). AM uses results from long-term monitoring to inform land management practices, by incorporating uncertainty into decision-making processes (Porzecanski et al., 2012; Williams, 2011). This management approach promotes stakeholder collaboration, draws on data from multiple sources, and uses a variety of models to increase effectiveness (McLain & Lee, 1996). AM has been adopted by many natural resource management agencies, and via emerging collaborative governance systems has been used to manage complex social and ecological systems (Brownson & Fowler, 2020).

AM started as a management approach to address structural uncertainty, or the “structure of the resource system and the processes (such as survivorship and reproduction) that influence its dynamics” (Williams & Brown, 2018: 996). In this initial conceptualization of AM, decision-making is considered a periodic sequence, where learning is used to adjust management strategies in an iterative, technically-focused learning process. For example, Gorelick et al. (2020) describe AM as a dynamically-updating risk mitigation process used to determine water supply reliability for water utilities. Here, the adaptive aspect of the management process is used to address uncertainties in the risk to provide water (demand growth, decreased precipitation) in a financially responsible way, in what is known as a single-loop learning process. However, the institutional uncertainties that arise from the impacts of management decisions were not considered in this technical AM framework. Gorelick et al. (2020) involved stakeholders (utility/water managers) to inform water supply modeling, but did not complete a second learning loop — returning lessons learned to decision-makers to improve their own decision-making. Over time, the AM framework evolved to address this gap and became a double-loop learning process that considers two types of uncertainties — uncertainty of watershed resource dynamics (structural) and the impacts of management actions (institutional) (Williams & Brown, 2018). More recently, a triple-loop learning cycle has been proposed for AM to further consider governance structures that address power dynamics and foster a type of social learning that accommodates changes in values and norms (Johannessen et al., 2019; Pahl-Wostl, 2009; Williams & Brown, 2018).

Despite its obvious benefits and its numerous proponents, AM has rarely been successfully implemented (Porzecanski et al., 2012). Scholars agree that additional research is needed to identify best practices in AM, particularly around the information used for long-term monitoring in projects with large numbers of stakeholders (Brownson & Fowler, 2020). How can stakeholders collectively create a monitoring system that bridges the information gaps between them, is scientifically sound, offers continuity over the long term, and speaks to the public? This paper seeks to address this gap through the analysis of our ongoing work with the State of the Cienega Watershed assessment.

Our primary goal in this analysis is to extract lessons that can be used as best practices for other similar efforts. While we do not claim that this study uses an experimental research approach, the paper does offer a broad and exploratory approach to addressing a critical question of *practice* — how to assess ecosystem health within a watershed, and particularly, how to do this within the framework of adaptive management and stakeholder participation. In doing so, we contribute to the literature by providing insights on the practical challenge of selecting indicators that reconcile the challenges of data availability with desirability and relevance, taking into account stakeholder interests and priorities. We also provide important lessons about the negotiation process among stakeholders around the indicators to be used in the assessment and their interpretation. Finally, we describe the communication tools that have been used to convey the state of the health of the watershed to stakeholders and to the wider public.

## Background

### Adaptive management at the watershed scale

Watersheds have been identified as the best ecological unit for the management and governance of natural resources (Cohen, 2011). The expansion of urban infrastructure disrupts watershed hydrology — the magnitude and timing of runoff affecting streamflow and storage levels (Gorelick et al., 2020). Land management actions implemented by individual property owners (e.g., clearcutting, conversion of the natural landscape into cropland, real estate development, and other changes in land use), may have profound and cumulative implications on ecological processes at the landscape scale (O’Neill et al., 1997). Changes in streamflow, through water withdrawals and projected extreme events (flooding and drought), interact with land use and land cover change, affecting water availability. In addition, water management policies (e.g., water conservation programs, usage restrictions, water transfers, infrastructure expansion) can have significant impact on hydrological systems (Gorelick et al., 2020; Scott et al., 2012). Therefore, to reinforce the cyclic nature of decision-making, institutional and technical learning must be incorporated into the AM framework (Williams & Brown, 2018).

AM at the watershed scale has the potential to enhance natural resource management; however, doing so presents important challenges. Even in the USA, with its established financial capacity, institutions, and legal frameworks, long-term monitoring, so essential to AM effectiveness, is difficult to implement and sustain over time. In their literature review of AM initiatives, Brownson and Fowler (2020) find that sustained funding still represents a limitation to effective monitoring. This is especially true in terms of the technical capacity to demonstrate a return on investment from management programs that can either satisfy old investors or attract new ones. In addition, funding is critical to establishing long-term baseline datasets that are essential for long-term monitoring. In some cases, for example, on public lands, watershed management programs have access to secure funding from regulatory drivers at the federal level (e.g., Clear Water Act, Endangered Species Act) (Brownson & Fowler, 2020). But in other cases, local governments or community-based organizations do not have the financial or institutional capacity to monitor indicators themselves, or hire consultants to do it for them, let alone coordinate monitoring efforts between different organizations. Brownson and Fowler also found that capacity building and enhanced evaluation methods are ongoing needs in AM projects. Stakeholder participation is therefore seen as a critical factor for successful long-term monitoring efforts. Although having a large number of people involved in the assessment may increase the potential for conflicts over goals and objectives, in general, a larger group of stakeholders is desirable because it may enable greater access to resources, technical assistance, and institutional capacity. In short, the more stakeholder interests involved in AM, the more likely that monitoring activities will be coordinated, and the more likely the effort will be able to find adequate resources and have the necessary capacity to implement AM over time (Brownson & Fowler, 2020).

Universities and scientists play an important role in AM, as they provide important expertise and critical information resources. Scientists can be considered learning champions, or even change agents, because they have the technical expertise and the ability to drive social learning processes by connecting information with its implications to decision-making (Johannessen et al., 2019). Interdisciplinary assessments that consider biophysical and social monitoring can be useful in engaging additional groups and tapping into different funding sources (Brownson & Fowler, 2020). Scientists have a unique opportunity to communicate science to the general public.

### Long-term monitoring of indicators

A major aspect of ecosystem assessment is the continuous monitoring of suitable indicators that can provide early warning signals for complex environmental dynamics (Dale & Beyeler, 2001; Feld et al., 2010). Through monitoring, managers have access to data about the state of natural resource systems that can inform decision-making (Williams & Brown, 2018). Scholars recommend the use of indicators that capture environmental processes at the landscape scale, such as climate information, changes in land cover, and the configuration of habitat patches that can illustrate landscape stability, biodiversity, and ecosystem integrity (O’Neill et al., 1997). Watershed health assessments use a range of ecological indicators to inform decision-making and policy on land and water resource management (He et al., 2000). It is helpful to identify indicators that simplify and quantify complex biophysical interactions at different scales, so that they are sensitive to change and are readily communicated to stakeholders and the larger public (Feld et al., 2010). Assessment efforts use indicators that reflect biophysical and hydrological processes and changes in the temporal and spatial distribution of watershed conditions (He et al., 2000); AM approaches must also include socio-economic indicators that measure the dynamic interactions between environmental and social systems (Morton & Padgitt, 2005). For example, Kim et al. (2021) explored the benefits and pitfalls of management strategies in the forests of South Korea, finding important tradeoffs between recreational uses and carbon sequestration, and between freshwater supply and wood production. Similarly, El Mahrad et al. (2020) studied the socio-environmental interactions derived from the management of coastal lagoons in Northern Africa; they found that science-based knowledge is critical to informed decision-making that can result in sustainable outcomes. Nevertheless, selecting an appropriate list of indicators represents a major challenge for AM efforts, as the list must be short enough to be manageable, but long enough to represent the complexity of these systems (Dale & Beyeler, 2001). To be sustainable over the long term, AM efforts should use indicators that are simple, easy to monitor, inexpensive, and preferably those that land managers regularly monitor as part of their normal routines. At the same time, they should be sensitive to stresses and predictable of major disturbances (Dale & Beyeler, 2001).

Monitoring can be expensive and time-consuming; therefore, dedicated funding is needed, as well as stakeholder commitment for continuous monitoring efforts. There are important monitoring challenges to consider in AM, including the changing focus of monitoring efforts. As learning processes occur, monitoring protocols must be adapted to changing priorities. In addition, AM needs to account for tradeoffs that exist between monitoring costs and precision, the frequency of monitoring, and changes in stakeholders’ values, priorities, and attitudes (Williams & Brown, 2018).

Assessing the health of a watershed is a complicated task. Which indicators are most helpful in determining watershed health? Who decides the criteria for selecting the indicators? How can data be collected in a watershed with multiple jurisdictions and landowners? Who funds this comprehensive effort over time? How can this information be used to inform land management decisions? What are effective communication tools to convey the watershed health assessment to stakeholders and the general public? In this paper, we analyze the State of the Cienega Watershed to seek answers to these questions.

## Methodology

### The State of the Cienega Watershed

Convened by the Cienega Watershed Partnership (CWP), a non-profit organization (501c3), the State of the Cienega Watershed engages a diversity of actors who share the common goal of protecting the health of the Cienega Watershed. CWP is a multi-stakeholder effort that has been recognized nationally for its collaborative adaptive management approach, receiving the Secretary of Interior’s Partnership award in 2013 for “promoting understanding and stewardship of the natural and cultural resources of the more than 45,000-acre (18,000 ha) Las Cienegas National Conservation Area.”^1^

After almost 20 years of commitment to an adaptive management framework, and, more recently, five years of continuous monitoring of a selected set of indicators, the State of the Cienega Watershed initiative is a unique example of interdisciplinary assessment and collaborative resource management. The efforts have produced a coalition of decision-makers and a long-term collaboration whose members — academics, federal, state and local government agencies, non-profit organizations, local ranchers and residents — actively share information and resources. This work also reflects the three components of community capacity identified by Brinkman et al. (2012) — shared vision, empowerment, and collective action.

### Location and threats

The Cienega Watershed, a hydrologic unit code (HUC) 10 watershed^2^ within the Santa Cruz River watershed, is located in southeastern Arizona. It contains one of the last remaining perennial creeks of the region — Cienega Creek. The watershed has historically included many *cienegas* (Spanish for wetlands), hence its name. Wetlands are critical ecosystems that are vulnerable under changes in hydrological regimes, and particularly as a consequence of climate change (Cassatt & Wilcox, 2020).

**Fig. 1:**
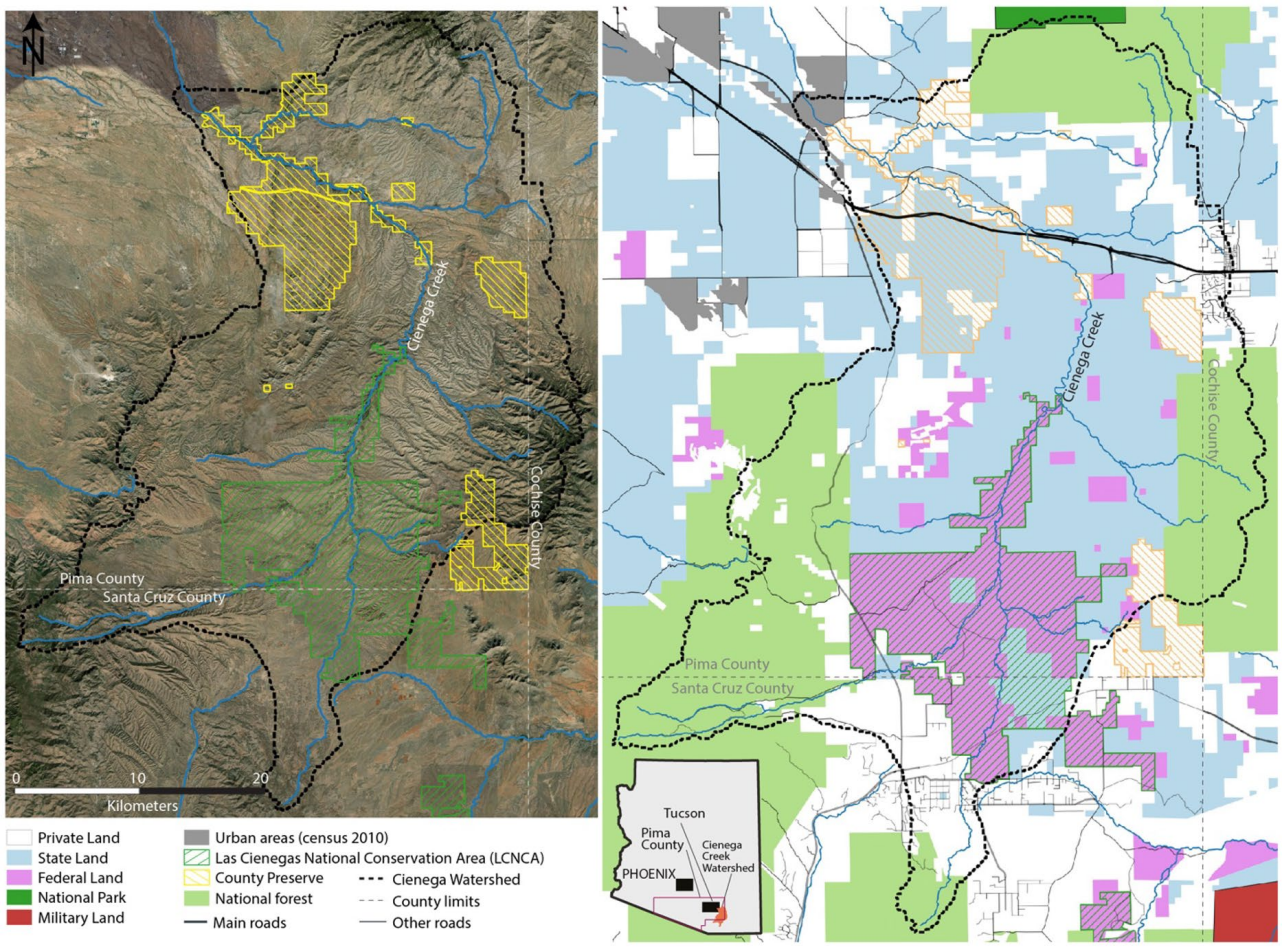
Land ownership and jurisdictions for the Cienega Watershed (satellite imagery map by F-M Le Tourneau)

The watershed, with a total area of 300,000 acres (120,000 ha), is located in an extended grassland valley at an average elevation of 4000 feet (1220 m). Land in the valley is of mixed jurisdiction. Although the centerpiece is the Bureau of Land Management (BLM)’s Las Cienegas National Conservation area (LCNCA), the watershed encompasses considerable State Trust Lands, as well as lands managed by the U.S. Forest Service, Pima County, the Department of Defense, and numerous private landowners, some with conservation easements (Fig. 1). The LCNCA’s grasslands and woodlands help to maintain connectivity among several of the region’s sky island mountain ranges and play a pivotal role in protecting regional wildlife linkages (Beier et al., 2011).

The LCNCA (47,279 acres, 19,133 ha) and the larger Sonoita Valley Acquisition Planning District (SVAPD) (95,609 acres, 38,692 ha) were designated by Congress and signed into law in December 2000 “in order to preserve, protect, and enhance the unique and nationally important aquatic, wildlife, and vegetative, archaeological, paleontological, scientific, cave, cultural, historical, recreational, educational, scenic, rangeland, and riparian resources and values of the public lands within the NCA, while allowing livestock grazing and recreation to take place in appropriate areas” (BLM, 2000). The LCNCA contains five of the rarest plant communities in the arid Southwest: cienega wetlands, cottonwood-willow riparian forests, sacaton grasslands, mesquite bosques, and semidesert grasslands, and its landscapes support several threatened and endangered species (Caves et al., 2013). The Cienega Watershed and the Sonoita Valley are also renowned for their archaeological sites and for more recent western cultural heritage, including the Empire Ranch (Majewski et al., 2004). Management of the LCNCA is based on multiple use objectives, as identified in the Establishment Act and includes an active cattle grazing program and a variety of recreational activities (Stevens, 2001).

In addition to the LCNCA, the Cienega Creek Natural Preserve encompasses approximately 4000 acres (1618 ha) along 12 mi (19 km) of Cienega Creek in the northern portion of the watershed. The Preserve is owned by the Pima County Regional Flood Control District and jointly managed with the County’s Natural Resources, Parks, and Recreation Department (NRPRD). The preserve was set aside to protect the riparian channel and vegetation, allowing for over-bank recharge of flood waters, reducing the need for flood control improvements downstream (Pima County, n.d.). Protection also increases water levels in the underlying aquifer for the Tucson basin. In addition to its hydrological importance, the Preserve provides important habitat for wildlife, including rare and endangered aquatic species, and maintains a critical corridor for wildlife migration between the Santa Rita, Whetstone, and Rincon mountain ranges. Finally, like the LCNCA, the Preserve provides for recreational and educational activities, which allow the public to enjoy and learn about this unique area.

Nevertheless, these important conservation areas, and their ecological and socio-cultural values are increasingly threatened by development, invasive species, wildfire, and climate change (Bodner & Robles, 2017; Goodrich et al., 2020; Stromberg et al., 2009). Dewatering of Cienega Creek, through increased settlement and economic activity, has placed increasing pressure on several threatened and endangered species (Stromberg et al., 2009), including the Chiricahua Leopard Frog (*Lithobates chiricahuensis),* Gila topminnow (*Poeciliopsis occidentalis*), Gila chub (*Gila intermedia*), longfin dace (*Agosia chrysogaster*), Southwest willow flycatcher (*Empidonax trailii extimus*), lesser long-nosed bat (*Leptonycteris yerbabuenae*), Huachuca water umbel (*Lilaeopsis schaffneriana* var. *recurve*). Invasive species, including bullfrogs (*Lithobates catesbeianus*), Lehmann lovegrass (*Eragrostis lehmanniana*), and mesquite (*Prosopis* spp.*,* a native shrub species that has become invasive over the past decades), pose increasing risk to the riparian ecosystem and to grassland function and health (Bodner & Robles, 2017). Wildfires have increased in frequency and intensity over the past decade (Goodrich et al., 2020). Another major concern for the watershed health is the threat of large-scale mining in the area.^3^ If approved, plans to permit this large copper mine further threaten to significantly alter local hydrological systems and ecosystem function. Concerns for landscape connectivity, loss of cultural resources, and declining federal budgets compound the series of issues facing the watershed.

### Community engagement and the Cienega Watershed Partnership

The area boasts a long history of active public engagement. Local residents advocated for the land exchange that eventually brought private lands into public ownership as the LCNCA. In 1995, the BLM sponsored a broad-based collaborative planning process to develop the unit’s resource management plan (RMP) (Caves et al., 2013). During the planning process, the collaborative group opted to incorporate an AM approach, underscoring the need for sustained monitoring and flexible, collaborative decision-making. The jointly developed RMP incorporates this collaborative adaptive management (CAM) approach into its guidelines, referred to in the RMP as Biological Planning, with the intention of providing agency decision-makers with both credible information on resource conditions and trends, while continuing the public engagement and the scrutiny that has been critical to maintaining agency accountability.

CWP grew out of these activist public engagement efforts and has since become a consistent advocate for the CAM approach. For almost 20 years, CWP has helped convene the twice annual Biological Planning sessions, where agency managers, researchers, ranchers, non-profit groups, and local citizens gather on the LCNCA to review monitoring data and take stock of grassland and riparian conditions. CWP hosts an annual Science on the Sonoita Plain Symposium, designed to promote exchange among scientists, land managers, local landowners, interested citizens, and students about the results of scientific investigations of the unique and diverse resources in the upper watersheds of Cienega Creek, Sonoita Creek, and the Babocomari River.

Since 2008, CWP has also sponsored an annual meeting, the State of the Cienega Watershed. The State of the Cienega Watershed provides an additional opportunity to conduct a regular assessment of conditions and trends in the Cienega Watershed, drawing on a wide array of existing data to provide a mechanism for long-term monitoring, evaluation, and adaptation of CWP program priorities and actions. The annual State of the Cienega Watershed meeting is yet another important milestone that reinforces the collaboration between CWP, the BLM, Pima County, and many partner organizations and individuals concerned about the health of the Cienega Watershed. We provide a list of stakeholders in Table 1 below.

**Table 1:** List of stakeholders and their role in the State of Cienega Watershed assessment

**Stakeholder affiliation**	**Department/division**	**Role in the State of the Cienega Watershed**
Cienega Watershed Partnership		NGO that brings together stakeholders and the general public to foster stewardship of the health of the watershed
Pima County	Regional Flood Control District	In charge of slowing down flow of the creek to avoid flooding downstream
	Parks and Recreation	Manages the recreational aspect of the county preserve
	Cultural Resources & Historic Preservation Division	Manages archaeological sites and monitors their state
	Office of Sustainability & Conservation	Manages landscape health
Pima Association of Governments		Monitors water resources
Bureau of Land Management		Manages the land to comply with federal regulations
US Forest Service		Manages the national forests land
Arizona Game and Fish Department		Manages wildlife for hunting and fishing
Arizona State Land Department		Owns land on the watershed, usually rented to ranchers
US Geological Survey		Monitors the landscape using remote sensing data
The University of Arizona		Provides expertise on several indicators in particular and adaptive management in general
Vail Preservation Society		Manages cultural resources
Desert Botanical Gardens		NGO that monitors wildlife movements and ecosystem health
Sky Island Alliance		NGO that monitors wildlife movements and ecosystem health
The Nature Conservancy		NGO that works toward ecosystem health. They partner with BLM
Empire Ranch Foundation		Stewards the Empire Ranch
iGlobes — Centre National de la Recherche Scientifique		French research center affiliated with the University of Arizona interested in human-landscape interactions

The idea of an annual “state of the watershed” review, using a select group of indicators, was first introduced during the 2015 meeting (February, 2015). CWP invited representatives from several other watershed management and environmental conservation programs to share their experiences evaluating ecosystem health.^4^ Many of these initiatives employed routine monitoring of a core set of indicators, used visually appealing ways of presenting their results, and sponsored regular convening events with key stakeholders to evaluate trends and recommend adaptations to management decision-making.

The group also relied on recent assessments conducted in the region — a 2010 LCNCA threats analysis, the LCNCA Data and Gaps Analysis workshop (November 2013), the scenario planning resource prioritization workshop (June, 2013), BLM’s Madrean Archipelago Rapid Ecoregional Assessment (BLM, 2015), and the ongoing Pima County Watershed Assessment.

## Results

In this study, we seek to provide insights into the practice aspect of adaptive management by analyzing our work in southeastern Arizona, where a group of stakeholders are working together to monitor the health of a watershed over time. We focus on (1) the challenge of establishing an indicator system for the Cienega Watershed, (2) stakeholder engagement in the negotiation, selection, and interpretation of indicators, and (3) efforts to communicate results of the assessment. We received 40 responses (47.6%).^5^ It is important to emphasize that the survey should not be interpreted as an attempt to achieve accurate representation of stakeholder numbers; it was designed as a means to elicit additional input from a wider array of stakeholder interests, to augment the in-person discussions occurring during the State of the Watershed workshop and the meetings of the technical teams. The ranking of the selection criteria is presented in Table 2 below.

**Table 2:** Ranking of top nine selection criteria for indicators (*N* = 40)

**Order**	**Selection criteria**
1	Measures impacts of change and the thresholds that threaten ecosystem integrity
2	Produces useful information for management in the short and long term
3	Repeatable, comparable, consistent
4	Simple and cost-effective to collect
5	Can expose threats and vulnerability
6	Quantifiable
7	Transferable and applicable to management across jurisdictions
8	Data already exists or is readily available
9	Data speak to the public

Based on the survey responses, and following considerable discussion and adjustment within the technical teams and in the plenary session, the participants agreed on the following 20 indicators:

### Negotiation process between stakeholders around indicators and their interpretation

**Fig. 2:**
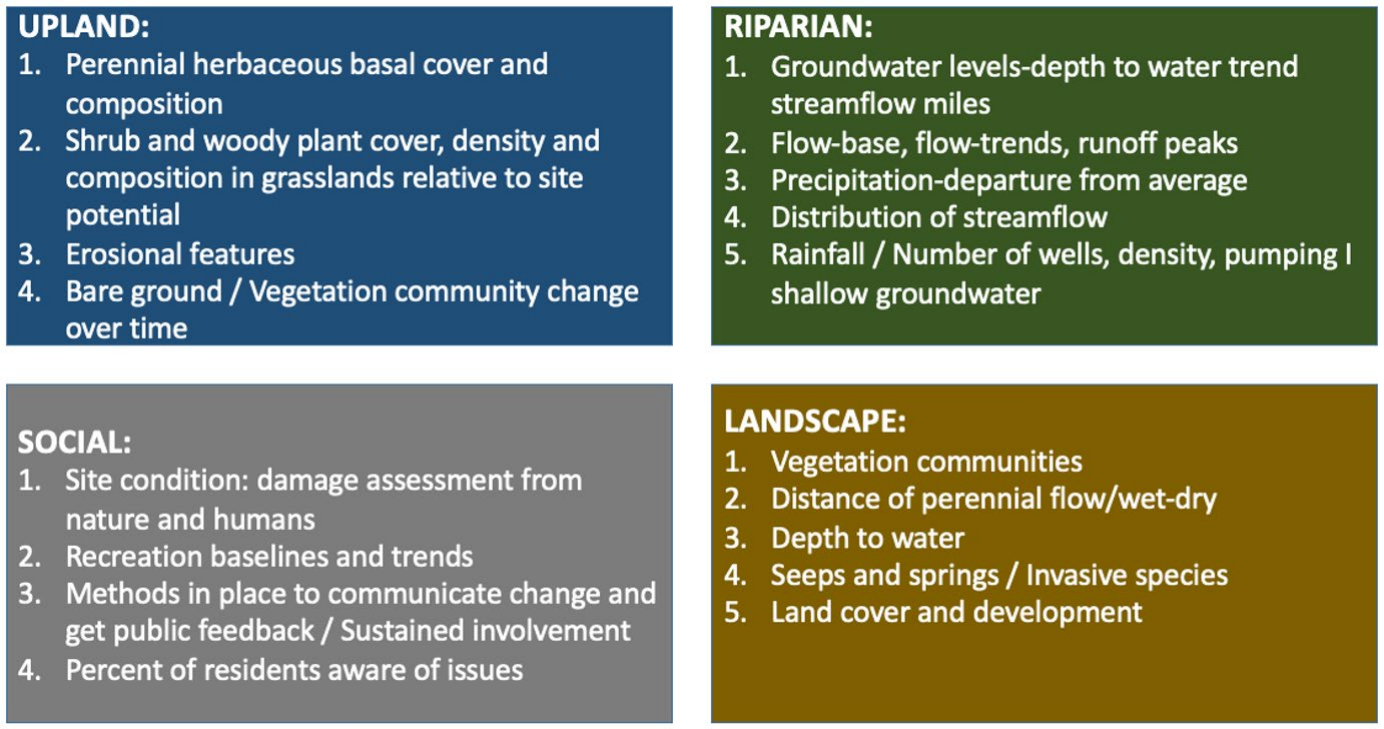
List of indicators retained by category

Through extensive contact with key CWP partners, we then set about gathering and synthesizing available data (and, as necessary, identifying proxy data), and determining the best way to present and communicate information for each of these indicators. The authors held periodic meetings with CWP’s SOW project team to further refine data analysis and enhance the presentation of results (Fig. 2).

In March 2017, we presented the first report on the State of the Cienega Watershed to 45 CWP partners, including representatives from all the key partner organizations and agencies. Following the presentation and a question-and-answer session, the participants were again divided into technical teams to seek further input on: (a) data quality, (b) suggested improvements to the presentation, and (3) what the information tells us about the state of the watershed in terms of actionable decisions.

In each of the subsequent years (2018–2021), we have presented the results of this analysis at the State of the Cienega Watershed meeting, the Science on the Sonoita Plain symposium, and in numerous other workshops and seminars. During these opportunities, we have sought additional input, both on the presentation itself (indicators, data quality, presentation of the information), as well as on what the analysis means in terms of implications for management decision-making. The input gained from these many presentations has helped us further refine and adapt the analysis, and the visual presentation of data sets, and has continued to stimulate important conversations about priority management actions within the watershed.

Recent adaptations of the assessment include changes to the list of indicators, based on further evaluation of their utility and/or availability of quality data. Previous assessments were not modified, only the list of indicators to include in future assessments. For example, in 2016 we included prairie dog monitoring. The species is being reintroduced to the grasslands by a team of Arizona Game and Fish Department (AZGFD) biologists, who provide regular feeding and fencing to protect them from predators. However, during the annual meeting that year, the participants agreed that this indicator was not reflective of broader watershed conditions and trends.

Pronghorn antelope is another species that is actively managed within the watershed. If there is a decline in the number of individuals, the AZGFD reduces the number of hunting permits, modifies fences (cutting the lower wire to allow species to cross them), carries out predator control, or transfers individuals from adjacent polygons. Stakeholders agreed that this charismatic species is an important symbol of grassland health and connectivity, so this indicator has been maintained as part of the assessment effort.

Another example is the indicator of number of wells. During the annual workshop in 2017, stakeholders proposed the introduction of wells because groundwater withdrawals directly affect the overall health of the watershed. The following year we introduced this indicator, using publicly available data from the Arizona Department of Water Resources. As a result of discussions among key stakeholders, we decided to include wells within a buffer of 10 mi of the boundary line of the watershed. This decision was made because wells outside the boundary still have impact on the watershed’s aquifer.

Other adaptations include reconciliation of data mismatches as a result of differences in data collection methodologies. Resource specialists in different organizations collect data as part of their regular activities, but each organization has its own process, units, terminology, and methods, and it can be challenging integrating different datasets for the same indicator. To solve these data mismatches, we decided to present these indicators in a parallel way, clarifying differences in nomenclature among the experts.

The development of the annual assessment report also led to improved coordination among agencies monitoring archeological sites in the basin. This coordination focused on how to better evaluate and document the status of significant cultural resources and monitor the trend in site conditions. Since this is a recent development, the effort has not yet resulted in an enhanced ability to detect trends. Nevertheless, it has resulted in a more consistent approach and enhanced collaboration in reviewing site conditions. This demonstrates how efforts to understand the state of a watershed can lead to improved cooperation and coordination among agencies and organizations.

### Communicating results of the assessment

To enhance communication with the wider public, we have made numerous changes in the graphic images used to present the data. We use PowerPoint slides to present the assessment, using color-coded categories (climate, water, ecological, socio-cultural) to guide the audience through the presentation. Where it is possible, we present the data in similar bar graphs, accompanied with a corresponding map to indicate the location where the data was collected. For large scale indicators (e.g., land use, land cover change, wildfires, wells), we rely on geographic information systems to display maps with the data. During each of the annual meetings, we have asked for input on how to further improve the visual display of assessment results and have incorporated this feedback into subsequent iterations.

Each year we received requests to provide a simplified, summary interpretation of the analysis, showing overall trends for watershed health indicators. For this purpose, we adopted an easily recognized evaluation icon to indicate “improvement”, “decline”, or “no change”, using up, down, or sideways hand-thumb images (see Table 3). These icons help stimulate further discussion during the meeting, as they are subject to interpretation and debate. For example, wildfires have increased in intensity and extent in the watershed, but this natural phenomenon can be considered as “positive” (thumbs up) for the grassland ecosystem, but also as “negative” (thumbs down) for riparian species or for recreational infrastructure.

**Table 3:**
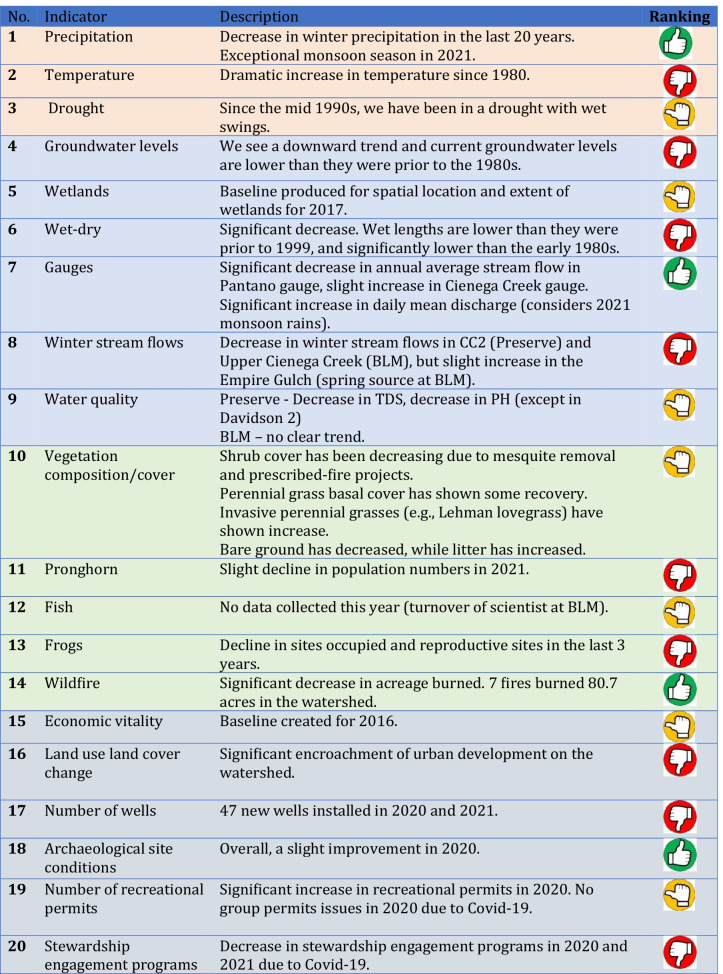
State of the Cienega Watershed — summary of findings (2020–2021)

**Fig. 3:**
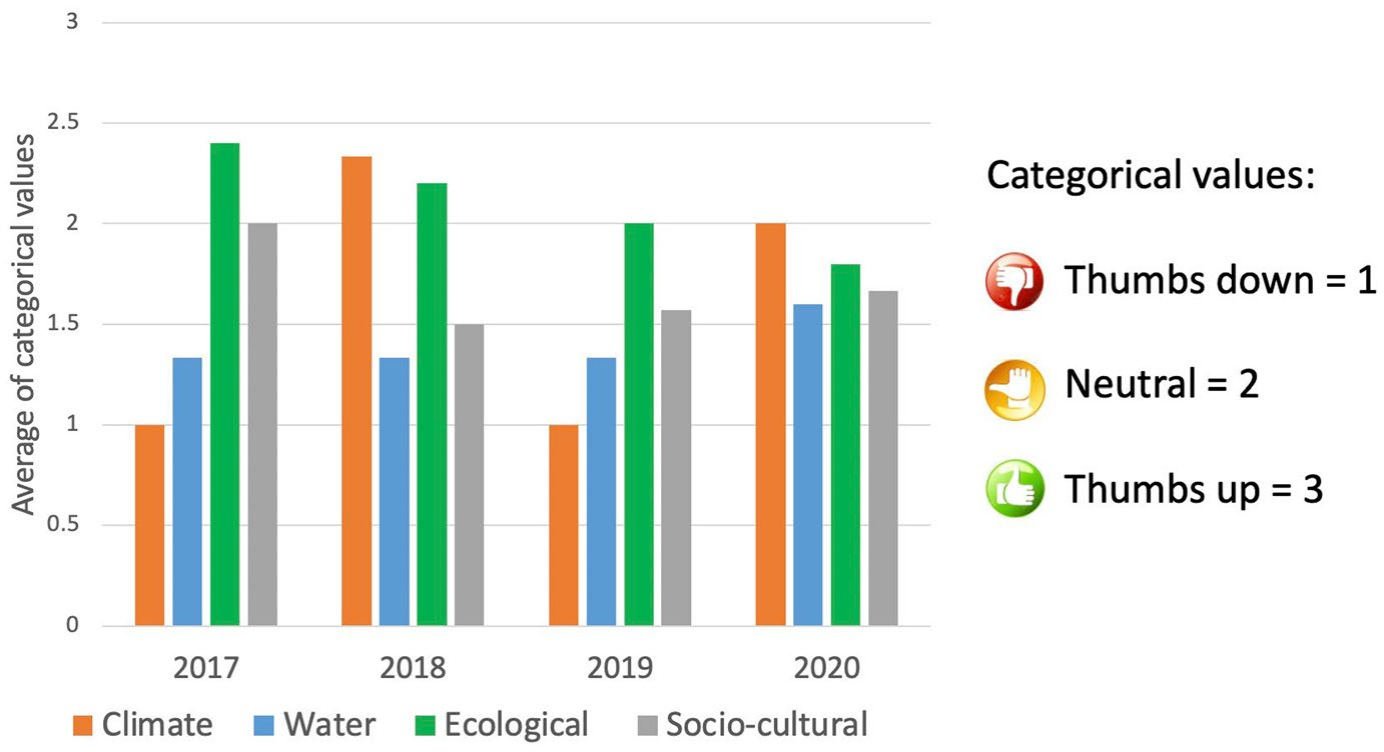
Trend of indicator categories after 3 years of collaborative assessment. Y axis indicates average of categorical values, which are described on the key list (thumps up/sideways/down)

After 5 years of assessment efforts, we identified trends for each category (climate, water, ecological, and socio-cultural) to tease out annual differences (Fig. 3). We used the same familiar ranking (thumbs up, thumbs down, thumbs sideways) to encourage discussion among stakeholders.

We have continued to develop this State of the Watershed assessment over the last 5 years. The assessment has evolved over time as we have gained new insights on how to best measure the indicators and how to succinctly summarize and present the results for the broad set of stakeholders in the watershed and for the public at large. The reporting of the indicators and trends was first completed in 2016. Based on discussion with the participants that year, we determined that a more summative assessment of the indicators was needed than the oral report and accompanying slides.

Each indicator presents its own challenges for summarizing the “state” of the watershed in terms of providing a full picture of what is changing (or not) within the watershed. These challenges include difficulties in summarizing and drawing conclusions about the information used for each of the indicators, but also the limitations of individual indicators in reflecting conditions across the entire watershed. In this section, we discuss the challenges of monitoring the following categories of indicators for the watershed: (a) climate, (b) water, (c) ecological, and (d) socio-cultural (see Table 3 for a complete list of indicators). Then, we highlight some key challenges for watershed health assessment, such as data compatibility and integration, data availability and consistency, challenges with scale, summarizing trends, sustained funding, and the challenges faced when trying to link the assessment to management decision-making.

### Climate

Precipitation provides a good example, since it is a simple, observable metric. But precipitation varies significantly over the watershed, plus there are multiple sources of precipitation data. We evaluated a number of different metrics, ultimately settling on using precipitation reported by the Western Regional Climate Center (WRRC — https://wrcc.dri.edu/wwdt). This archived system permitted independent verification by the participants and enabled a simple and repeatable metric. Similar strategies were used for temperature and drought.

### Water

The water-related indicators presented a different set of challenges. For example, the ideal metric for groundwater conditions would be change in groundwater volume over time. However, average basin groundwater volumes are not generally available for watersheds the size of Cienega Creek. To calculate such a change in volume would require immense data collection and analysis. Instead, we used a set of wells that are regularly monitored on both Pima County’s Natural Preserve and on BLM lands within the LCNCA. A summative metric was then utilized to determine the collective trend in these wells. Nevertheless, there is still uncertainty in these trends since shallow and deep wells have at times shown different temporal trends due to differences in location and the varied frequency of depth to water measurements.

Other water metrics, such as water quality and wetland area, suffer from the relative lack of data across relevant variables. For example, it was not until 2018 that wetland areas in the watershed were precisely mapped. For water quality, while index variables like total dissolved solids (TDS), dissolved oxygen, and pH are available, more relevant metals or nutrient concentrations are not consistently monitored.

**Fig. 4:**
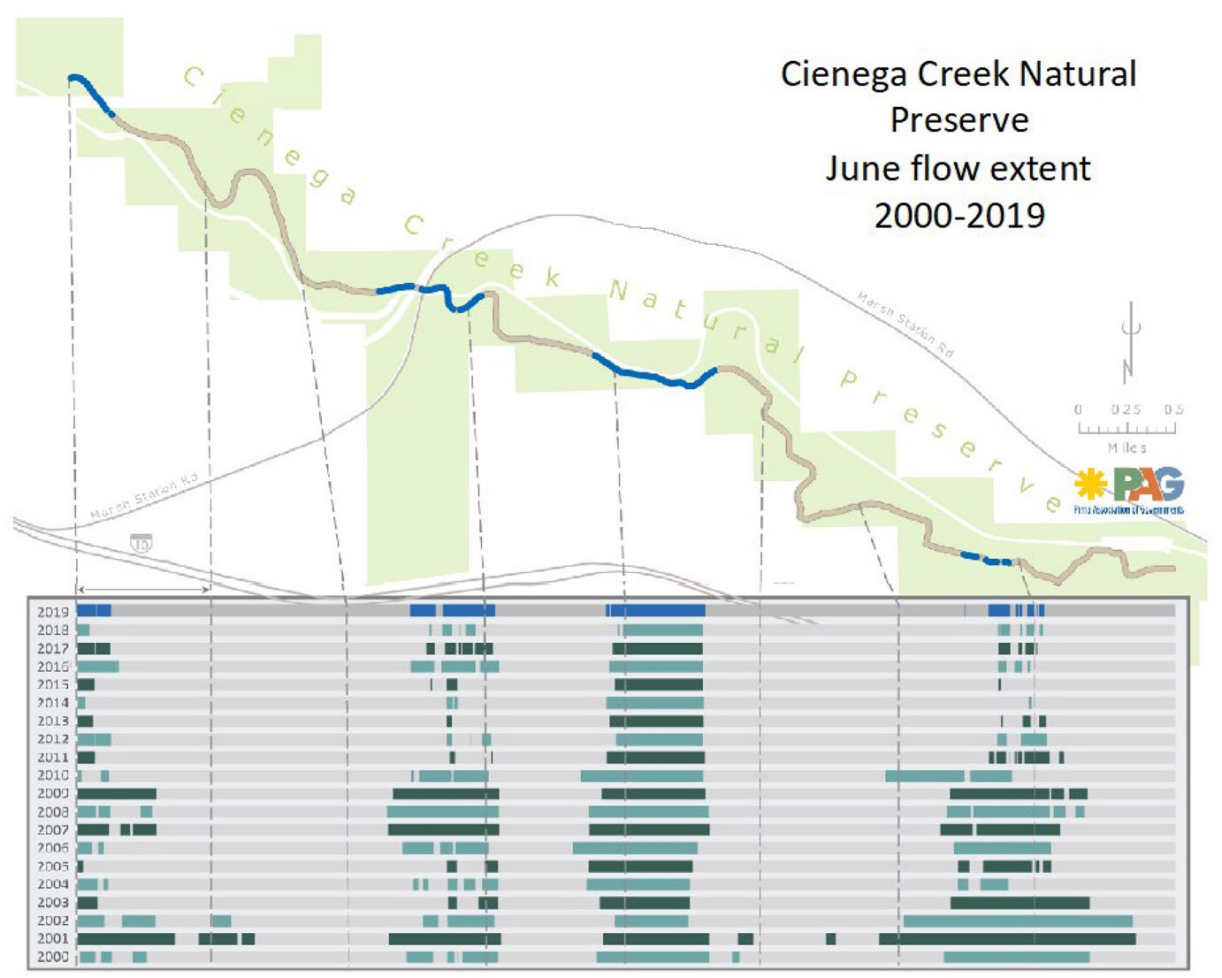
Cienega Creek June flow extent for 2000–2019. June is the driest month of the year, so measuring the flow extent (wet length) in June provides insights on how the creek is shrinking over time ( adapted from Pima Association of Governments’ annual riparian health assessment)

Finally, water data about surface water conditions required multiple metrics. We were fortunate that both the BLM and Pima County support regular “wet-dry” mapping efforts, to determine where streamflow is present and where it is not. These data provide critical insight into stream conditions over time. While surveys are conducted multiple times each year, we have focused on June mapping data, since this is usually the driest time of the year and thus reflects the minimal perennial flow extent — or the length of the creek where there is water running through it. Available maps show a general decline in stream wetted area over time, with some modest rebounds in recent years (Fig. 4). USGS stream gauge data at the two gauges in the basin present a rich data set, but we focused on annual average flow, which represent changes that include peak flow. This approach is important since it helps account for the highly variable climate and hydrologic conditions in the watershed and provides a potential assessment of increased extreme precipitation events under prospective climate change. The data shows a decline in flow for the lower elevation stream gauge but no evident trend for the upper elevation gauge. Many alternative measures might be used to evaluate flow, but since we had low flow data through the wet-dry mapping, and an additional set of sites with monthly data, we chose the more general annual flow statistics for evaluating trends over time.

### Ecological

Similarly, for ecological indicators we struggled with how to reduce the number of indicators while maintaining a broad understanding of watershed health. After several iterations, two landscape-scale measures were retained — land cover, and fire cover and severity. Additionally, we used the status of three endangered species in the watershed — Pronghorn antelope, Chiricahua leopard frog, and consolidated data for three endangered fish species — the Gila topminnow, longfin dace, and Gila chub*.*

Simple observation suggests that there has been substantial land cover change in the watershed, but the underlying data set shows significant year-to-year variability that is likely due more to data collection variation rather than underlying change. Still, over the long term we have seen an increase in the non-native Lehmann love grass. This increase has tracked with decreases in bare ground and other cover types except for perennial grass cover, which has increased. This is perhaps in a large part due to the increase in Lehmann love grass over the time, based on available data.

For fire area, extent and coverage were the metrics of record, and in general, increases in burned area have been observed over the last 40 years. For endangered species, raw counts collected by agency biologists were used for each species. All show increases in recent years, and each demonstrates the value of enhanced management practices. For pronghorn, significant efforts have been put in place to reduce predators (coyotes), alter cattle fencing, and address other landscape-scape aspects to allow for wider grazing and movement of pronghorn. For Chiricahua leopard frogs, a concerted effort has been focused on eliminating the non-native American bullfrogs that prey on the leopard frogs. In contrast to the rebounds of these two species, native fish populations have remained relatively stable.

### Socio-cultural

We also faced challenges in determining appropriate socio-cultural metrics. The availability of zip code and census data for economic vitality did not overlap well with basin boundaries. Nevertheless, we were able to detect a slight decline in economic activity despite population growth on the fringes of the watershed. Similarly, we have seen an increase in developed area but with some data quality issues in the most recent analyses. We also used groundwater wells as a surrogate for development. Construction of new wells has continued, but the rate of construction has slowed in recent decades. Finally, we have yet to identify metrics of change for archaeological sites, but, as noted above, have continued working with partner agencies to develop a systematic and coordinated site assessment approach.

## Key challenges for watershed health assessments

Based on our recent years of experience working on the State of the Cienega Watershed assessment, we have identified several key challenges and lessons that can be applied and/or adapted to other watershed health assessment efforts.

### Data compatibility and integration

In a watershed that comprises multiple jurisdictions, each partner agency and organization may be monitoring the same resource, but in a somewhat different way. Differences can be seen, for example, in frequency, with some agencies gathering data monthly and others only annually. Archeological site conditions are a good example of this problem. The BLM, Pima County, and the US Forest Service all use their own survey forms and criteria to assess conditions of cultural resources. Our effort sought to bring together all the specialists working on cultural resources to design a common procedure for use in their assessment efforts. However, even after achieving consensus, we still encountered challenges in data integration. Their qualitative assessments differed significantly — some used a five-point Likert scale, while others used a seven-point scale. They also used different terms for ecozones. Further adjustments were therefore necessary to integrate data from the three agencies.

Similarly, some of the water monitoring is done at different times for different reasons, resulting in occasional gaps or contrasts in observed trends. Reconciliation of data mismatches remains an ongoing challenge when different organizations monitor the same indicator using different methodologies. Our analysis suggests that continuous stakeholder engagement among partners monitoring the same indicator is helpful in gaining agreement on data collection format, frequency, and metrics. This finding aligns with McLain and Lee (1996) who note that while AM promotes stakeholder collaboration, data reconciliation challenges may arise when drawing from multiple sources.

### Data availability and consistency

As our approach relies on using existing data sources from program partners and other available sources, we have faced numerous problems with data collection methods. This includes differences in both tools and approach, as well as changes in personnel over time. As some projects and contracts end, or individuals in charge of data collection retire or move on to other positions, the assessment effort experiences challenges in data gathering consistency and continuity. For example, The Nature Conservancy (TNC), sponsored by the BLM, had been routinely monitoring vegetation composition at LCNCA, providing data for several years. For the most recent assessment, however, we learned that TNC is no longer working on this project and that BLM will now be monitoring vegetation composition themselves. BLM staff provided us with their data, but it turns out that they are not using the same collection points as TNC; nor are they using the same nomenclature for species categories. Updating, consolidating, and interpreting the data therefore becomes more complicated. For example, we identified an unusual spike in the extent of Lehmann love grass, but it is unclear whether this sudden increase is caused by an actual increase in this invasive species, the change in monitoring methods, or the use of different survey locations.

Similarly, a lack of funding in the late 1980s and early 1990s created a temporary break in available data for mapping wet and dry locations within Cienega Creek. During this unobserved period the length and continuity of the wet stream length decreased dramatically. Given these data gaps, the nature of this decrease and its connection to human or climatic factors is difficult to determine. Our findings highlight the importance of supporting collaborative assessment efforts over the long term to avoid significant data gaps, which has been previously documented, for example by Brownson and Fowler (2020).

### Challenges with scale

We found that several indicators do not conform to the watershed boundary. We therefore sought ways to adapt measurements to the watershed scale. For example, as described above, stakeholders decided that it was important to monitor the number of wells installed in the watershed over time. But wells installed outside the watershed boundary may still impact the water volume within the aquifer. We debated the length of the buffer area and decided, based on input from area hydrologists, to include wells installed within 10 mi (16 km) of the watershed boundary. Similarly, we encountered scale issues with the indicator land use/land cover change. For this indicator, we used a decadal assessment conducted by USGS that tracks large-scale changes in land use and land cover (e.g., from grassland to shrubland). However, this dataset did not capture high-resolution changes in land use related to ex-urbanization. We know from basic observation that ex-urban development is encroaching into the watershed from the nearby towns of Vail and Benson, but these changes are not reflected in the dataset. Therefore, we have sought additional resources to study these changes in the next assessment. Although the watershed scale has been established as the optimal unit for monitoring natural resource conditions (Cohen, 2011), our experience suggests that this scale of analysis may not be appropriate for some indicators, making individual adaptations necessary.

### Summarizing trends

A more recent challenge in the State of the Watershed assessment process has been determining whether a trend is positive (thumbs up), negative (thumbs down), or static/uncertain (thumbs sideways). With some metrics, this judgment is a matter of perspective. For example, a decline in the number of recreation permits can be seen as a negative trend — i.e., the less people visiting trails and camping sites, the less people engaged in preserving the watershed from development. On the other hand, an increasing number of people visiting the watershed may represent the potential for more damage to land and resources — e.g., off-road vehicles impacting vegetation, soils, and litter, increasing risk of fire, graffiti, and looting of archaeological sites.

Wildfire is another example of this problem. Fire events can be considered integral to the ecological functioning of the grassland ecosystem, as they are known to stimulate grass growth and regeneration. However, more recent human-caused fires in the Cienega Watershed have resulted in damage to riparian ecosystems and to infrastructure that are both difficult and expensive to restore. It is therefore somewhat debatable whether an increase in wildfires is a positive or negative impact on the overall state of the watershed. This issue was discussed repeatedly during sessions with stakeholders. The outcome of those conversations suggests that there is a need to establish thresholds in which one indicator shifts from positive to negative or vice versa. For example, it was determined that an increasing number of people visiting the watershed is a positive signal of watershed health, but this may become a carrying capacity issue for land managers (e.g., availability of restroom facilities, parking spots, maintenance costs). Therefore, for at least some of the indicators, it is important to determine appropriate thresholds.

The need to observe the behavior of certain indicators to anticipate sudden changes in ecosystem health is often referred as “sentinels” or “sentinel territories” (Blanchon et al., 2020). The concept is particularly useful when the system faces an imminent threat (e.g., the opening of a mine). Indicators must be sensitive enough to predict major disturbances or even minor stresses (Dale & Beyeler, 2001). Our experience highlights the need to determine these thresholds and predict potential changes to ecosystems, and in this way, gain a better understanding of the overall state of the watershed.

For other metrics, judgment is a matter of weighting. For example, increasing depths to groundwater are an overall negative for the basin as they indicate a loss in groundwater storage at individual wells. However, given the large number of wells within the watershed, the trends are not always consistent. Some wells now show water closer to the surface than they did previously, while others indicate losses of groundwater and increases in depth to groundwater.

Conditions also differ for shallow versus deep wells. It is therefore difficult to draw conclusions, since some locations show worsening conditions while others show improvement. However, comparing progressive annual watershed exit flow at the USGS Pantano gauge with depth to water can also shed light on the overall storage volume of the watershed, The Pantano Dam gauge is significant because it is at the downstream end of the watershed in a shallow bedrock area. So, the exit base flow at the Pantano gauge is a significant indicator of any surplus water leaving the watershed. In addition, the Pantano gauge offers an extended data record (since 1960) and is therefore useful for comparison of drier (1960–64, 1994–2000, 2014–2020) and wetter periods (1977–1993, 2006–2014). Finally, a clear relationship has been established between depth to groundwater and length of perennial flow in the lower Basin, and to a lesser extent within the upper Basin. More data is needed to explore the upper Basin relationship. But extent of streamflow is also important to evaluate and compare when looking at depth to groundwater over the watershed as a whole.

Groundwater results are indeed complex and exemplify this challenge. While broad conclusions are difficult to draw for the entire watershed, more discrete trends about specific systems can be teased from more detailed data. This assessment effort helps to signal managers to take a closer look at the causes and impacts in each subregion. This finding aligns with He et al. (2000), who point to differences in the spatial and temporal distribution of hydrological and biophysical processes that influence watershed conditions.

### Sustained funding

The most difficult challenge to address in a long-term monitoring effort is the need for continuous, sustained funding. Monitoring is often regarded as a low priority activity that is often supported only episodically. Declining federal budgets have further exacerbated this tendency, and in a direct sense resulted in a reduction of support (in this case, from the BLM) for the compilation of the State of the Cienega Watershed assessment report. We have been fortunate to secure other external sources of funding, including support from the US Geological Survey, and the French research center — *Centre National de la Recherche Scientifique* or CNRS, but both of these sources can only commit to limited funding cycles (2 years), which means an uncertain future for what should be a routine and sustained annual assessment. Although the financial challenges for long-term monitoring in AM projects have been well documented (Brownson & Fowler, 2020), this case again demonstrates the need to engage with a large number of stakeholders, including universities, in order to gain access to a more diversified and resilient funding portfolio that can sustain AM efforts.

### Linking the assessment to management decision-making

Ultimately, the true test of success within the framework of an adaptive management effort is a robust, ongoing connection between analysis of monitoring data and management decision-making. CWP recognized from the start that stakeholder engagement in the development of the watershed assessment would encourage more active involvement in reviewing these trends over time. The annual State of the Watershed meeting provides a regular forum for review and discussion of assessment results and has continued to engage a wide range of stakeholders from key agencies and organizations. During each iteration, we have continued to make adjustments to the assessment tools and approach, eliciting lessons learned and identifying implications for land management.

Nevertheless, while the response to declines of individual species (Chiricahua leopard frogs, pronghorn antelope, native fish species) has shown unequivocal success, and the growing collaboration over cultural heritage sites represents a significant development, response to the more chronic and complex issues of water availability and land cover continues to present challenges. This may be due as much to natural causes (climate change, invasive species, fire) as to internal agency dynamics (staff changes, shifting priorities and policies, budget constraints) and ongoing development in the area. CWP must continue to find ways to leverage the assessment results to mobilize stakeholder response and increase public awareness about these issues, so that this awareness can be translated into practical programs that address the concerns reflected in the trend data. An increased understanding of trends in watershed health has the potential to lead to better informed management decision-making.

How can an AM approach address these concerns? As one example, winter precipitation is decreasing, while summer flooding intensity has increased, but this has not resulted in much additional storage. Upland detention/retention is therefore recommended to minimize summer flash floods and reduce water loss within the watershed. Non-structural methods mimicking site hydrology, including preserving natural areas and using small scale berms and check dams in first order tributary channels can certainly help. Erosion has become an issue due to increased summer flood intensity and decreased winter rains that provide antecedent moisture to grow grasses and shrubs, typically retaining greater water volume. For these reasons, stakeholders agreed to focus greater attention on erosion control efforts. Some areas of the Creek are now able to partially detain flows with woody debris. However, some of the Creek has been adjusting to loss of water levels below the Creek by head cutting. Modeling and actual pilot studies of proposed adjustments to upstream areas may reveal, if further actions can help.

## Summary and conclusions

In this study, we offer a broad an exploratory approach to addressing the critical question of *practice* of AM. Using a case study approach, we analyze the State of the Cienega Watershed in southern Arizona, to examine (1) the challenge of selecting indicators, (2) the negotiation process between stakeholders around the list of indicators and their interpretation, and (3) communicating the results of the assessment. The multi-year effort to develop a State of the Watershed assessment for the Cienega watershed has resulted in improved understanding of the state and direction of change in the watershed and enhanced understanding of how management can affect that status.

We list the key contributions of the paper in the form of five lessons learned. First, given complex jurisdictional and community dynamics, assessing overall watershed health requires a detailed process for seeking agreement over what stakeholders consider most important.

Second, the process of cataloging those indicators will necessarily lead to shifts in the metrics used and may mean that aspects the community initially considered important cannot be monitored with existing data availability.

Third, a drive to create simple communication around metrics of improvement or decline in watershed health can be quite nuanced. While some metrics are simple (e.g., increasing population numbers an endangered species is good), others are not as straightforward (e.g., the pros and cons of increased recreational use). Such challenges point to some metrics being subject to the need to identify thresholds (e.g., some fire is good, but not if so extensive that it results in ecological damage).

Fourth, the process of identifying the need for a metric can foster improved cooperation between agencies, as we have seen with the cultural site assessment process.

Finally, the assessment process itself can enhance communication, collaboration between partners, and analysis of what is most important in the watershed, providing new opportunities for agencies and stakeholders to adapt management decision-making to improve watershed health. Ultimately, a comprehensive, broadly supported assessment effort has the potential to speak not only to land managers, but also to public constituencies and agency decision-makers.

## Supplementary Information

Below is the link to the electronic supplementary material.

Supplementary file1 (PDF 9962 KB)

## Data Availability

The data compiled for the State of the Cienega Watershed in 2020 is included as electronic supplementary material.

## Footnotes

1 See: https://www.basinandrange.org/basin-and-range-blog/blm-partnerships-receive-partners-in-conservation-awards).

2 A HUC 10 or fifth-level watershed typically covers an area of 40,000 to 250,000 acres (16,000 to 100,000 ha).

3 The Rosemont Mine — a copper mining operation proposed by Canada’s Hudbay Minerals Inc. — was initially approved by the US Forest Service but subsequently overturned in the Federal District Court in August 2019. The company has vowed to appeal the decision, despite continued oppositions from an array of environmental advocates and citizens’ groups.

4 The session included presentations from the Santa Cruz Living River Report, San Pedro Sustainability Report, ADWR Water Atlas Vulnerability Assessment, BLM’s Assessment, Inventory and Monitoring (AIMS) initiative, USDA Forest Service’s Watershed Condition Framework and the Collaborative Forest Landscape Restoration Program.

5 Of the 40 responses, 11 (27.5%) were from federal agencies, 9 (22.5%) from NGOs, 5 (12.5%) from universities, 3 each (7.5%) from local businesses and from county agencies, 2 each (5%) from state agencies and ranchers, and 5 (12.5%) from others.
