# Supplementary material for: Stakeholder participation, indicators, assessment, and decision-making: applying adaptive management at the watershed scale
Source: Environ Monit Assess. 2022 Feb 8;194(3):156. doi: 10.1007/s10661-021-09741-4 (PMC8821513; doi:10.1007/s10661-021-09741-4)

State of the Cienega Watershed

September 18, 2020

Presented by Adriana Zuniga-Teran

Created by Larry Fisher – UA/CWP, **Tom Meixner** – UA/CWP, **Adriana Zuniga-Teran** – UArizona, **Shela McFarlin** – CWP, **Tahnee Robertson** - SW Decision Resources/CWP, **Frank Postillion** – CWP/Pima County Reg. Flood Control District

Overview

- Introduction
- Objective
- Methodology
- Results
  - Climate
  - Water
  - Ecological
  - Socio-cultural
- Conclusion
- Next Steps

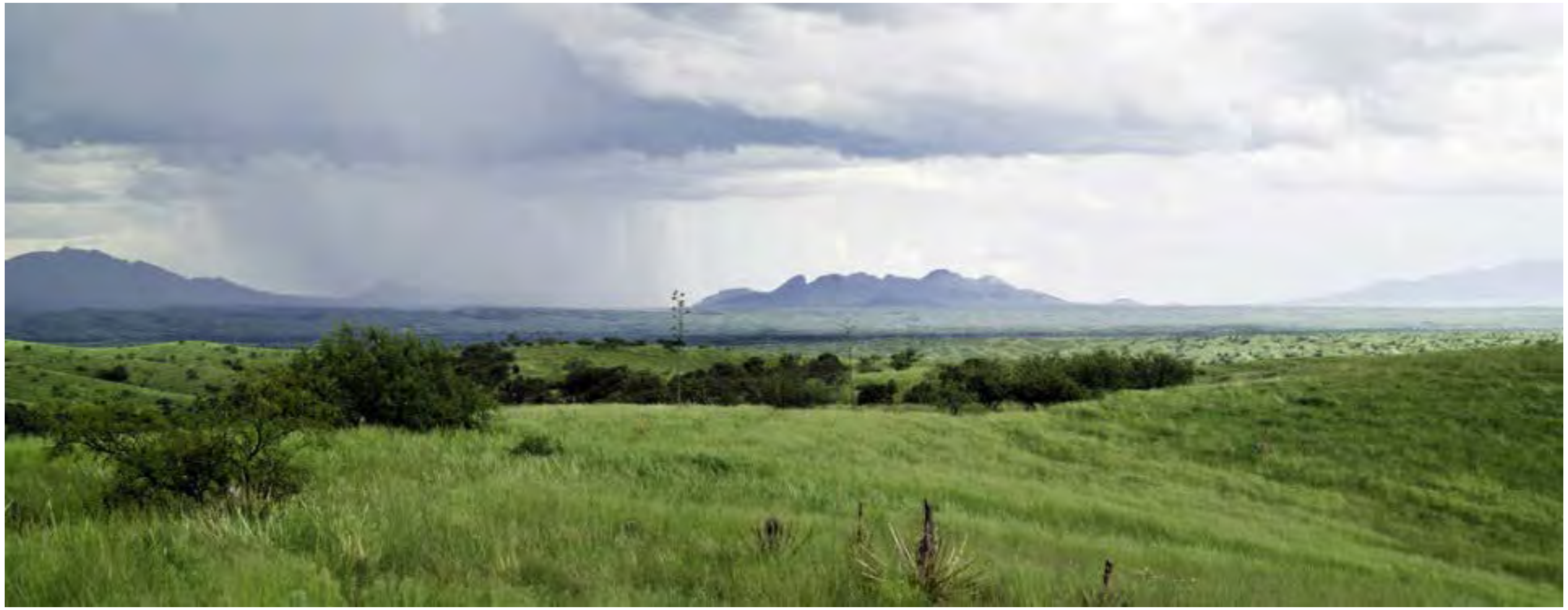

Cienega Watershed

- Includes five of the rarest habitat types in the American Southwest:
  - Cienegas (marshlands)
  - Cottonwood-willow riparian forests
  - Sacaton grasslands
  - Mesquite bosques
  - Semi-desert grasslands

- Cienega Creek – one of the few remaining perennial streams in Arizona, providing critical habitat for wildlife (threatened & endangered species).
- Historically important ranching operations, important cultural/archeological resources.
- Attractive visitor destination, for its scenic landscapes, natural beauty, and cultural heritage.
- Water source for Tucson Metropolitan Area (groundwater).

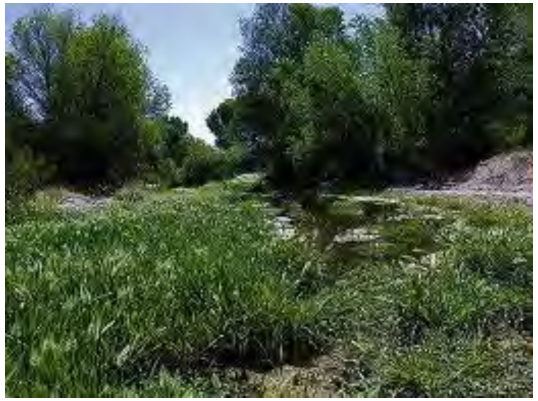

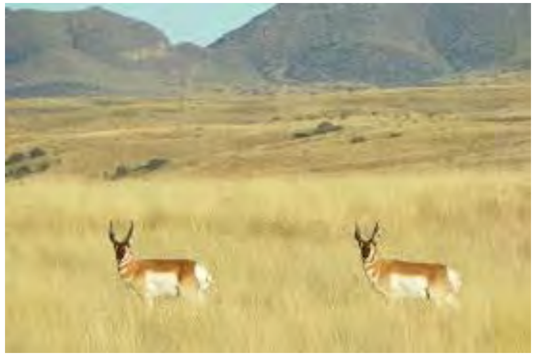

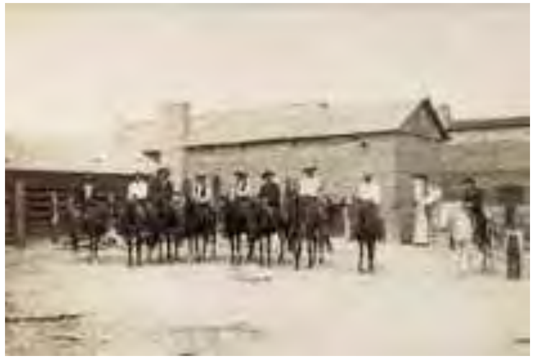

Collaboration & adaptive management

- Broad-based partnership effort to protect a landscape threatened by fragmentation and development – includes federal, state, and county agencies, NGOs, ranchers, researchers)

- Has become a national example of effective stakeholder engagement and adaptive management

- Management approach includes strong emphasis on:
    - Shared goals, measurable objectives
    - Relevant, reliable scientific information
    - Mechanisms for incorporating new information into decision making
    - Shared learning to improve both process and management actions

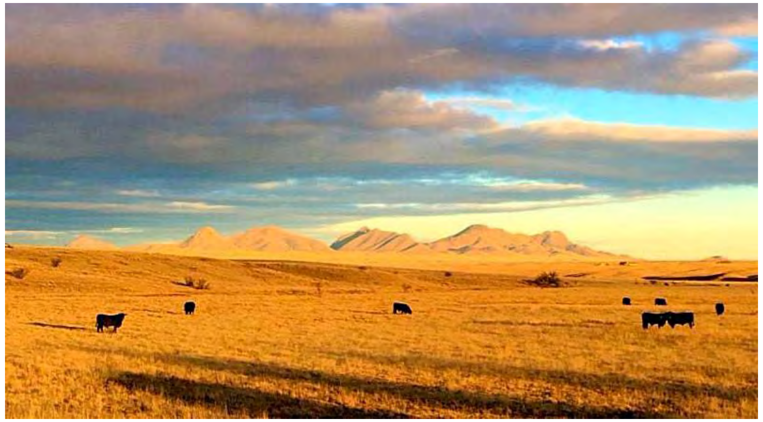

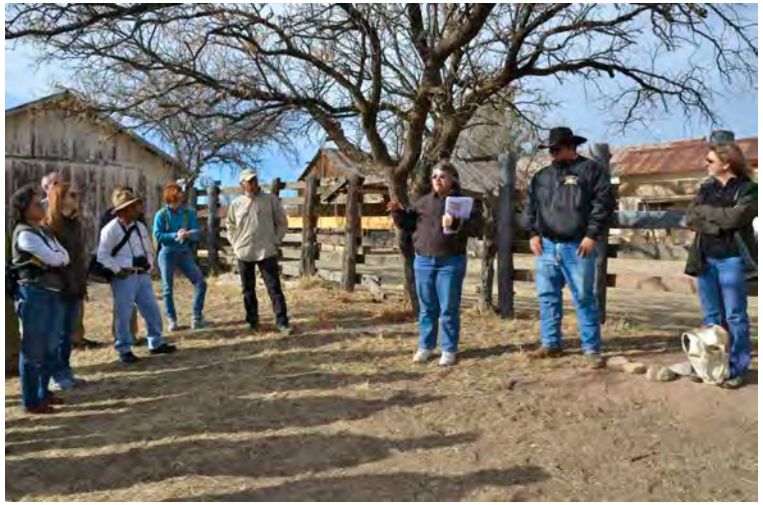

Management challenges

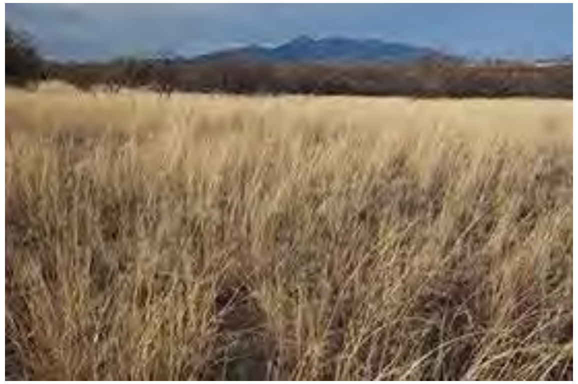

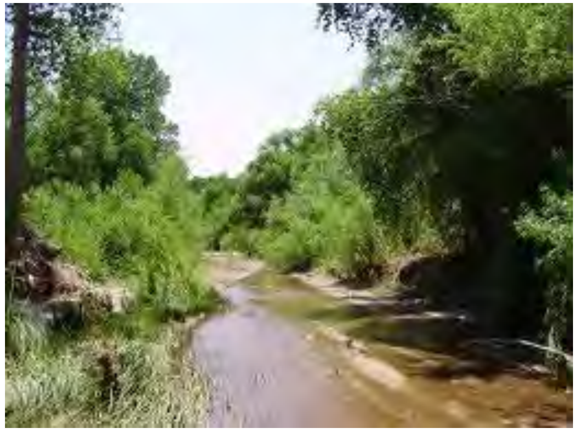

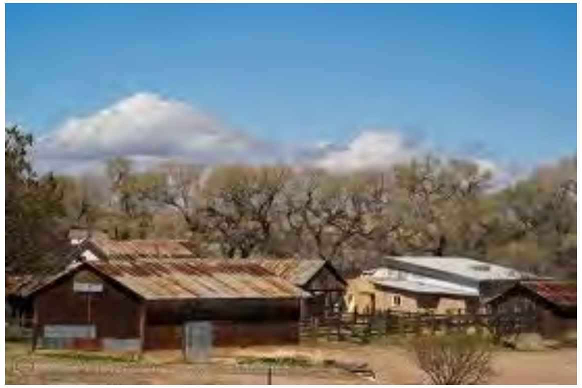

- Dewatering of Cienega Creek and wetlands through development, mining, and climate change
- Threatened/endangered species
- Spread of invasive species
- Restoration of critical habitat
- Connectivity to larger landscapes
- Loss of cultural heritage sites
- Declining federal budgets

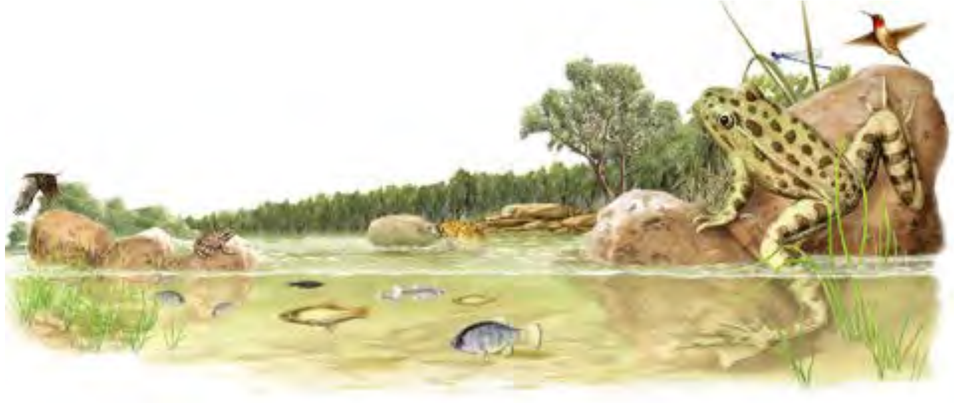

Cienega Watershed Partnership

The Cienega Watershed Partnership (CWP) is a non-profit organization that supports stewardship initiatives in the Cienega Watershed.

CWP works with many partners to sustain the ecological systems, heritage values, wildlife corridors, and open spaces for future generations.

The State of the Watershed is part of a larger effort to provide a regular assessment of our watershed's health, capitalizing on our partners' existing data, and providing a additional mechanism for long-term monitoring, regular evaluation, and adaptation of CWP program priorities and actions to meet changing conditions.

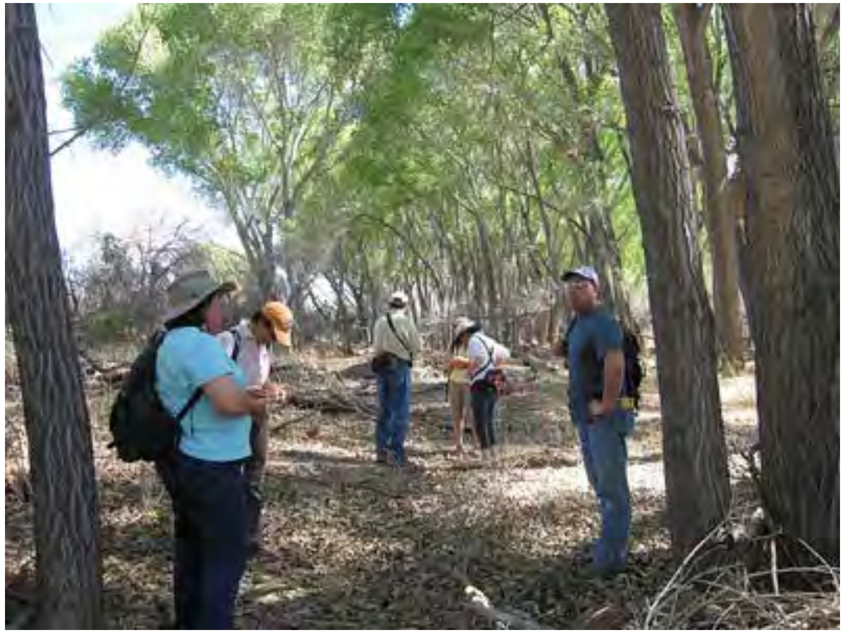

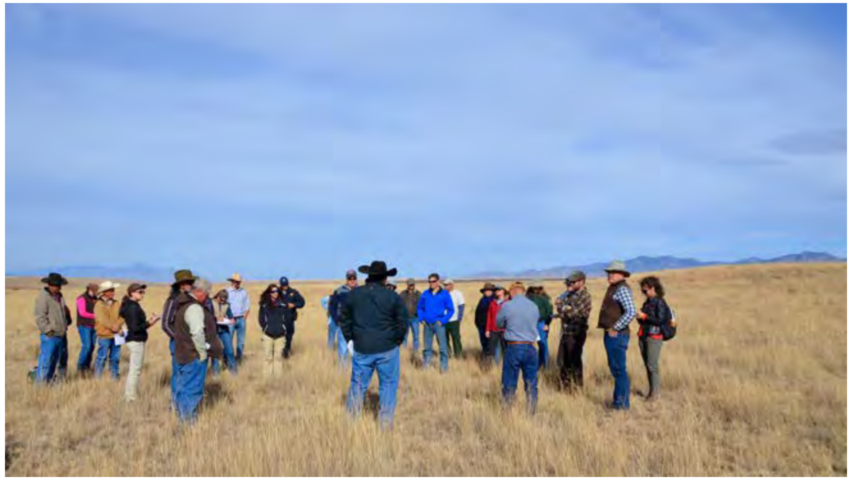

Objectives

- Monitor the state of the Cienega Watershed through a common set of overarching indicators
- To provide a regular mechanism for evaluating watershed health
- Communicate this assessment to program partners and the community at large
- Guide the implementation and adaptation of CWP program priorities and actions to meet changing conditions.

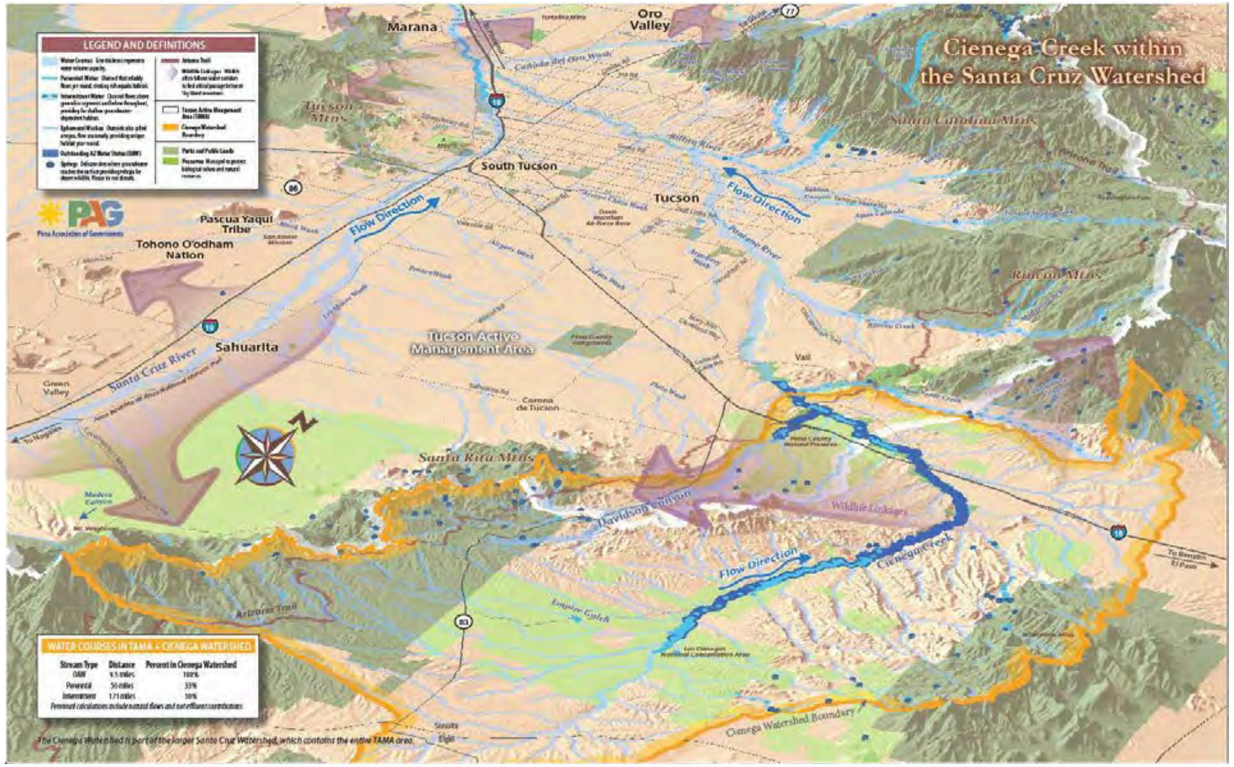

Source: Pima Association of Governments

Methods and approach

- Initial tasks
  - Develop criteria for evaluating indicators
  - Identify and prioritize indicators
  - Identify sources of data
  - Determine appropriate ways of presenting the results
- Three plenary workshops with CWP partners
- Periodic meetings of four working groups
  - Landscape
  - Riparian/water
  - Uplands
  - Social/cultural

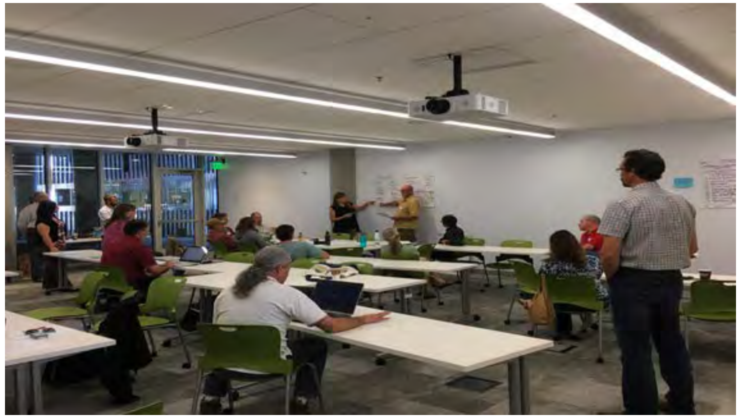

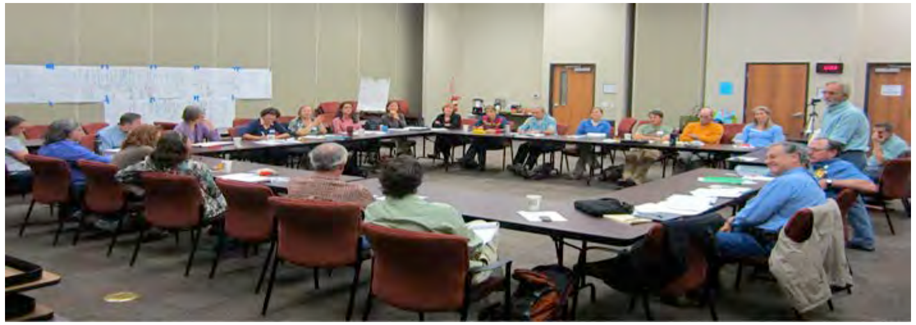

Methods and approach (cont.)

- Electronic survey distributed to CWP partners (R = 40/84)
- Extensive contact with key partners to gather available data, determine how best to communicate the data, and ask where to find missing data, or identify suitable proxy data
- Periodic meetings to further refine data analysis and presentation

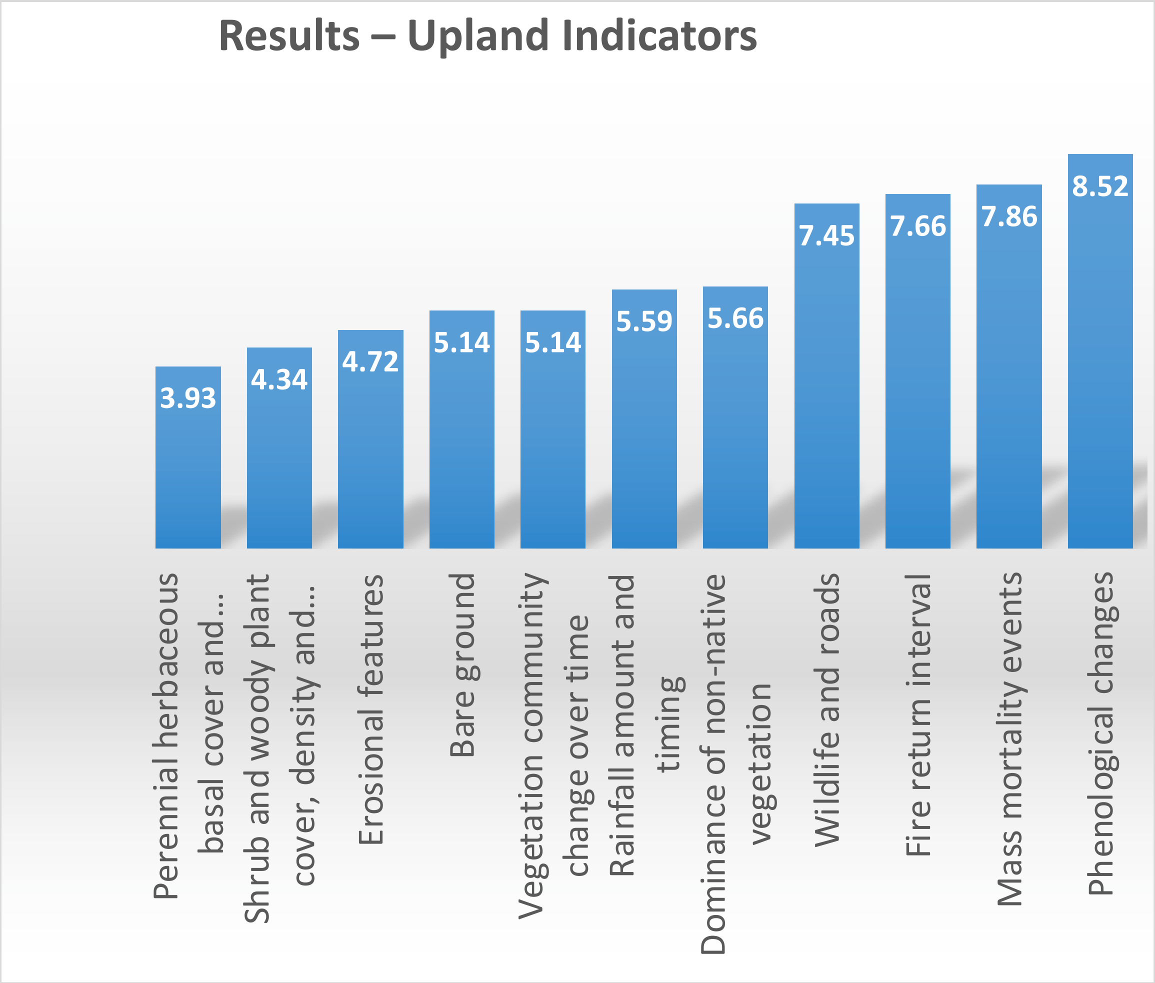

Cienega Watershed

Hydrologic Unit Codes (HUC) 10

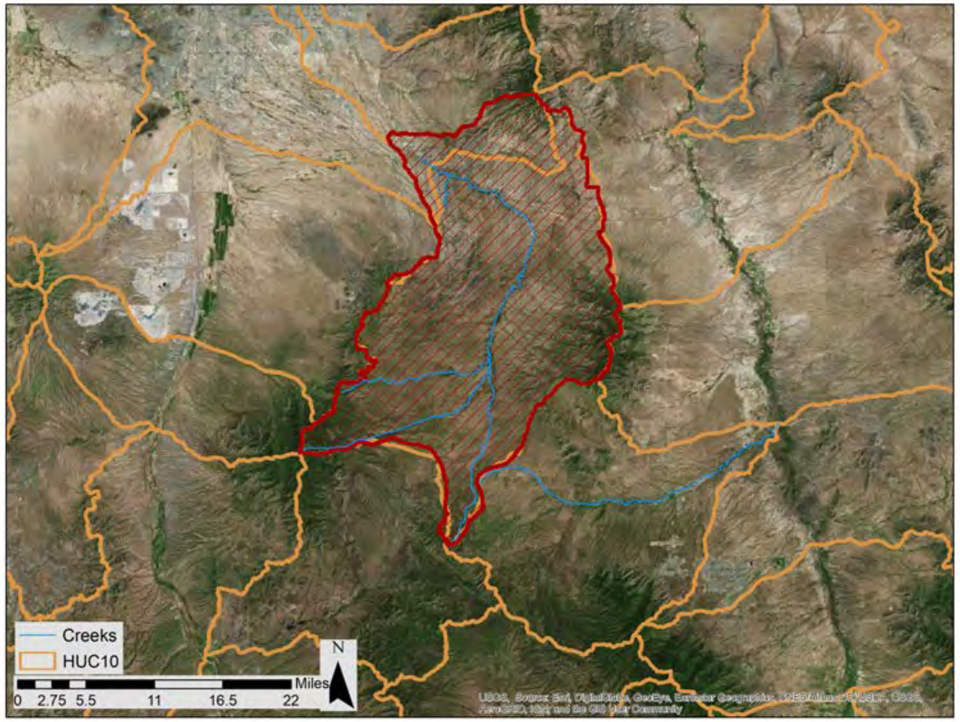

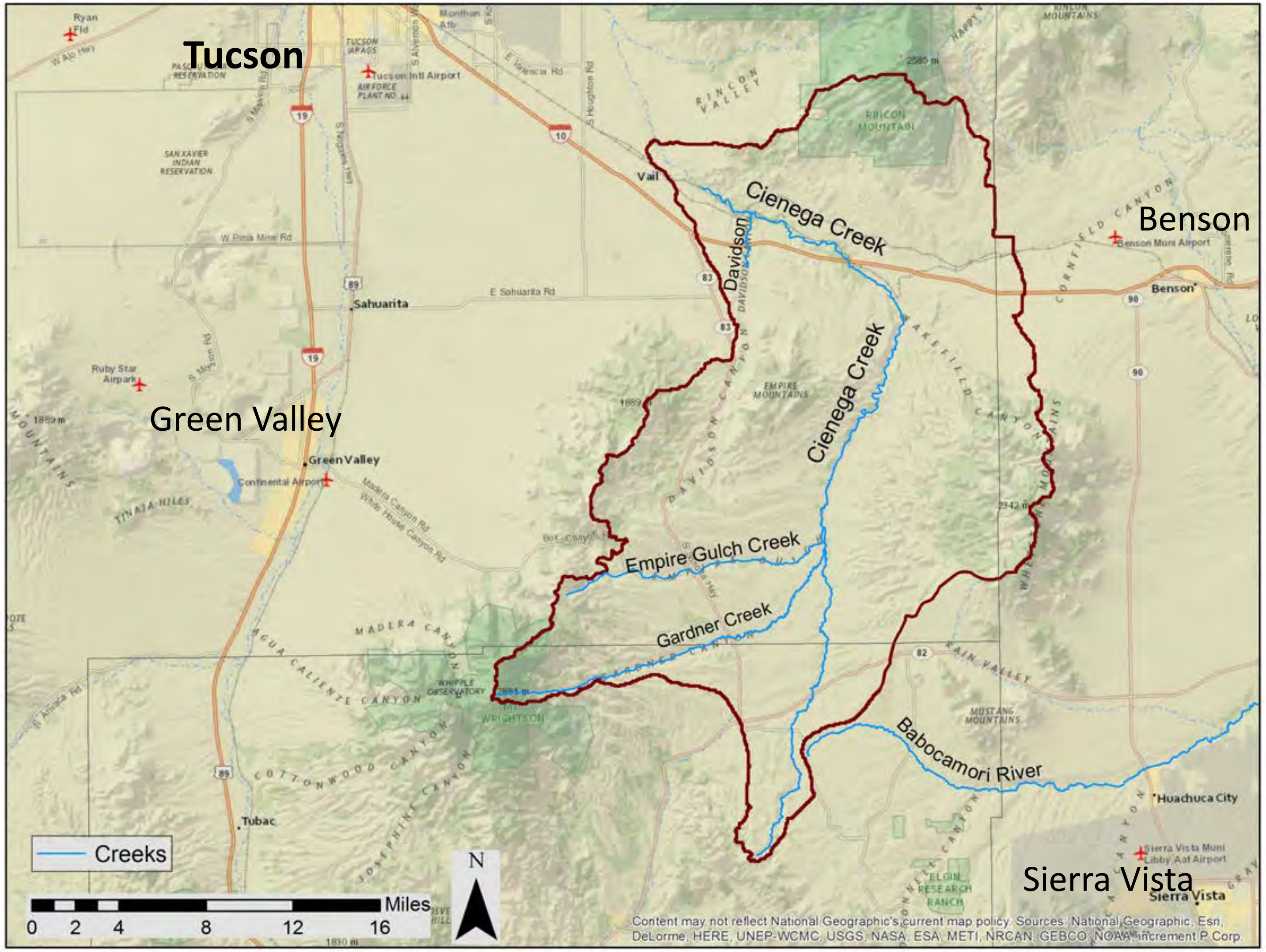

Boundary:

- Shapefile provided by Brian Powell from Pima County.
- Partially corresponds with USGS – NHD_HUC 10.

Hydrologic Unit Codes (HUC) 10

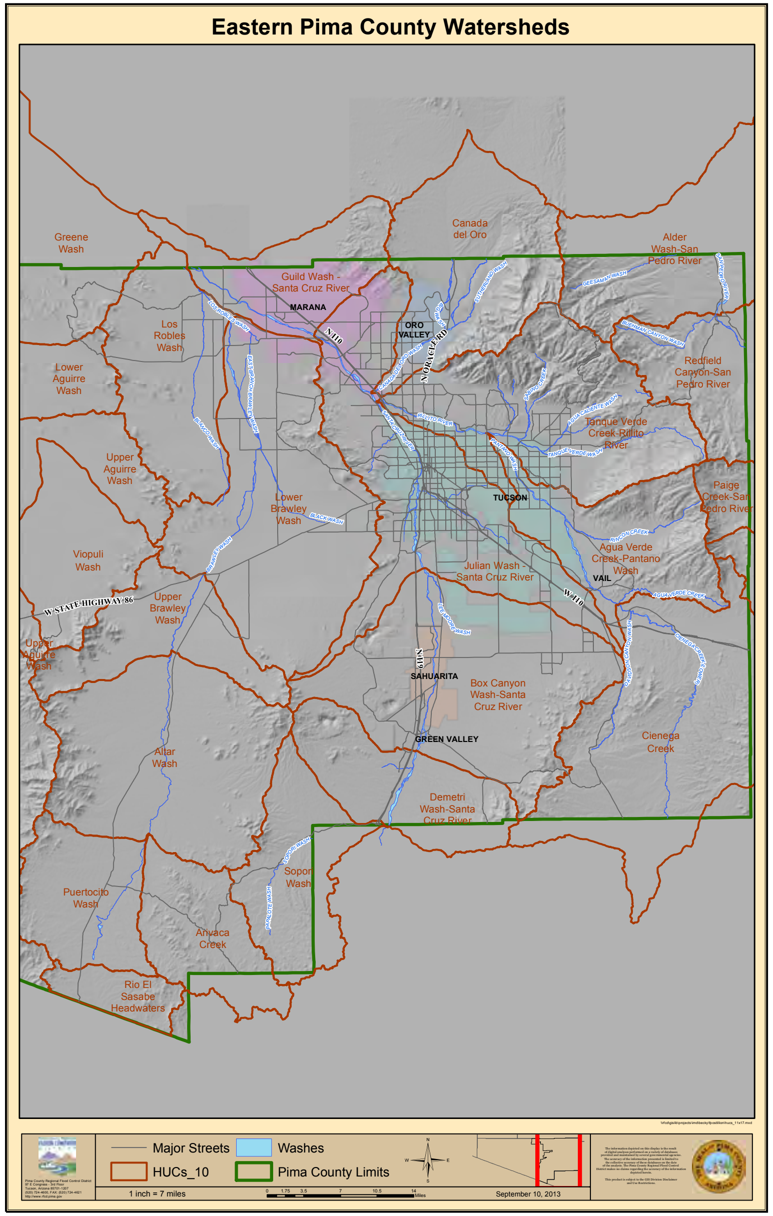

Cienega Watershed

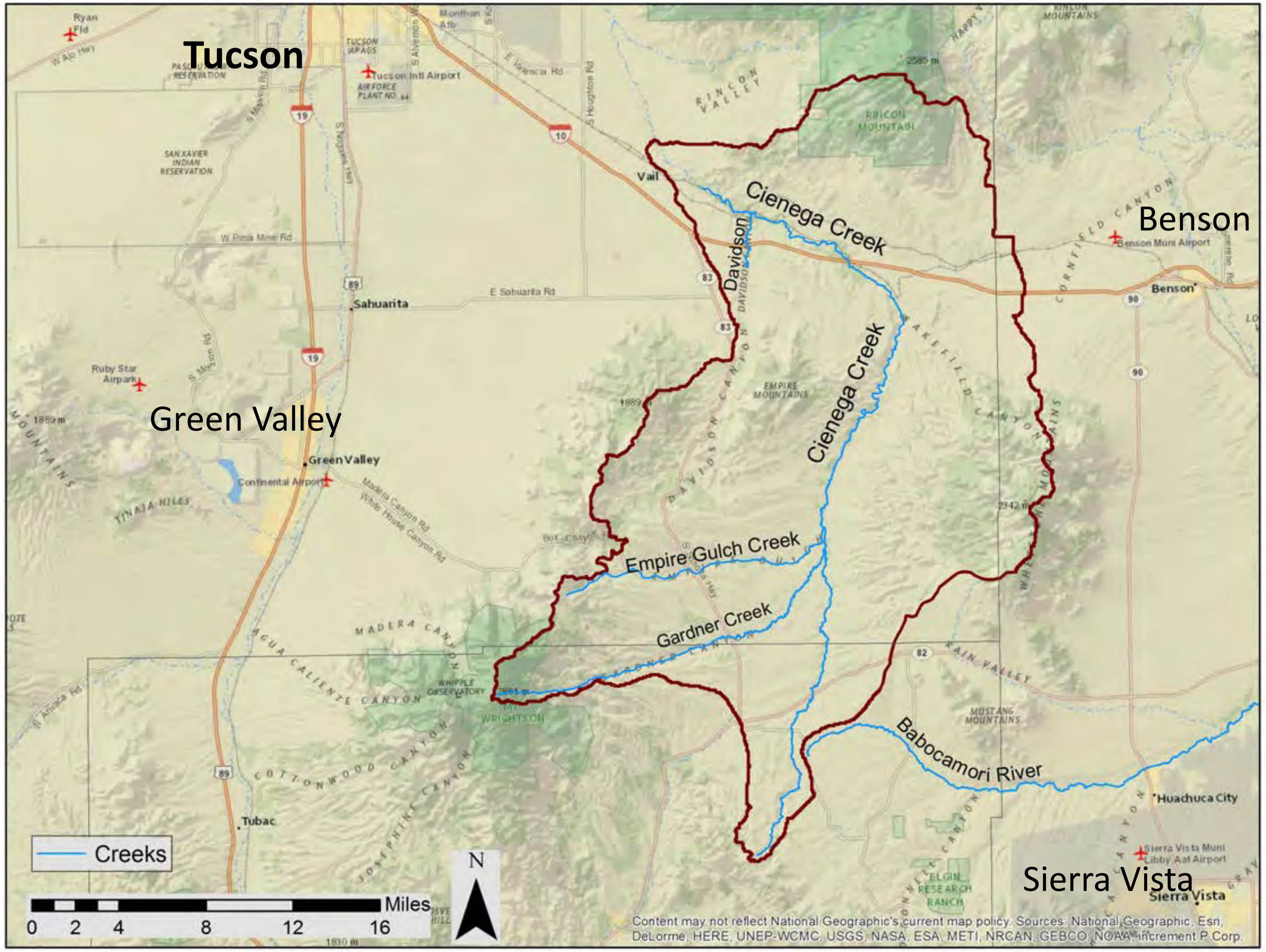

Land Ownership

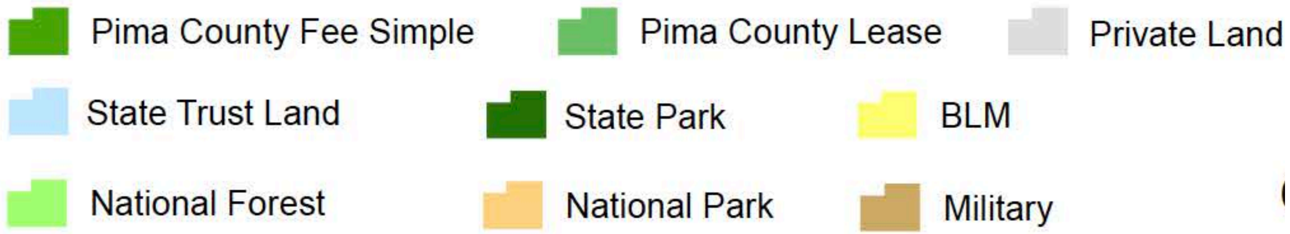

Source: Mike List from Pima County

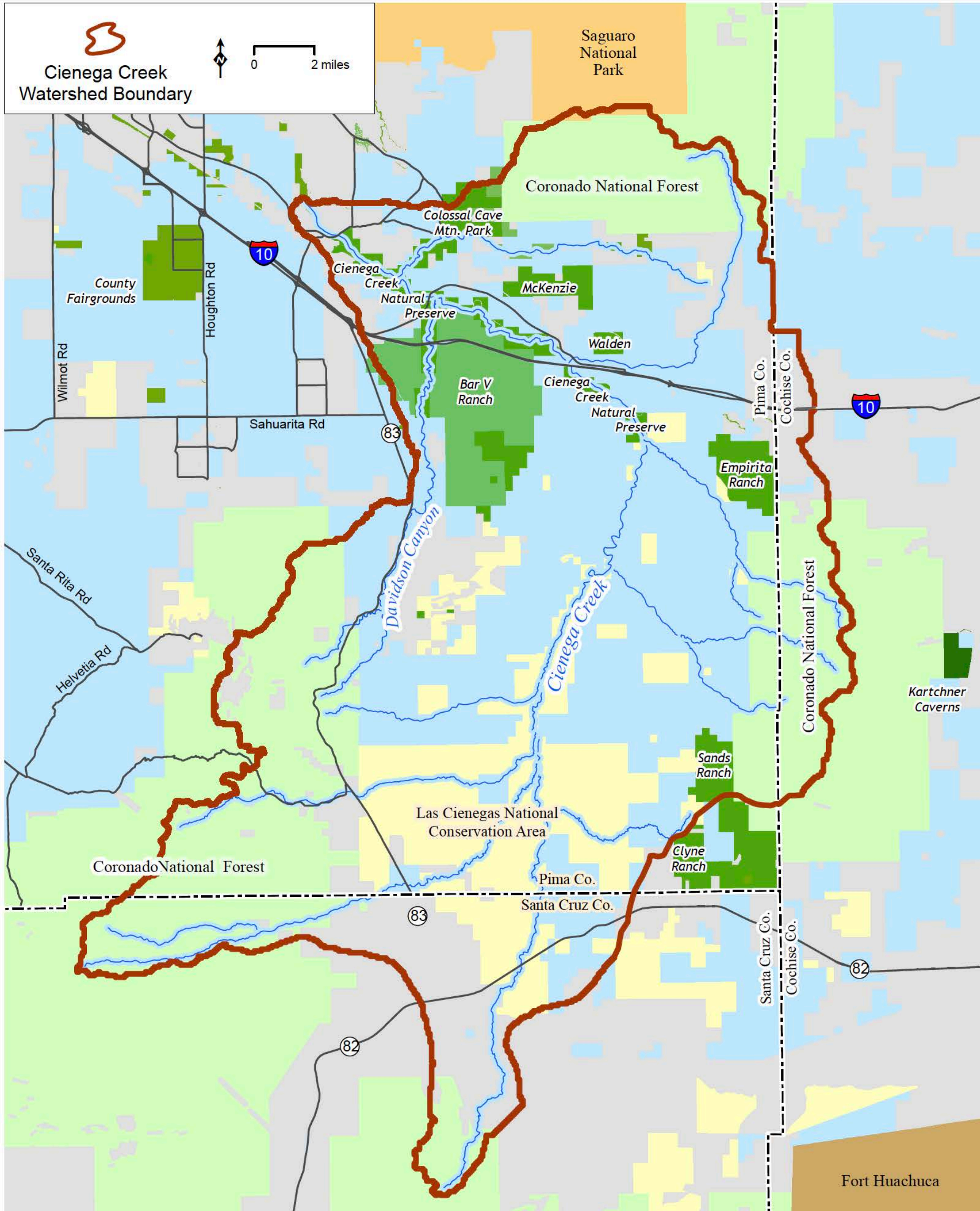

List of Indicators

Category	Indicator	No.	Description
Climate	Precipitation	1	Historic data on mean precipitation summer vs. winter
	Temperature	2	Historic data on mean temperature
	Drought	3	Standardized Index for drought over time
Water	Groundwater levels	4	Change of groundwater levels over time
		5	Wetlands – spatial location and extension of wetlands
	Surface water quantity	6	Wet-dry mapping (June –worst case)
		7	Gauges (Narrows and Pantano Dam)
		8	Monthly Flows/ base flows
	Water quality	9	TDS, dissolved oxygen (fish), PH
Ecological	Veg. volume/composition/cover	10	Land cover
	Wildlife	11	Pronghorn
		12	Fish
		13	Frogs
	Fire	14	Coverage
Socio-cultural	Economic vitality	15	Median household income, median home values, unemployment, residents below poverty level
	**Population density and growth**	**16**	**Changes in population density according to Census data**
	Land use land cover change	17	Land use and land cover change
	Number of wells	18	Number of wells installed within the watershed and buffer area of 10 mi
	Archaeological site conditions	19	Trend in site conditions, both human and natural-caused damage.
	Number of recreational permits	20	Number of recreational permits over time
	Stewardship engagement programs	21	Number of opportunities for active engagement

N=21

Acknowledgments

**Adams, MaryAnn**	Pima County Parks and Recreation
**Alvarez, Melanie**	Pima Association of Governments
**Behrend, Matthew**	Arizona State Land Department
**Bodner, Gita**	The Nature Conservancy
**Buntin, Alison**	Empire Ranch Foundation
**Burns, Emily**	Sky Island Alliance
**Burroughs, Wendy**	Pima County Natural Resources
**Crimmins, Michael**	University of Arizona
**Condo, Theresa**	Bureau of Land Management
**Driscoll, Zach**	Bureau of Land Management
**Gicklhorn, Jeff**	Pima County
**Hall, David**	CWP- Frog project
**Hartfield, Kyle**	Arizona Remote Sensing Center
**Lamb, J.J.**	Vail Preservation Society
**List, Mike**	Pima County
**Le Tourneau, F-Michel**	iGlobes, CRNS
**Mehalic, Dave**	US Forest Service
**Mendoza, Francisco**	Bureau of Land Management
**Mier, Mead**	Pima Association of Governments
**Monkemeier, Peggy**	Bureau of Land Management

**Murray, Dave**	Bureau of Land Management
**Murray, Ian**	Pima County
**Norman, Lara**	US Geological Survey
**Pere, Christina**	Bureau of Land Management
**Postillion, Frank**	Pima C. Regional Flood Control District
**Powell, Brian**	Pima County
**Quintana, Dan**	Bureau of Land Management
**Rose, Courtney**	Pima County
**Ryan, Kim**	Bureau of Land Management
**Salywon, Andrew**	Desert Botanical Garden
**Scalero, David**	Pima C. Regional Flood Control District
**Schrager, Chris**	Bureau of Land Management
**Simms, Karen**	Pima County
**Simms, Jeff**	Bureau of Land Management
**Tiller, Ron**	Desert Botanical Garden
**Tucker, Rana**	Arizona Game and Fish Department
**Van Leeuwen, Wim**	Arizona Remote Sensing Center
**Walter, Robert**	Bureau of Land Management
**Zazueta, Noemi**	Pima County Parks and Rec

Climate

Category	Indicator	No.	Description
Climate	Precipitation	1	Historic data on mean precipitation summer vs. winter
	Temperature	2	Historic data on mean temperature
	Drought	3	Standardized Index for Drought over time

Climate

- **West Wide Drought Tracker** offers a pixel representation of climate data and drought indices at all time scales.
- Includes point data from rain gauges, which drives the creation of gridded climate data (interpolation/statistical estimate).
- Accounts for elevation.
- It is a defensible way to track climate.

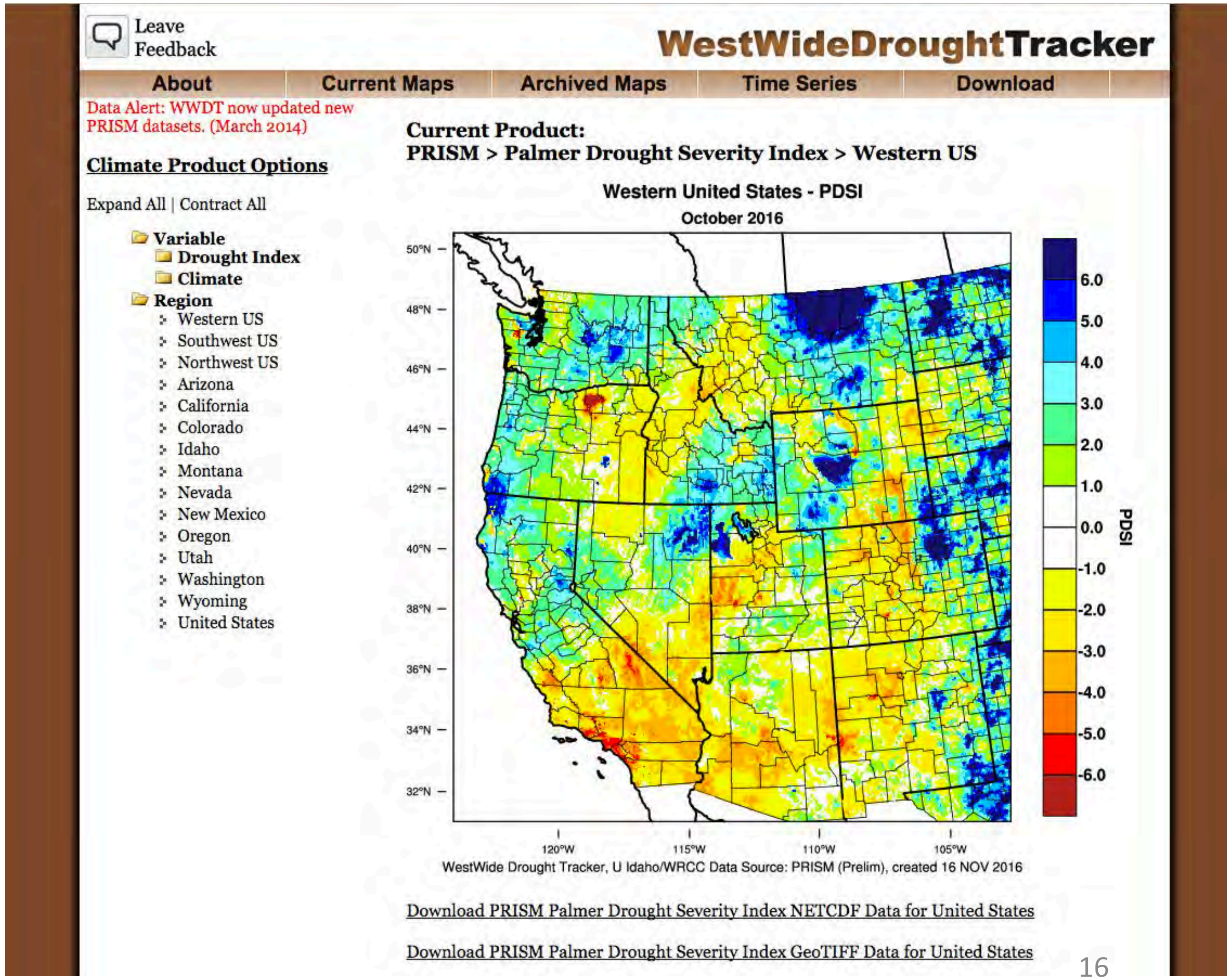

Source: http://www.wrcc.dri.edu/wwdt/ (click Time Series tab)
Advise from: Mike Crimmins

Precipitation

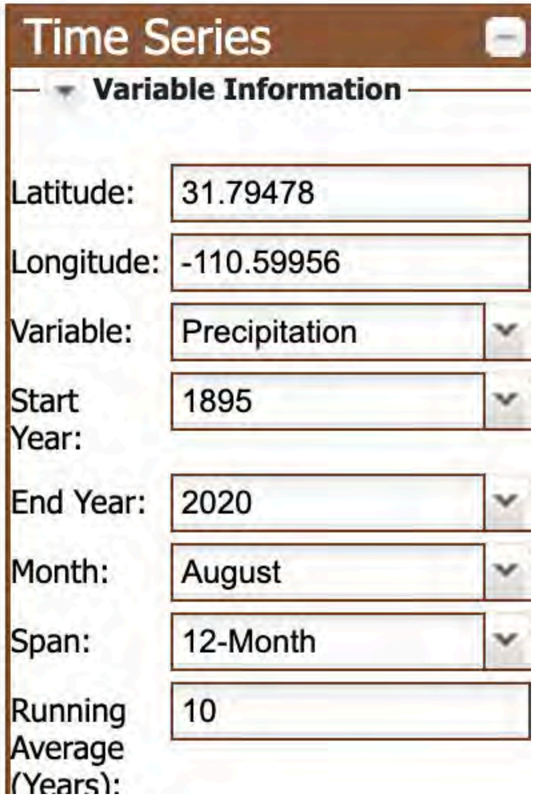

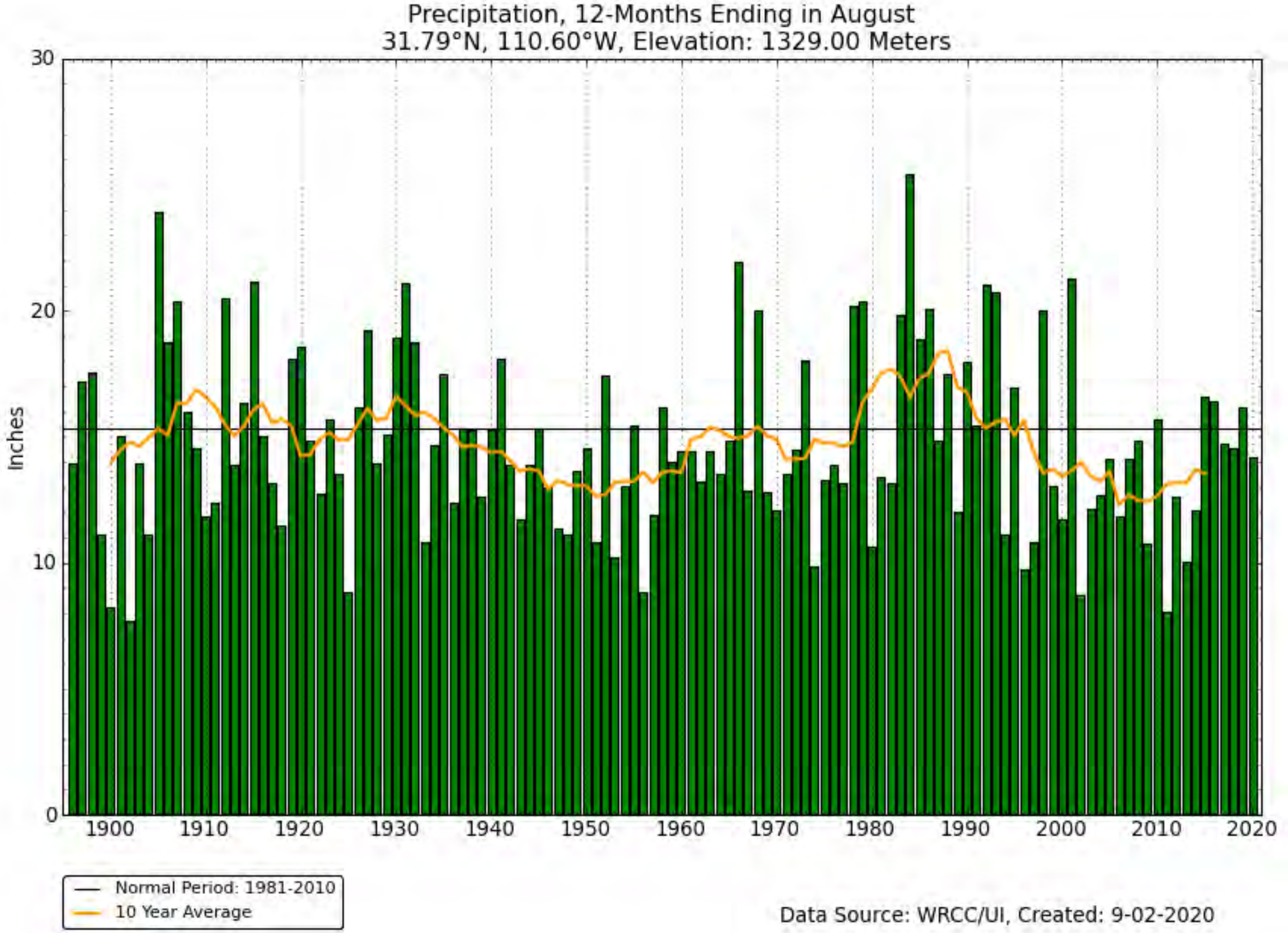

Source: http://www.wrcc.dri.edu/wwdt/
Assistance from: Mike Crimmins

Precipitation – summer vs. winter

Summer

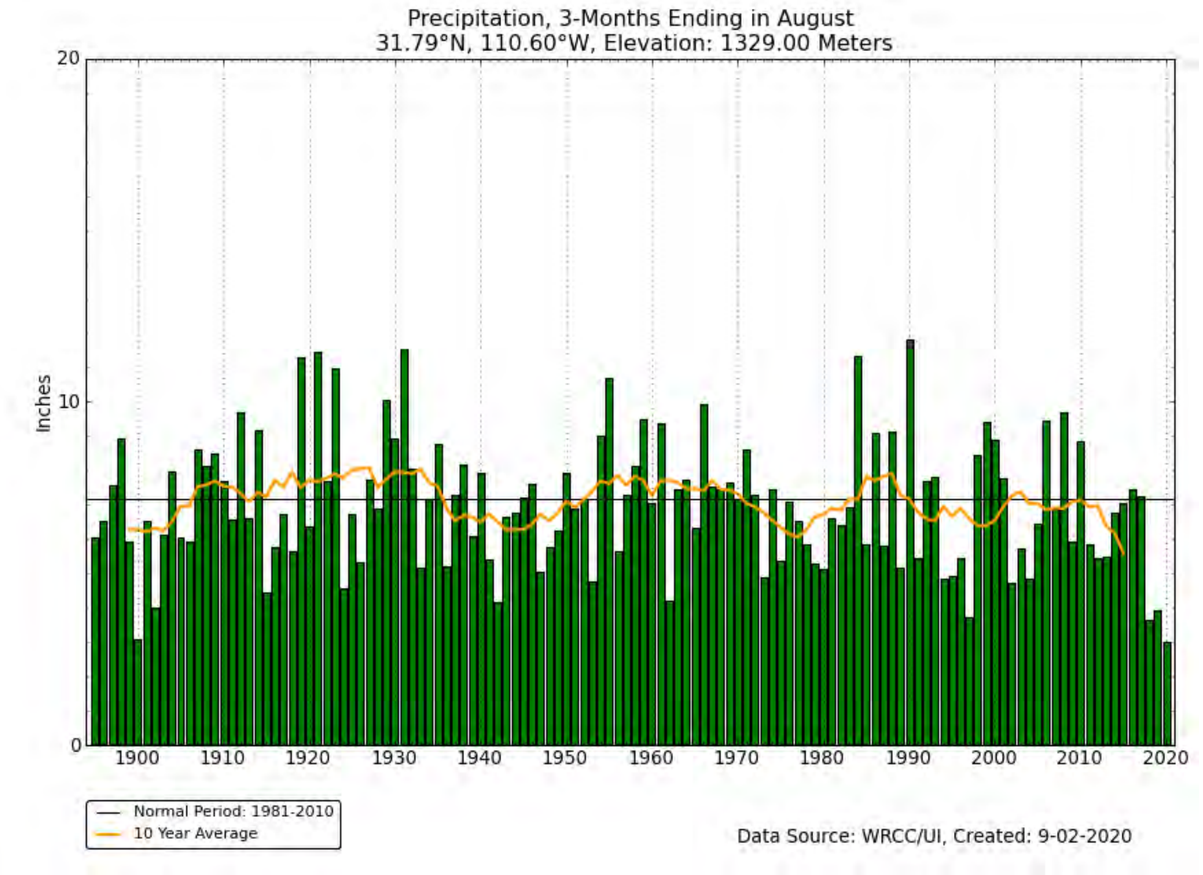

Winter

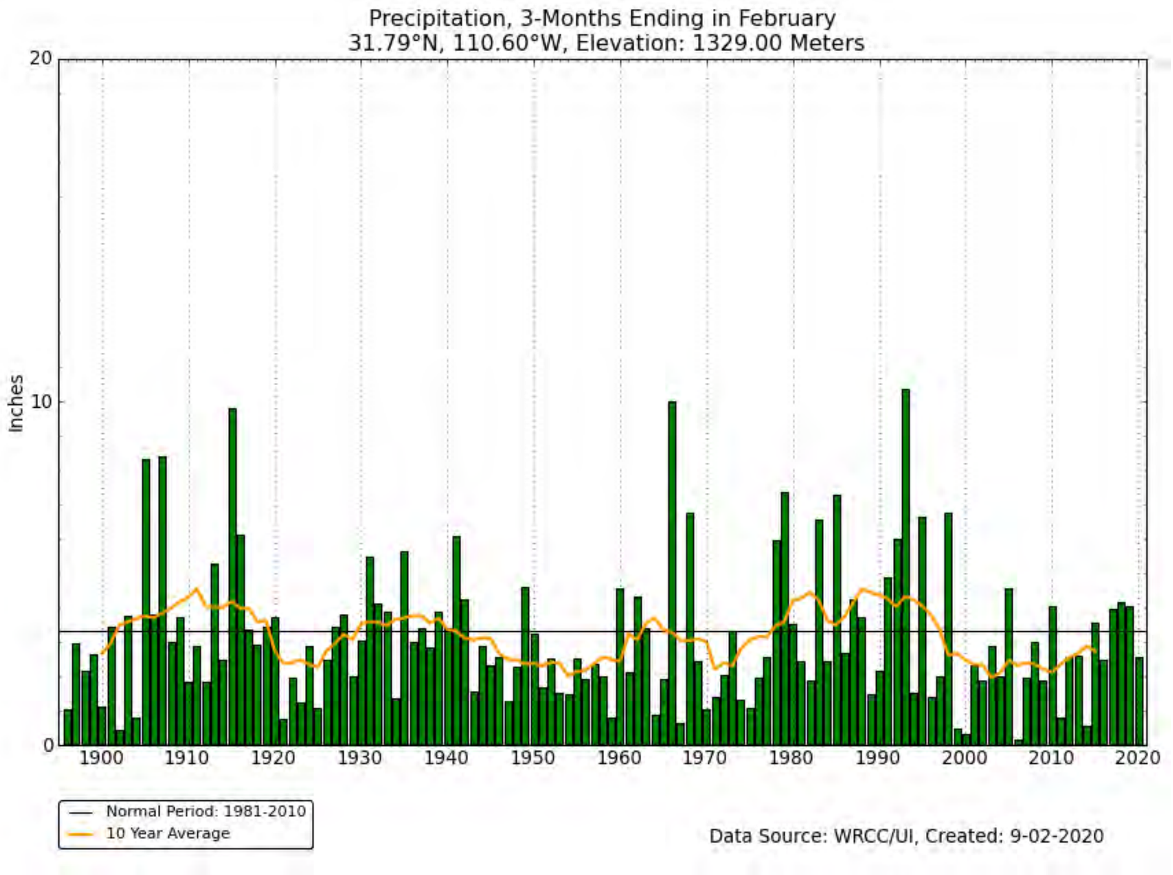

Source: http://www.wrcc.dri.edu/wwdt/
Assistance from: Mike Crimmins

Temperature

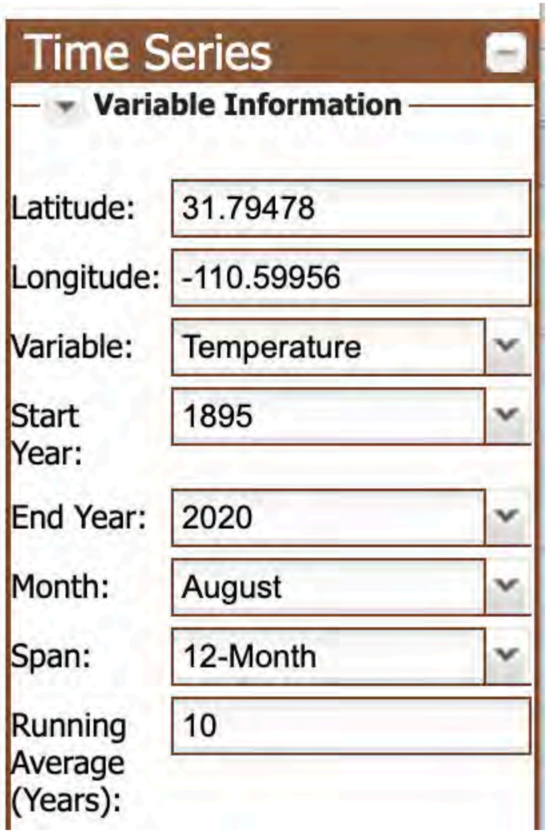

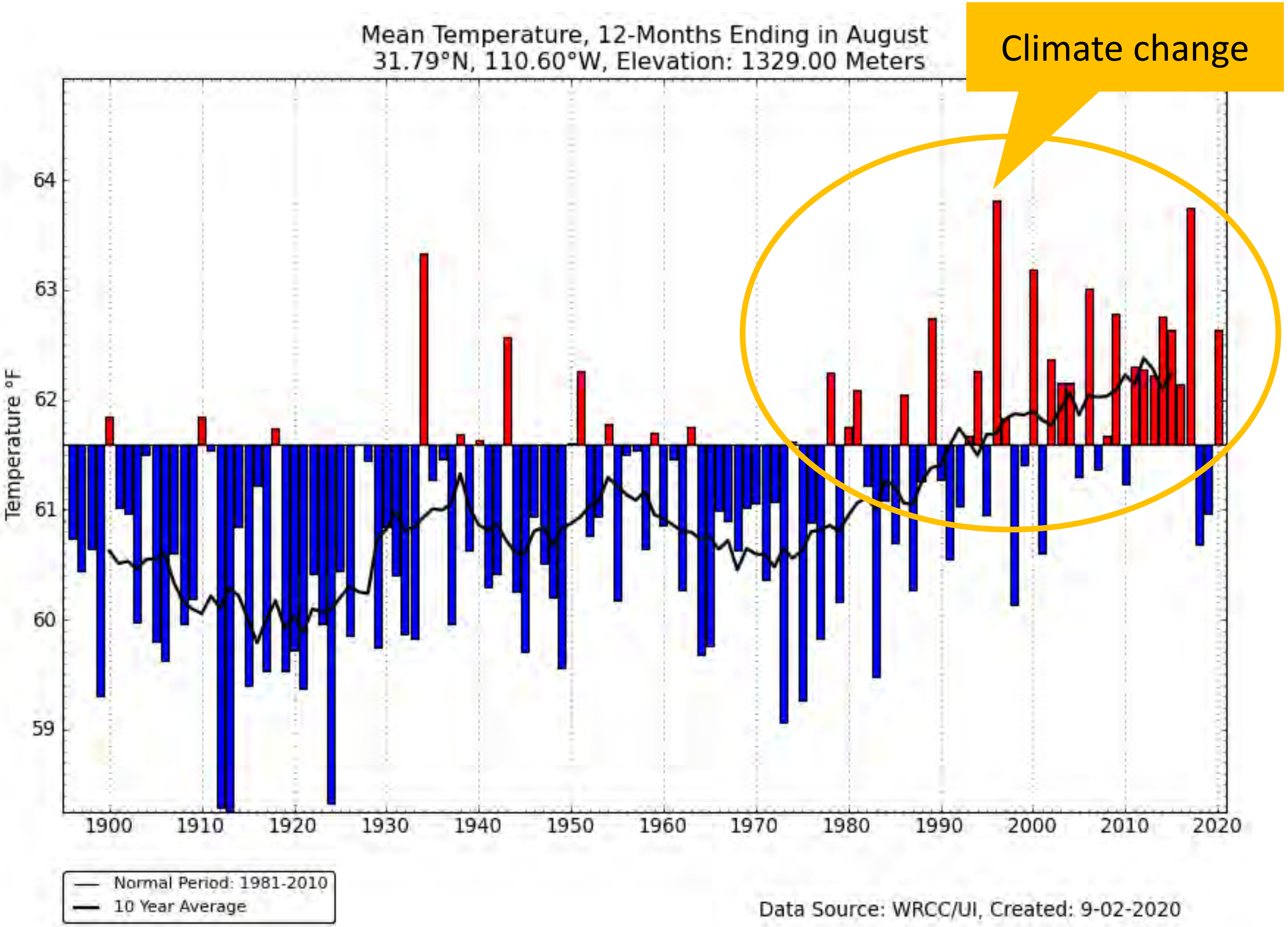

Source: http://www.wrcc.dri.edu/wwdt/
Assistance from: Mike Crimmins

Drought

- SPI – Standardized Index - the simples index for drought.
- Standard deviation of observed precipitation for a given point.

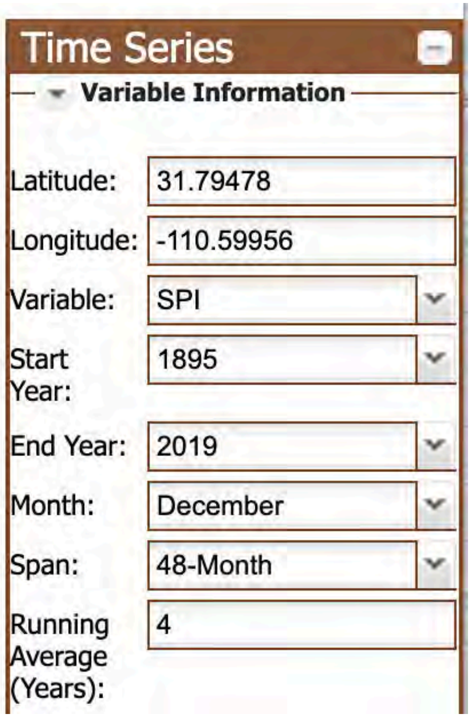

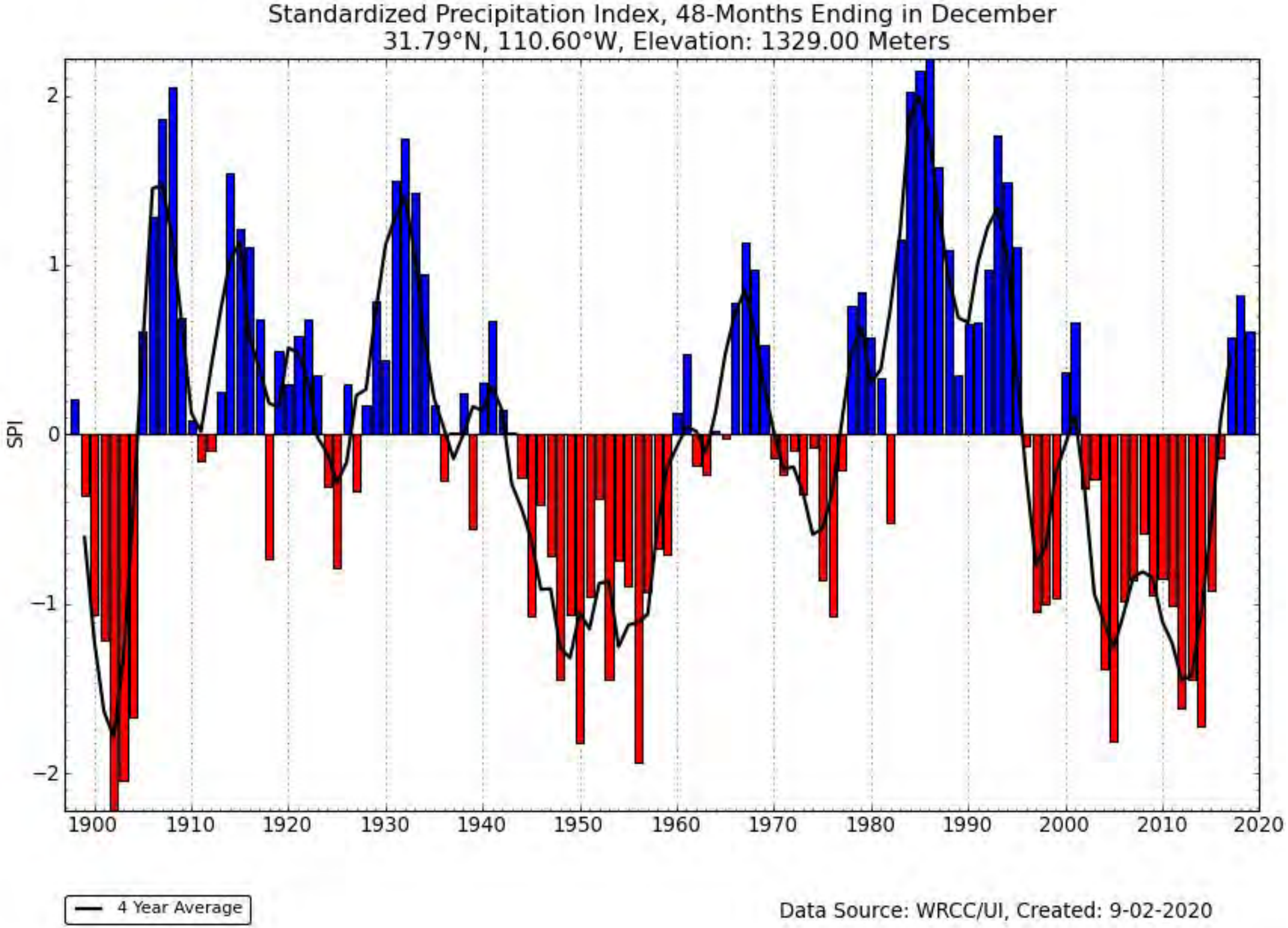

Source: http://www.wrcc.dri.edu/wwdt/
Help from: Mike Crimmins

Drought

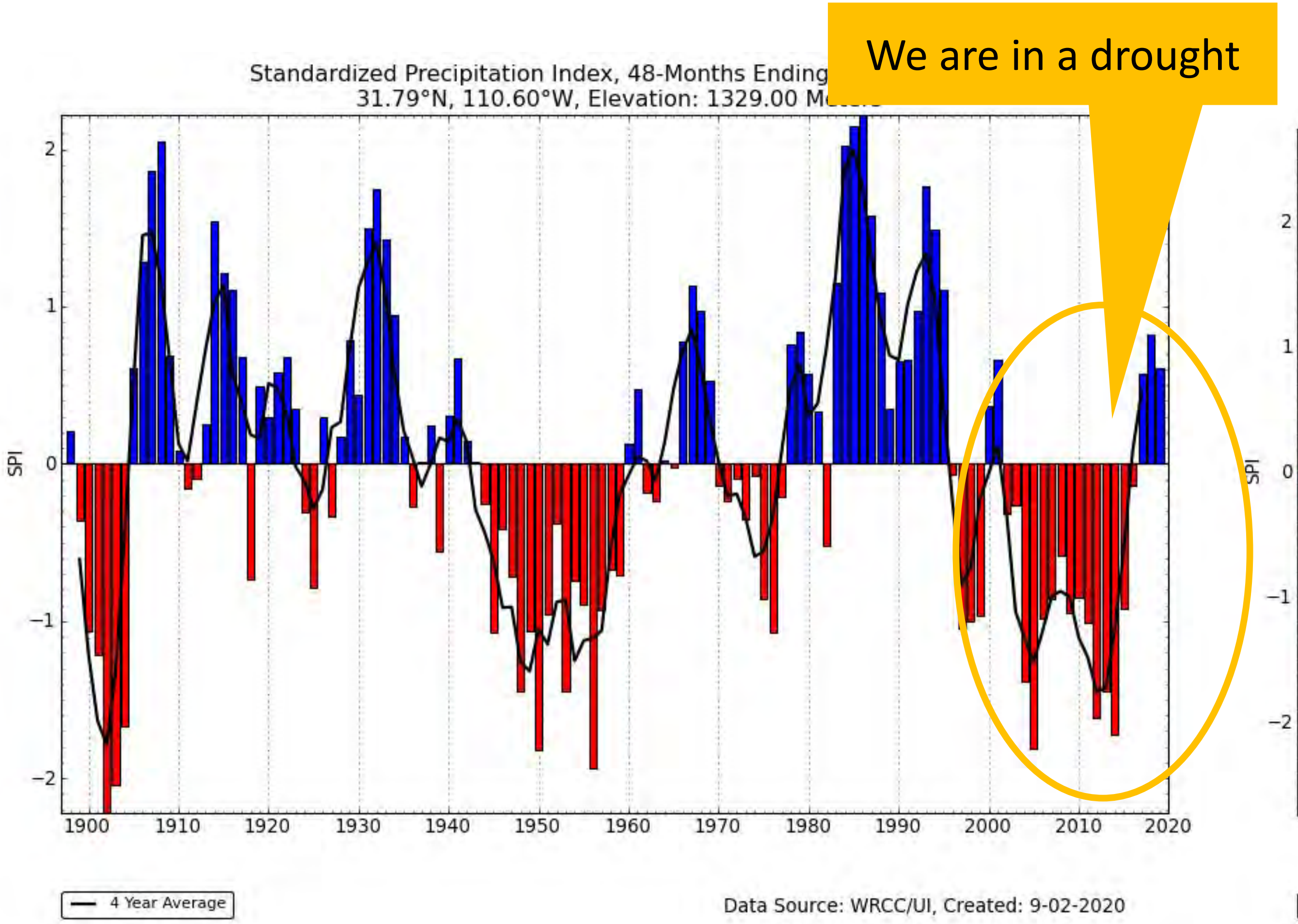

48 month SPI – 4 yr. average

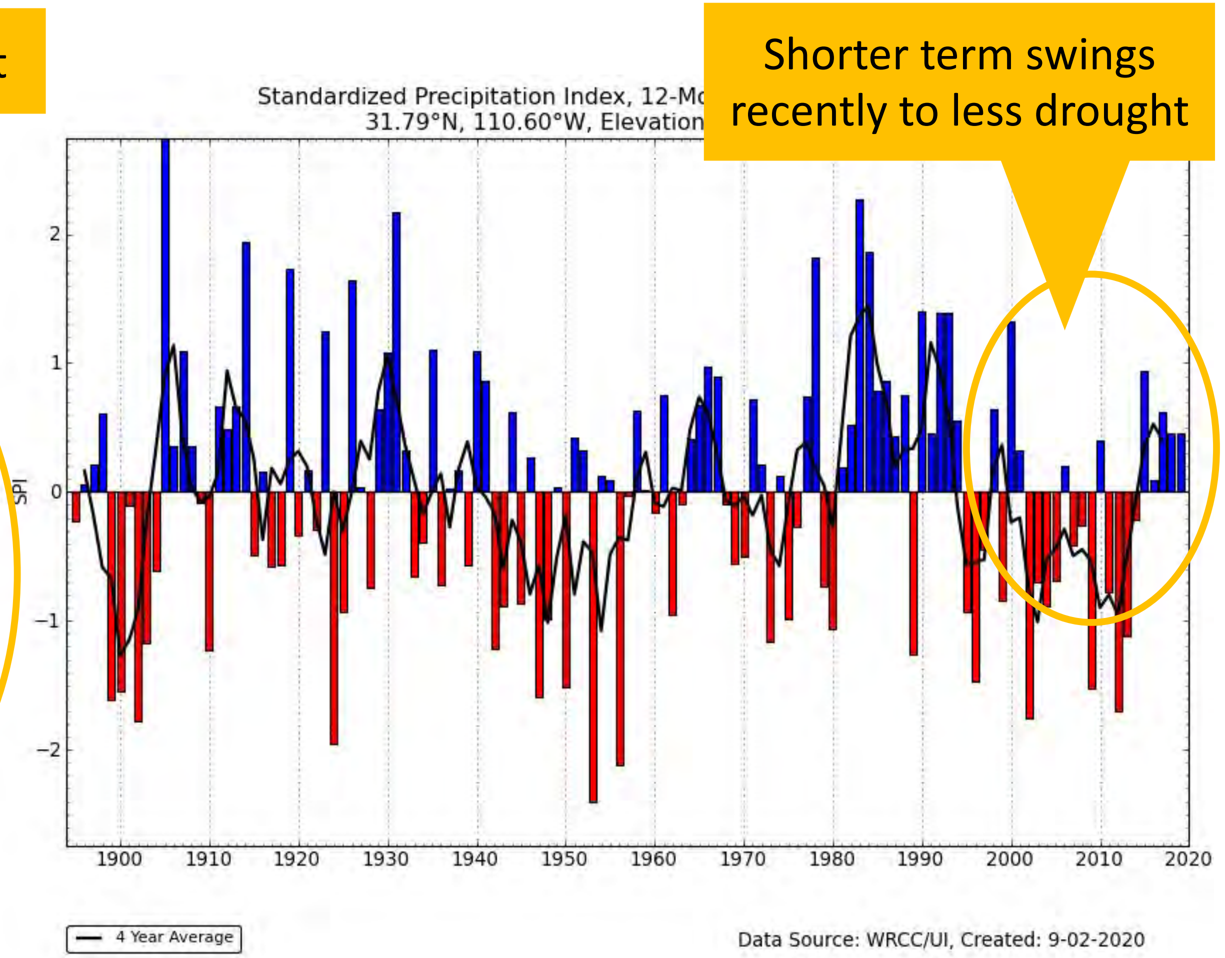

12 month SPI – 4 yr. average

Source: http://www.wrcc.dri.edu/wwdt/ Assistance from: Prof. Mike Crimmins

Climate - General trends

- **Precipitation:** Over the last 20 years we see a decline in both summer and winter precipitation. This is significant because winter precipitation is crucial for and the state of the health of the watershed.

- **Temperature:** Dramatic increase in temperature, particularly since 1980

- **Drought:** There have been periods of droughts since the early 1900s. We are in a drought since the mid 1990s, experiencing shorter term swings toward less drought.

Water

Indicator	No.	Description
**Groundwater levels**	4	Change from previous year in Jan. and June (highest/lowest)
	5	Wetlands
**Surface water quantity**	6	Wet-dry mapping (June – worst case)
	7	Gauges (Narrows and Pantano Dam)
	8	Monthly flows / base flows (average ft3/sec)/total flow
**Water quality**	9	TDS, dissolved oxygen PH, (fish)

Groundwater levels – Preserve (shallow wells)

Shallow Wells

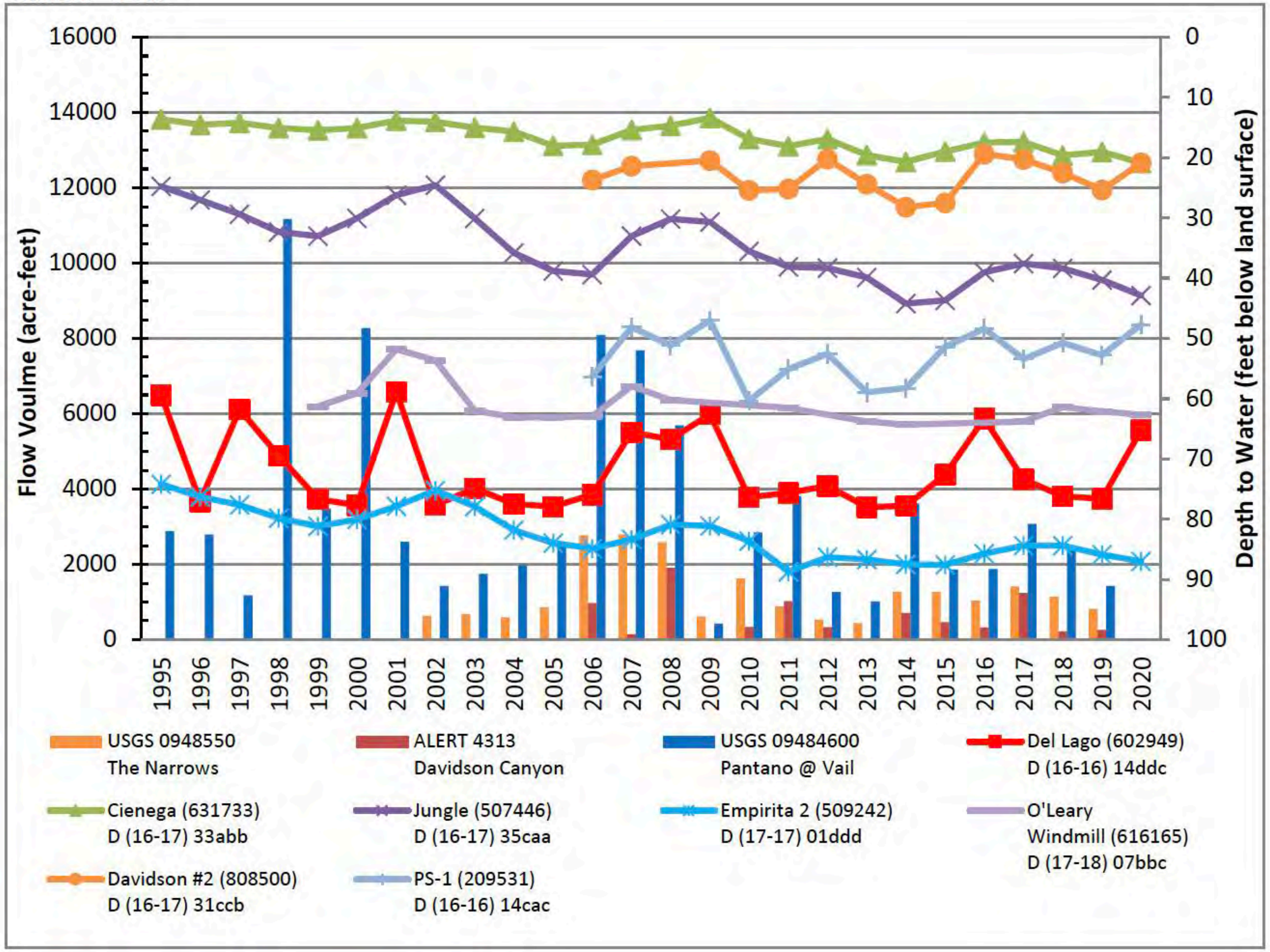

"On average, water levels declined 1.825 feet, with the largest decline of 2.6 feet observed at Pantano Jungle (Jungle Well). Water levels increased along Davidson Canyon by an average of 2.35 feet, with the largest occurring at Davidson 2 Well (4.5 feet)."

David Scalero (2020).

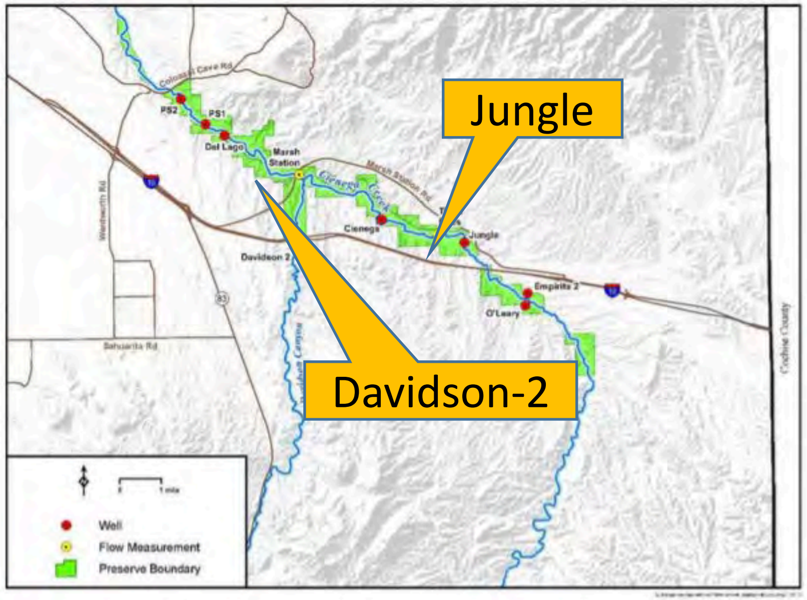

Source: David Scalero - Pima County Regional Flood Control District

Groundwater levels – Preserve (deep wells)

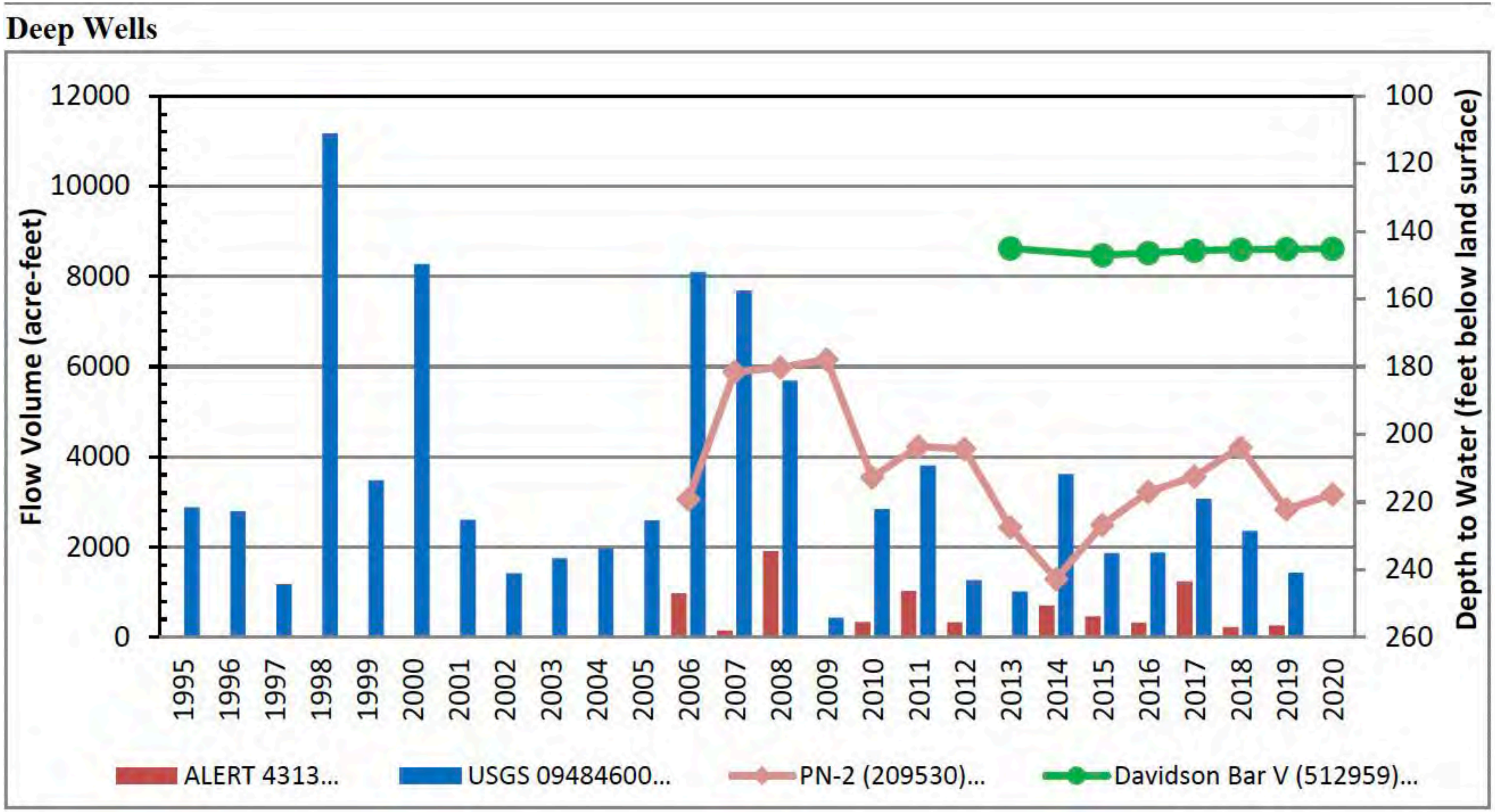

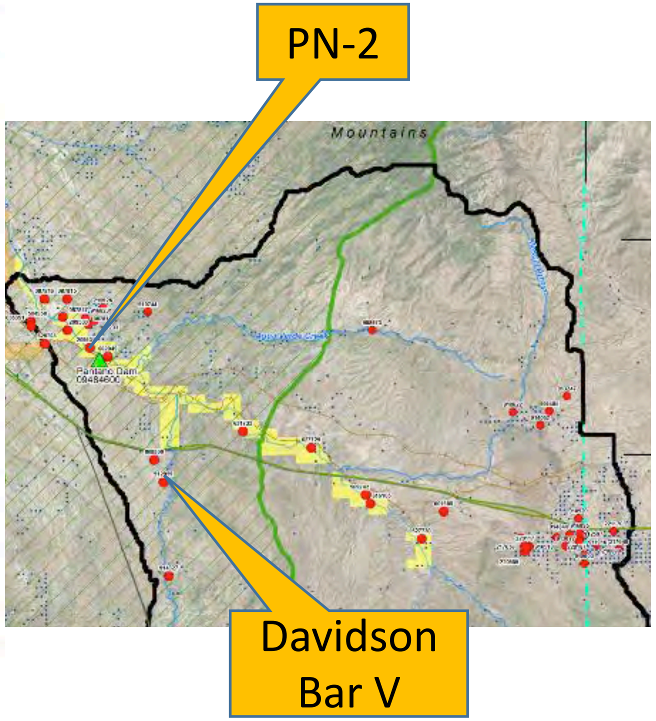

Source: Mead Mier - PAG and Frank Postillion - Pima County Regional Flood Control District

Groundwater levels – BLM (wells)

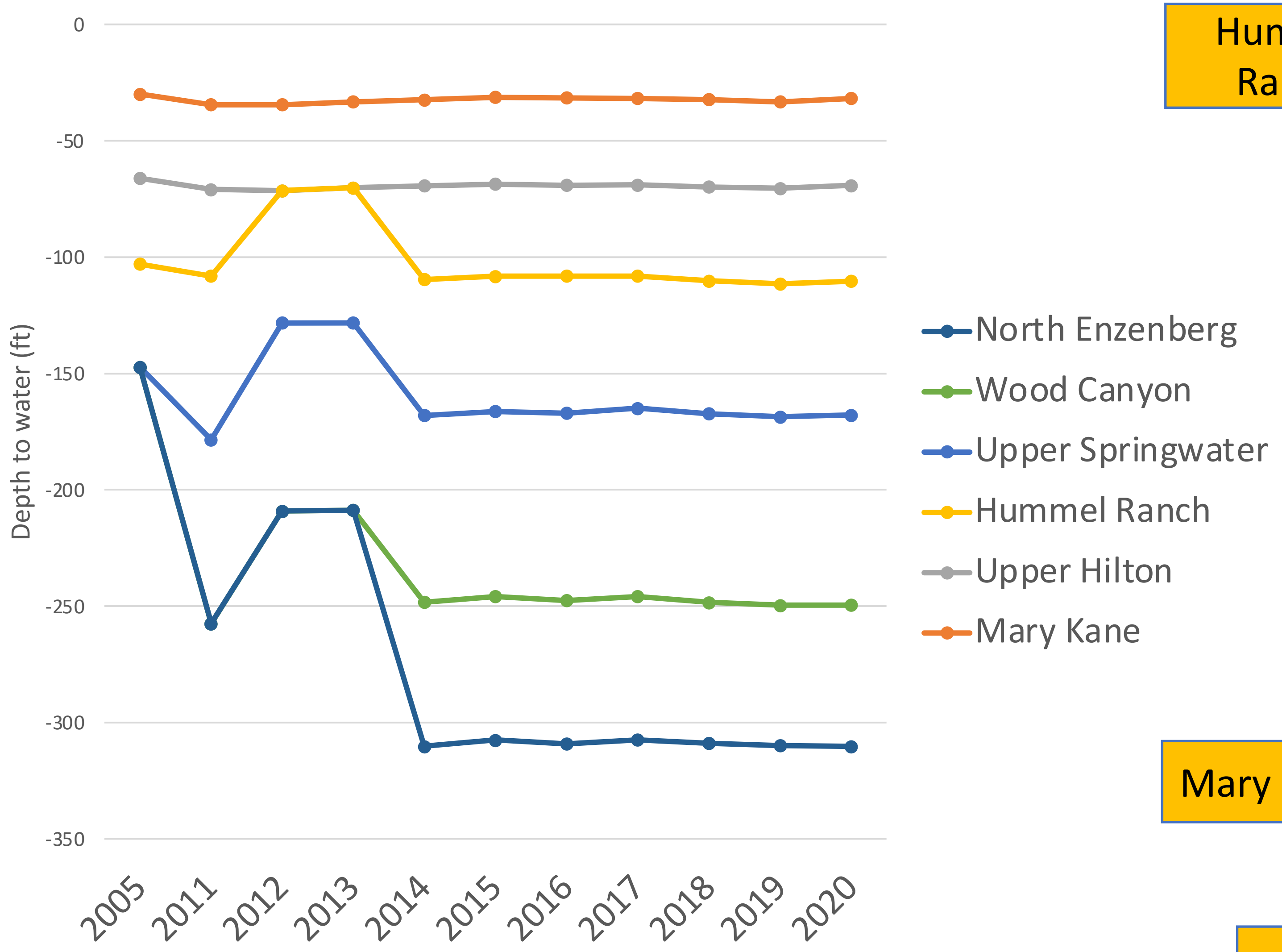

Source: Dave Murray, BLM

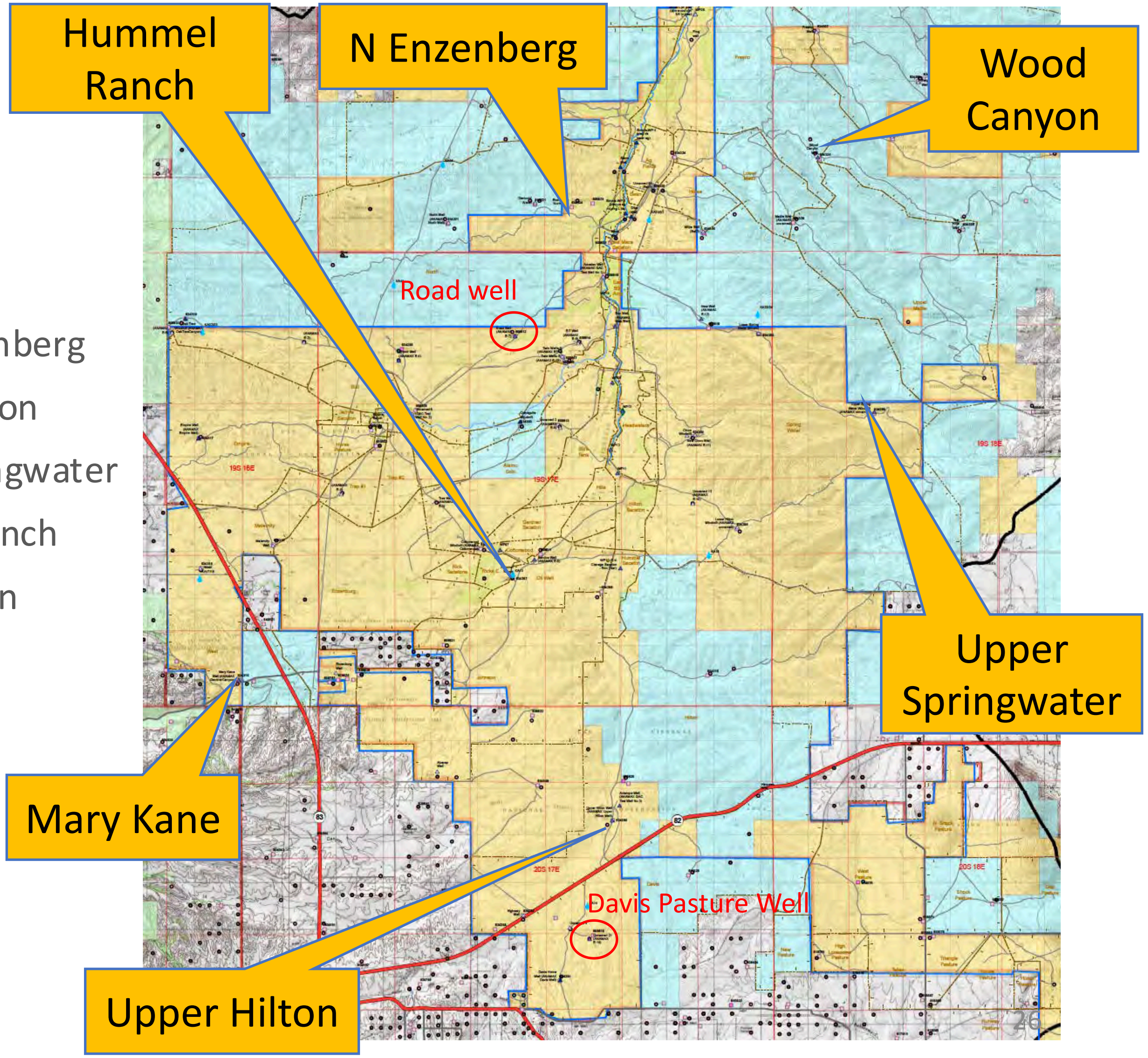

Wetlands

Spatial location and extent of wetlands from 2017

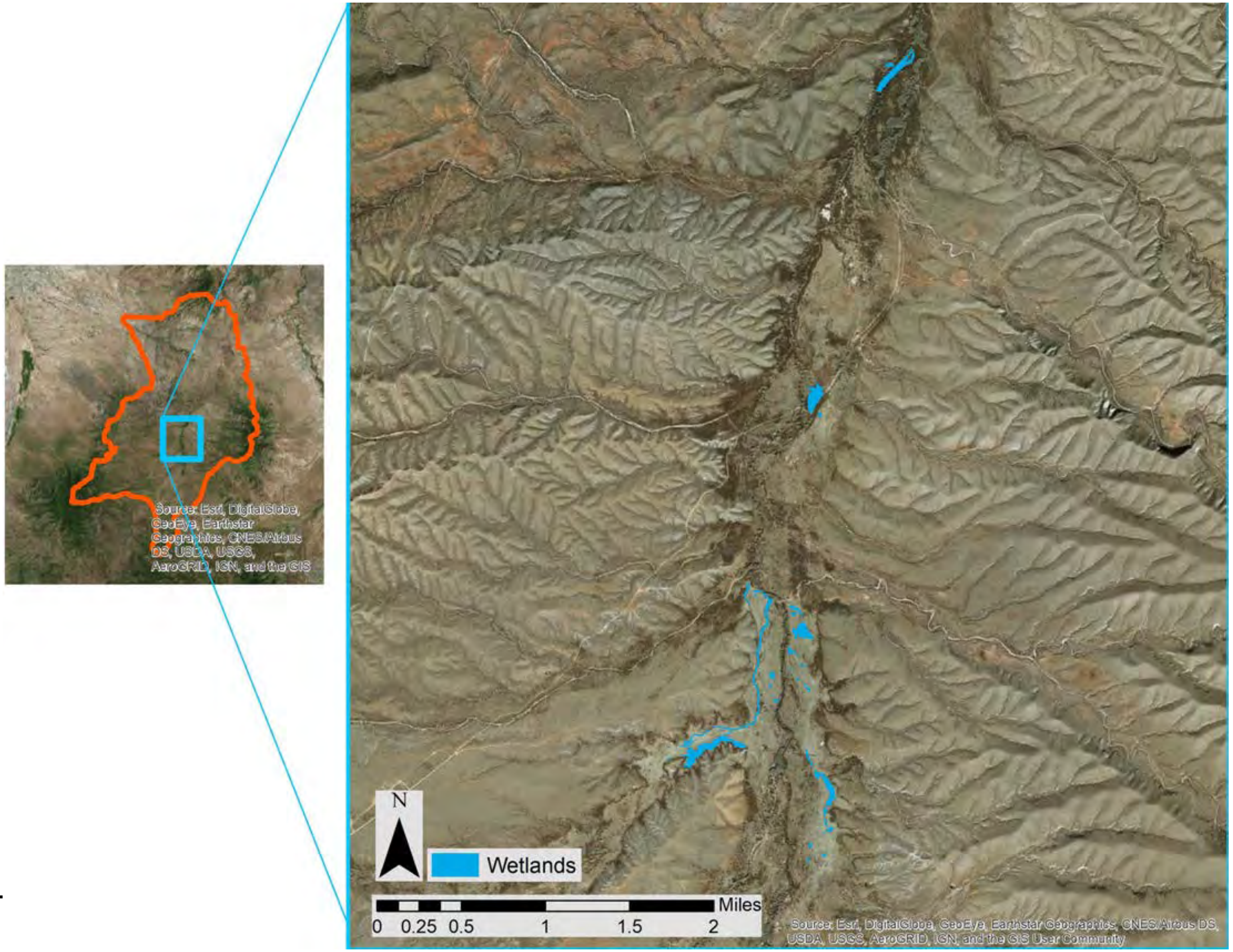

Source: Andrew Salywon and Ron Tiller - Desert Botanical Garden

Wetlands

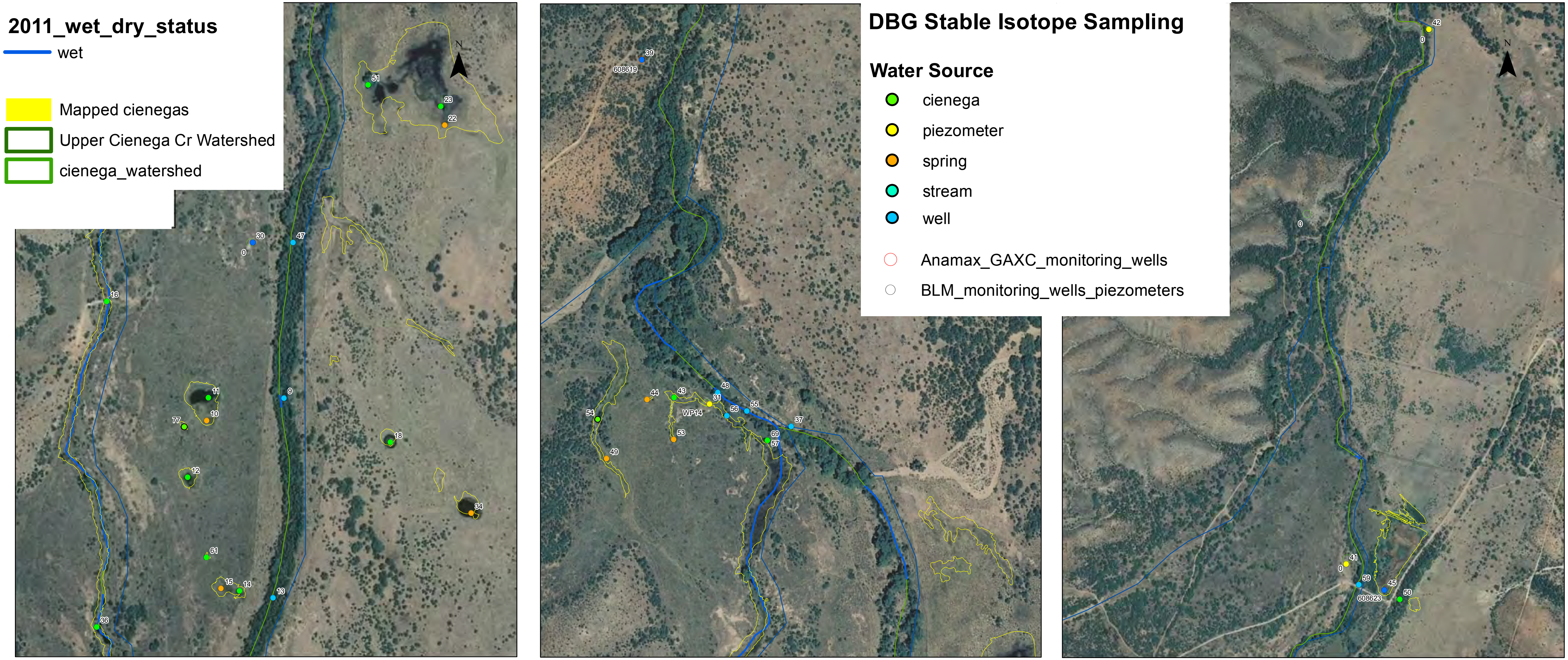

Source: Andrew Salywon and Ron Tiller – Desert Botanical Garden

Wetlands

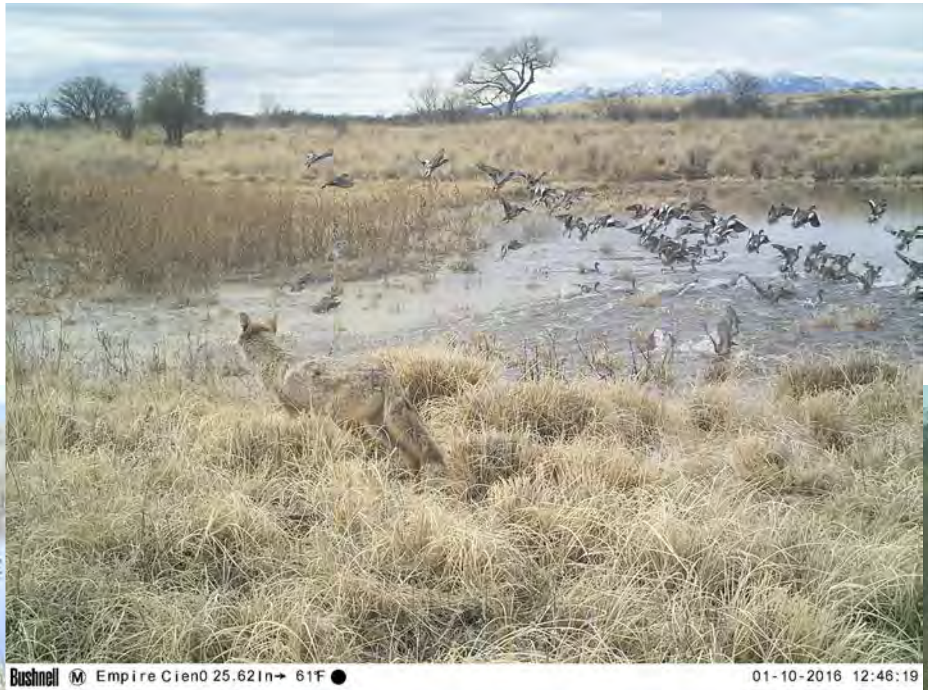

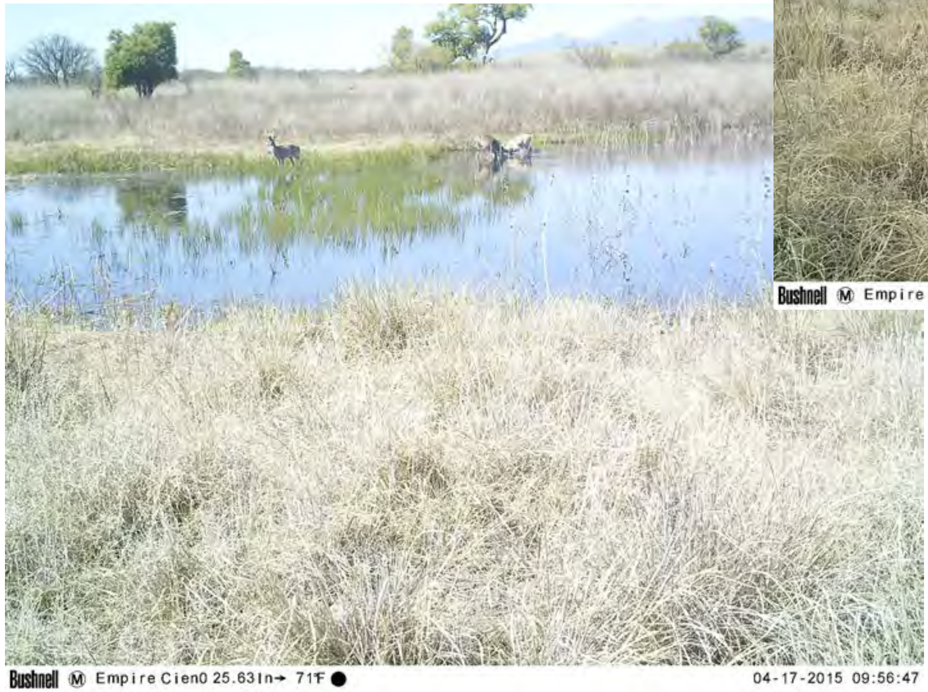

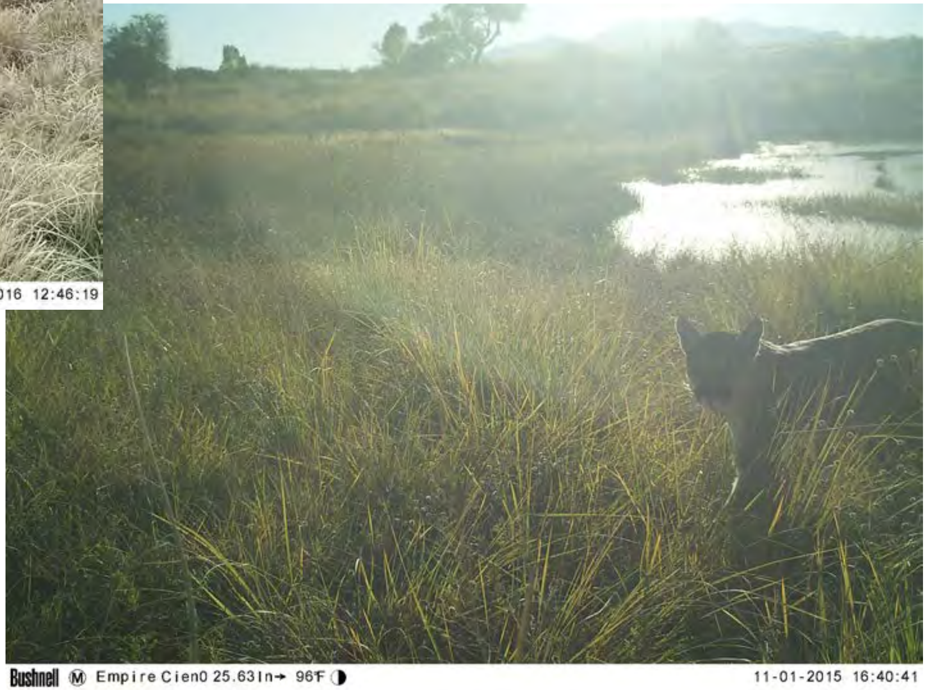

Source: Andrew Salywon and Ron Tiller – Desert Botanical Garden

Wet-Dry

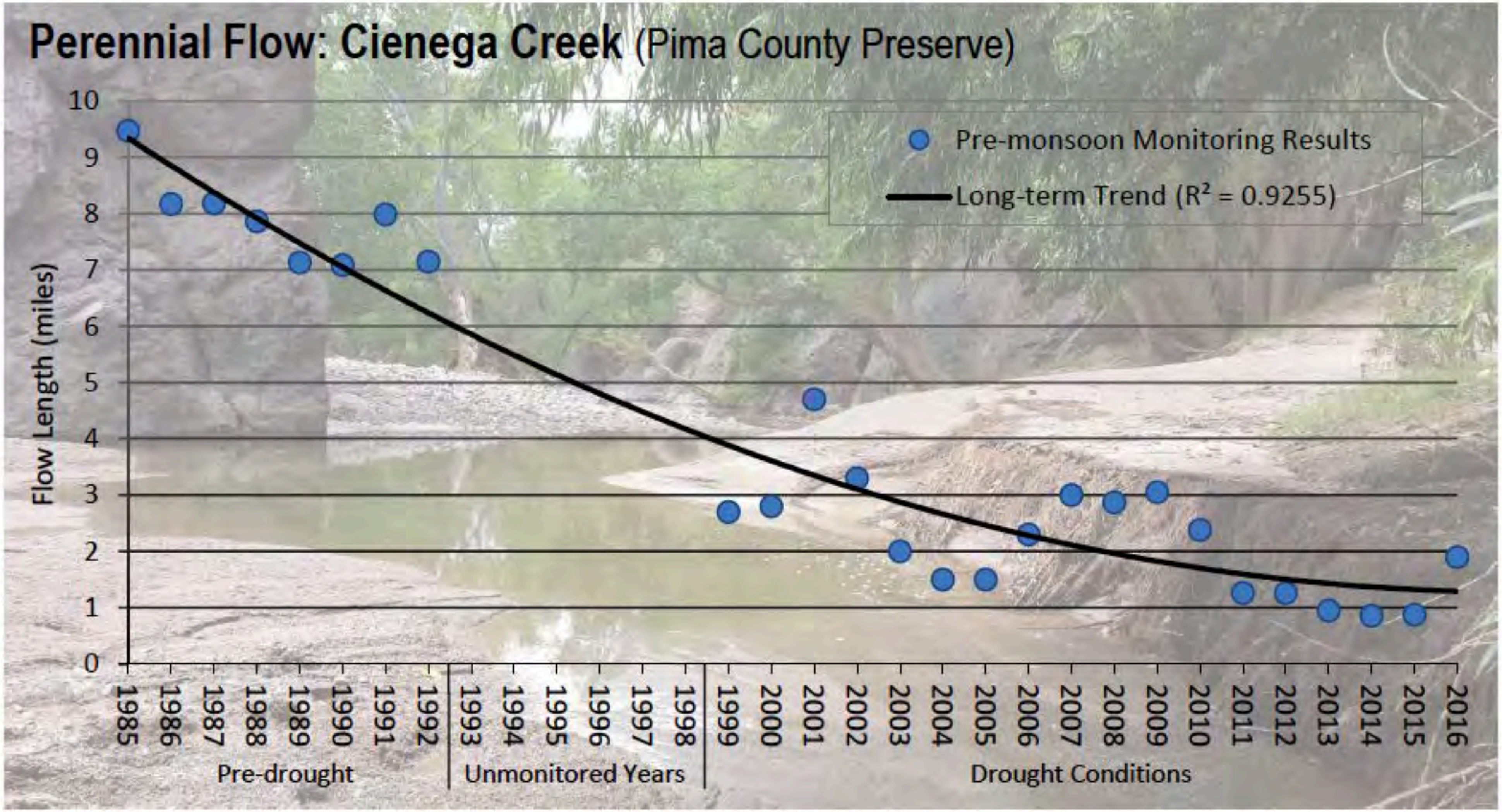

Source: Mead Mier, PAG

Wet-Dry

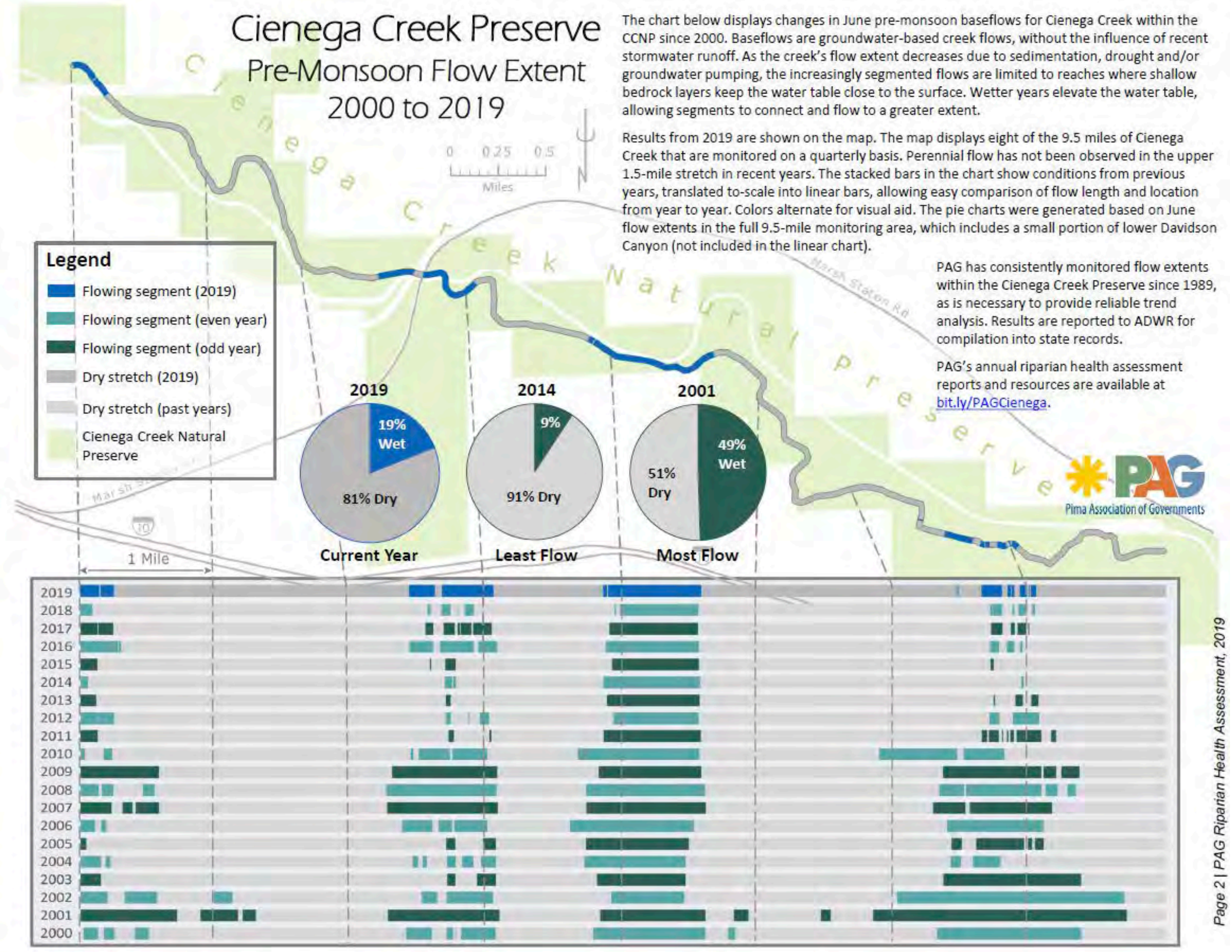

Source: Mead Mier, PAG

Wet-Dry

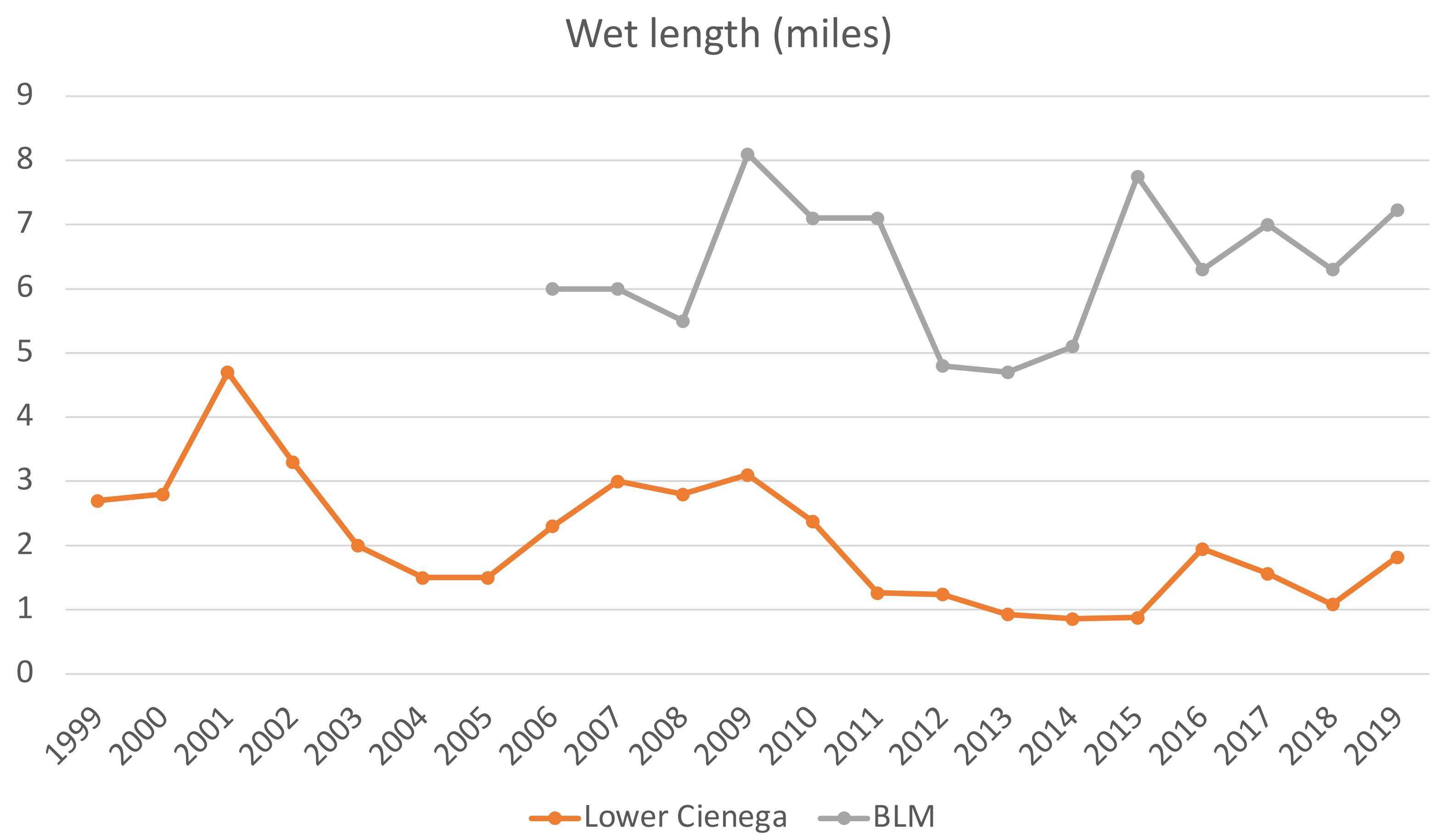

Source: Ian Murray, Pima County; and Dave Murray, BLM

Gauges

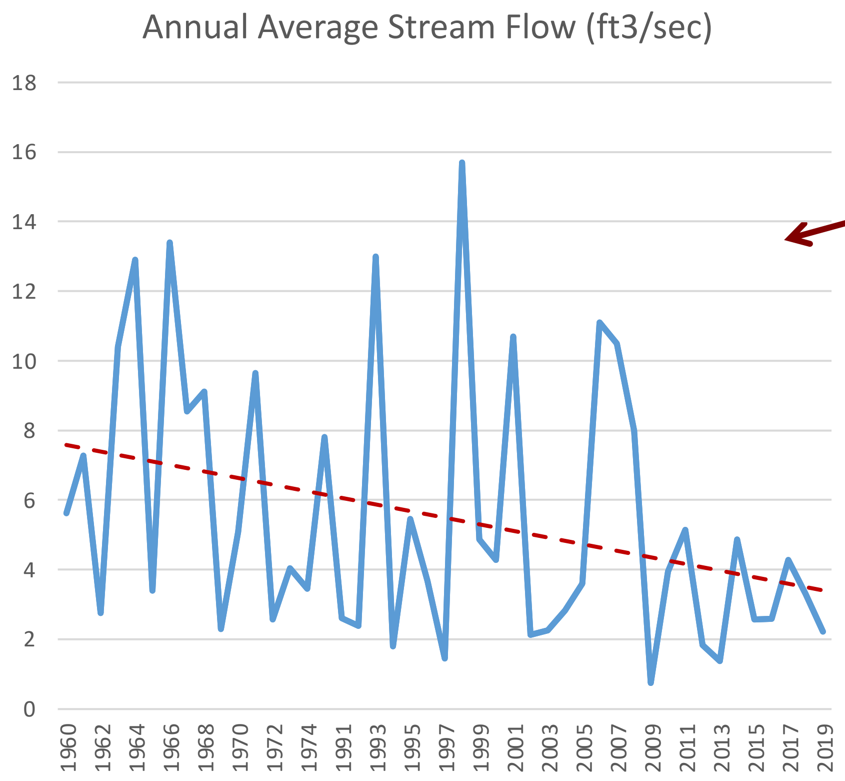

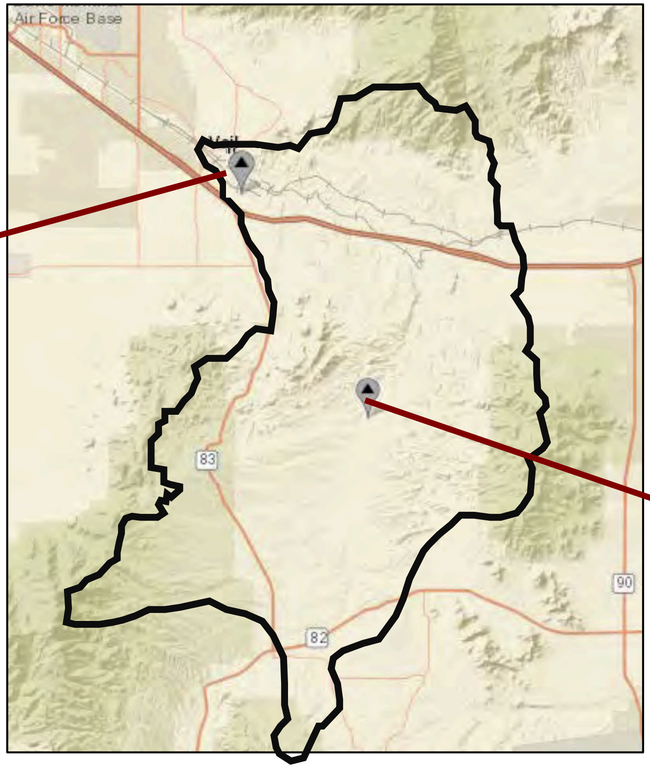

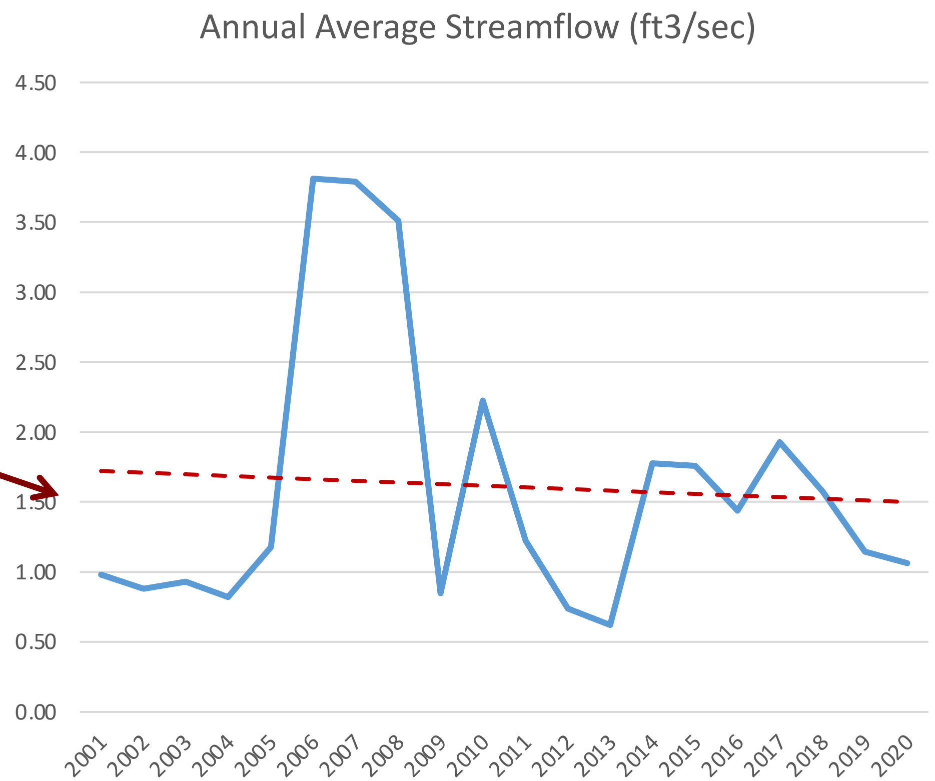

Pantano Wash
Gauge # 09484600

Cienega Creek
Gauge # 09484550

No trend

Source: waterdata.usgs.gov

Stream Flows – Cienega Creek Natural Preserve

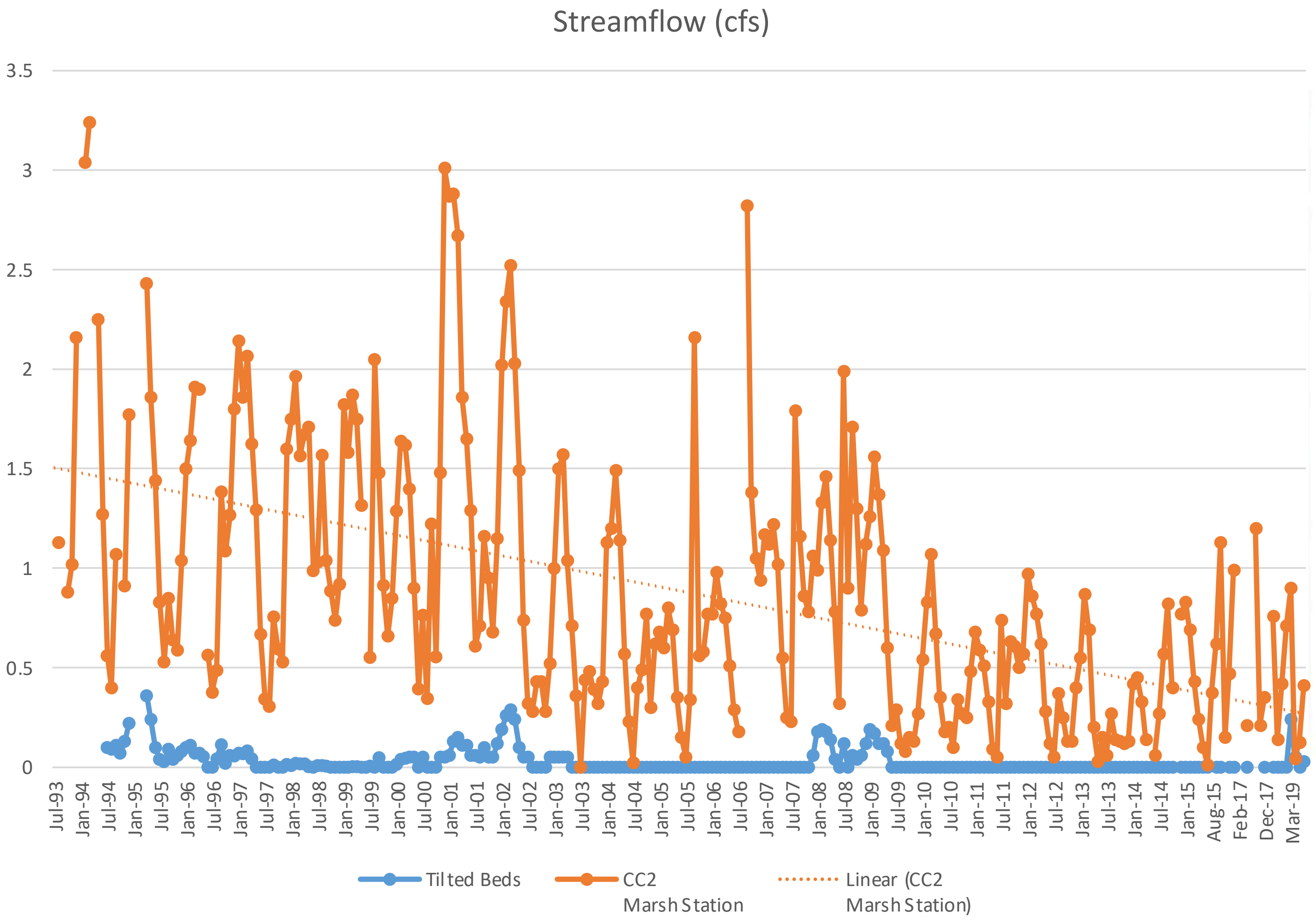

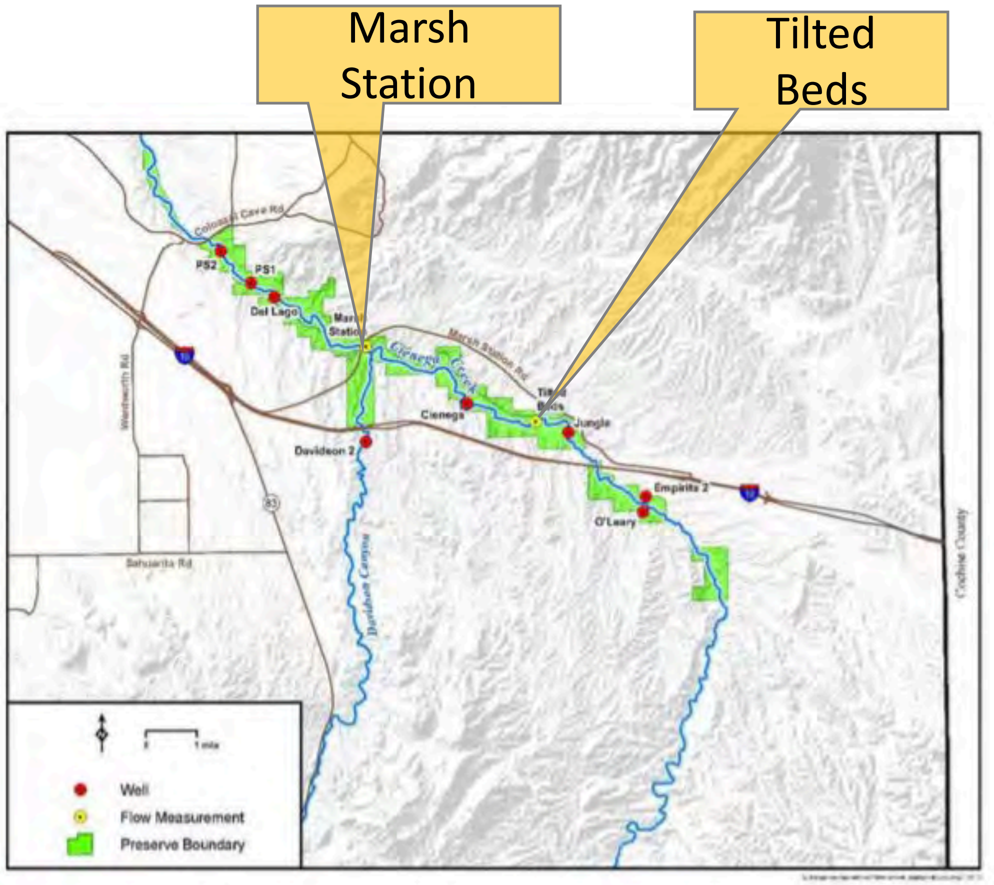

Source: David Scalero, PAG; Pima County Regional Flood Control District

Stream Flows - BLM

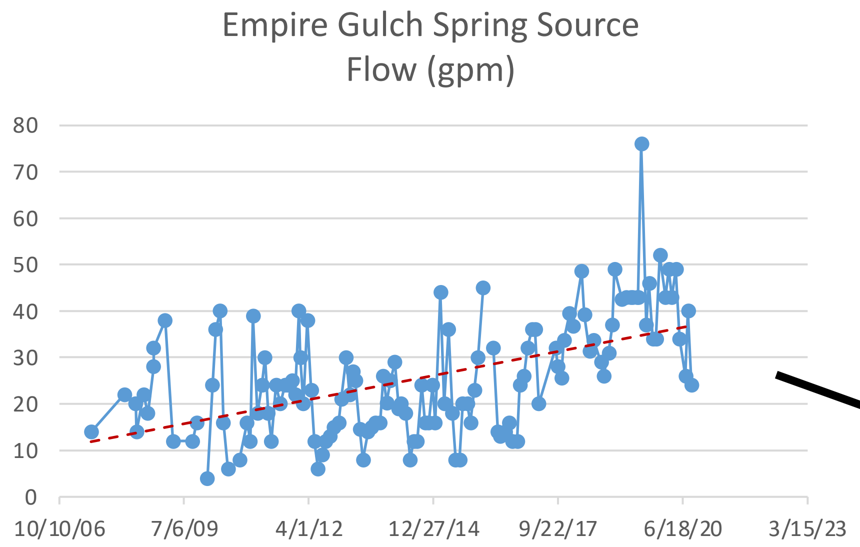

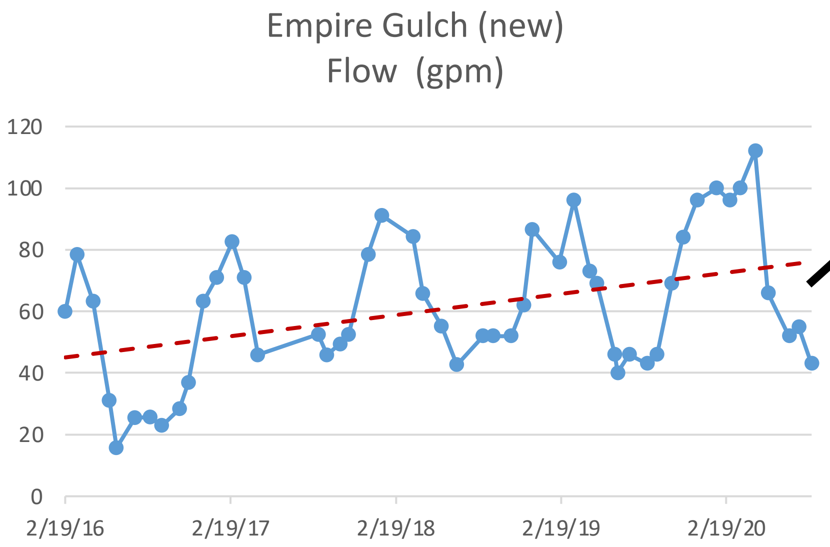

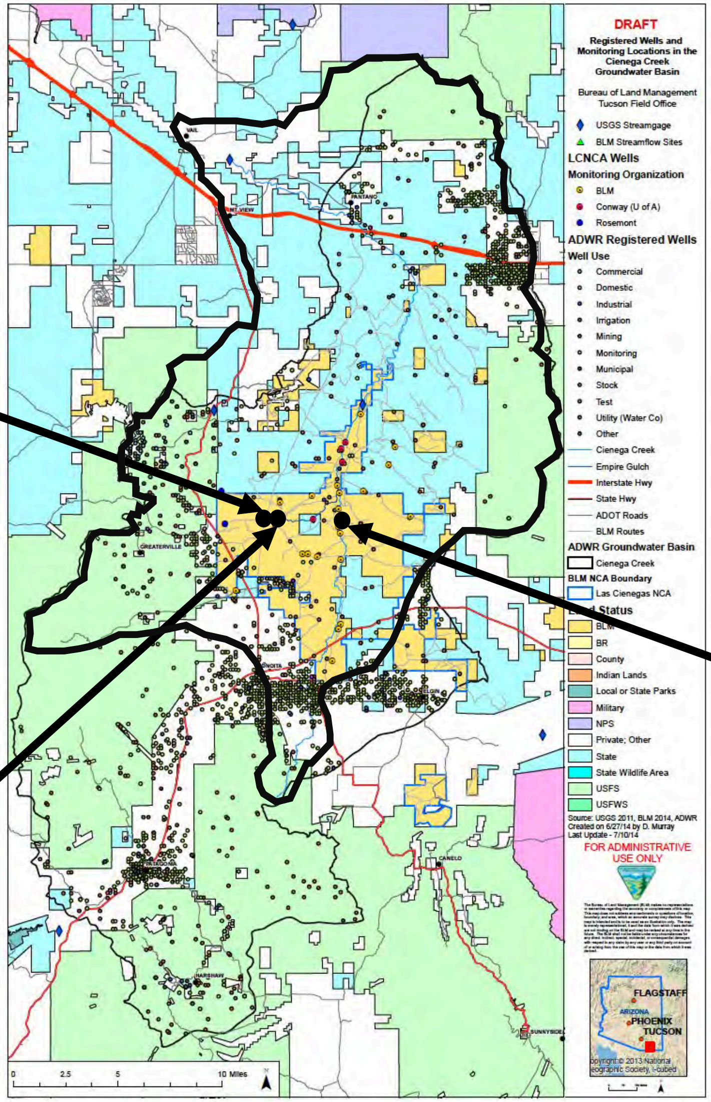

- Stream flow is measured once a month, and varies depending on which day and hour the data is collected.
- This data provides a ‘snapshot in time.’

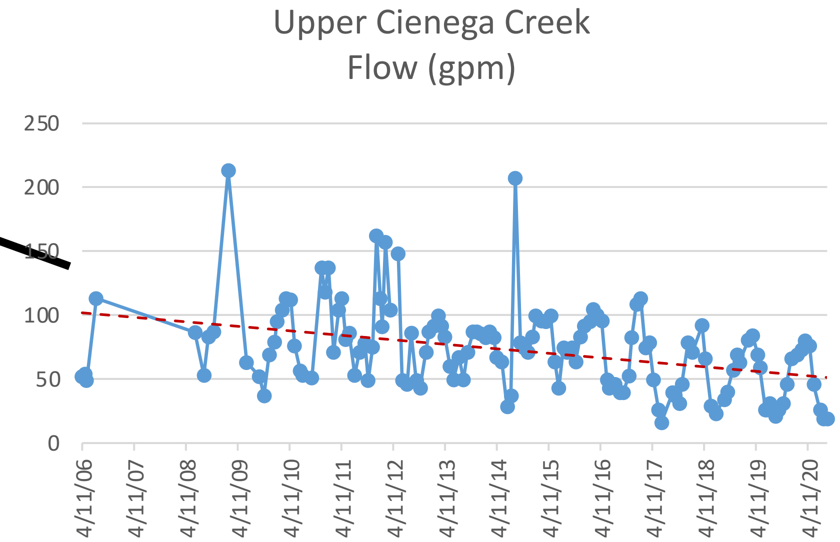

Source: Dave Murray and Peggy Monkemeier, BLM

Water quality – Pima County

PH - Cienega 2

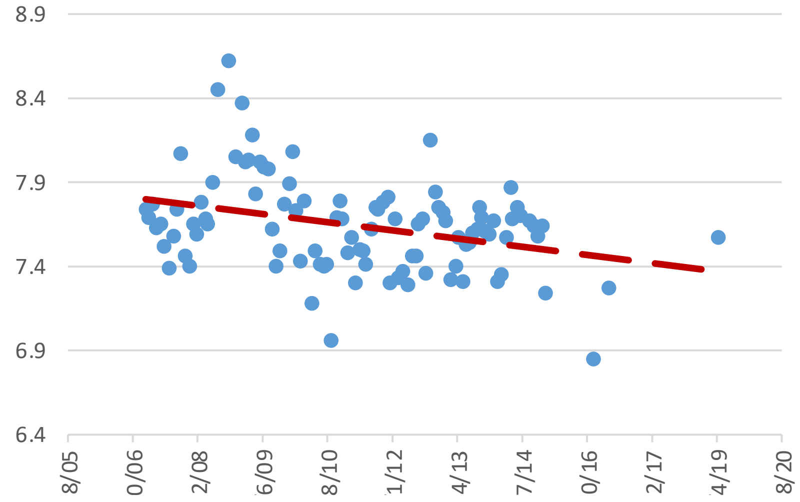

PH - Davidson 1/3

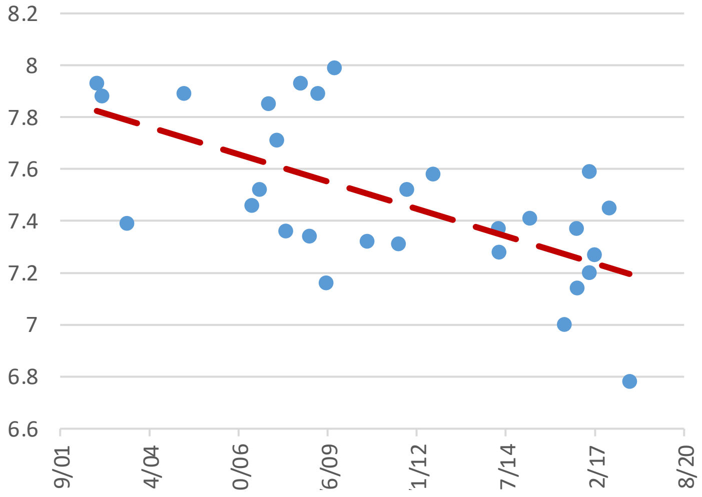

Temp. (F) in Cienega 2

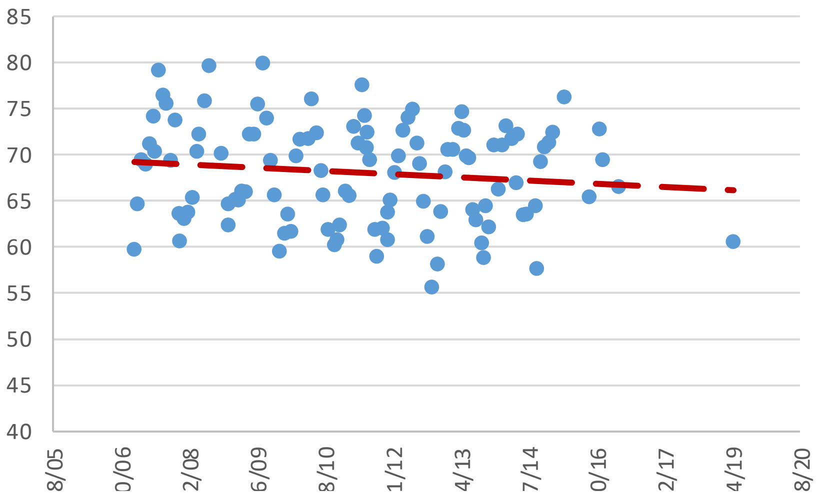

Temp. (F) Davidson 1/3

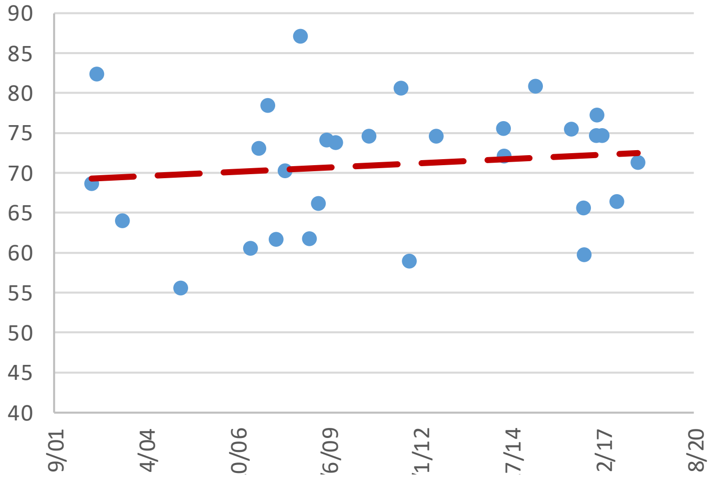

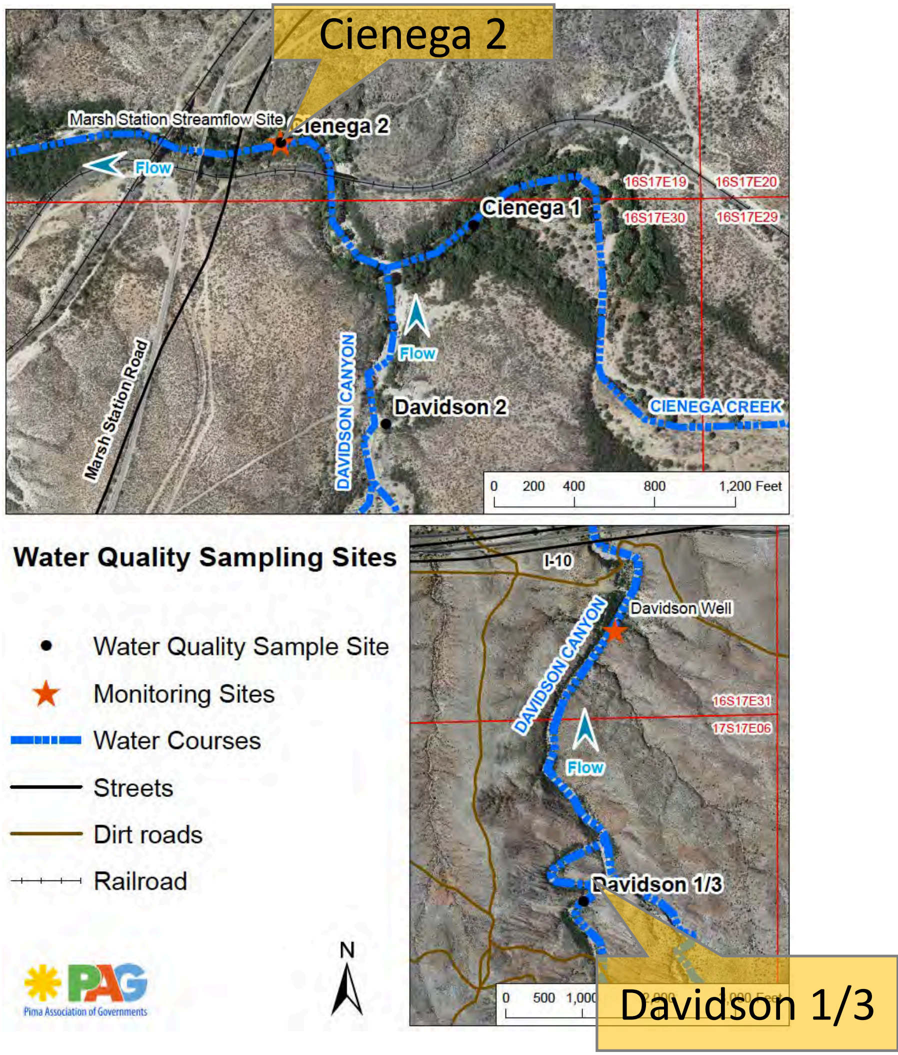

Source: Mead Mier, PAG and David Scalero, PC Flood Control District

Water quality – Pima County

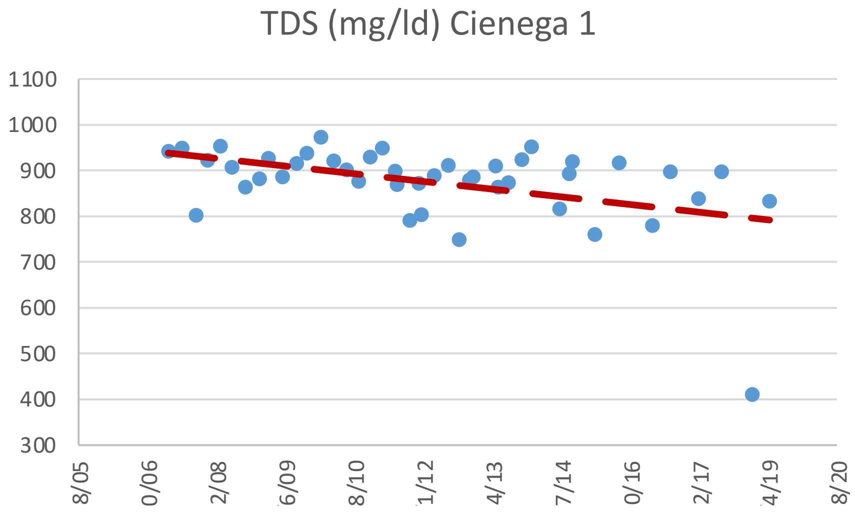

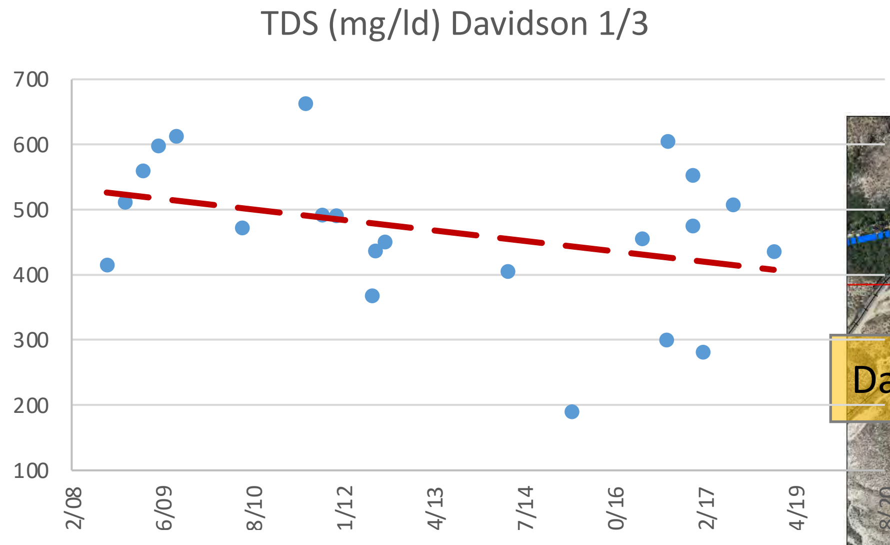

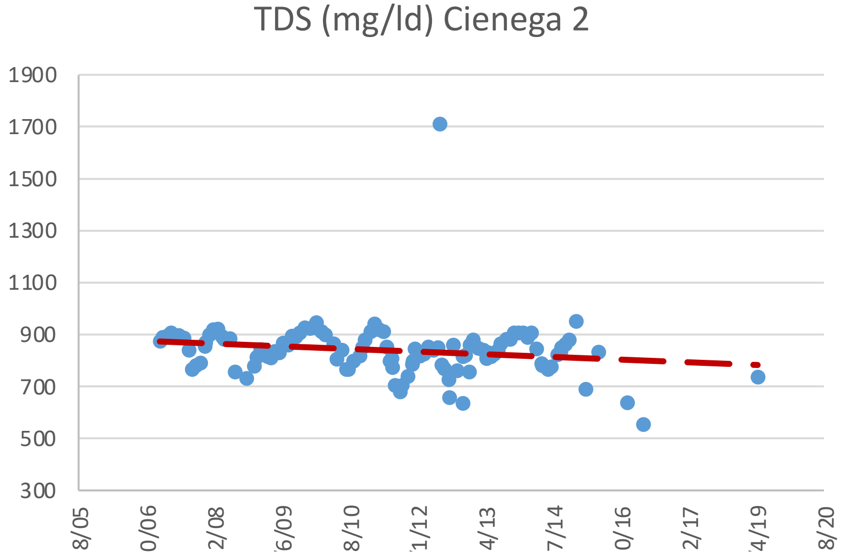

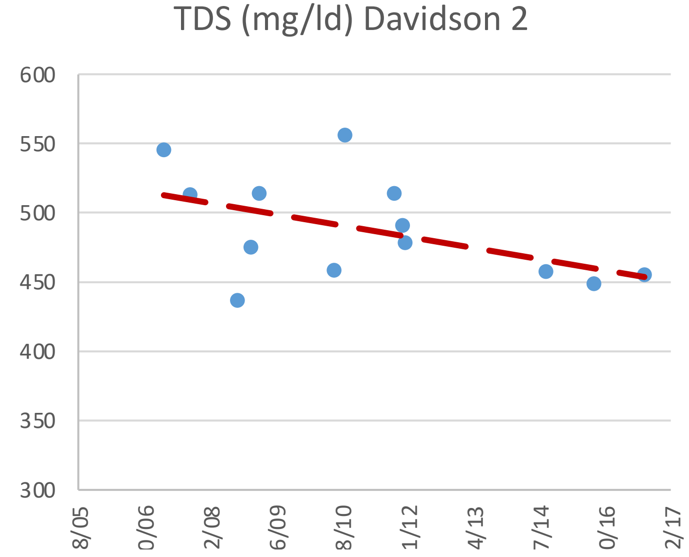

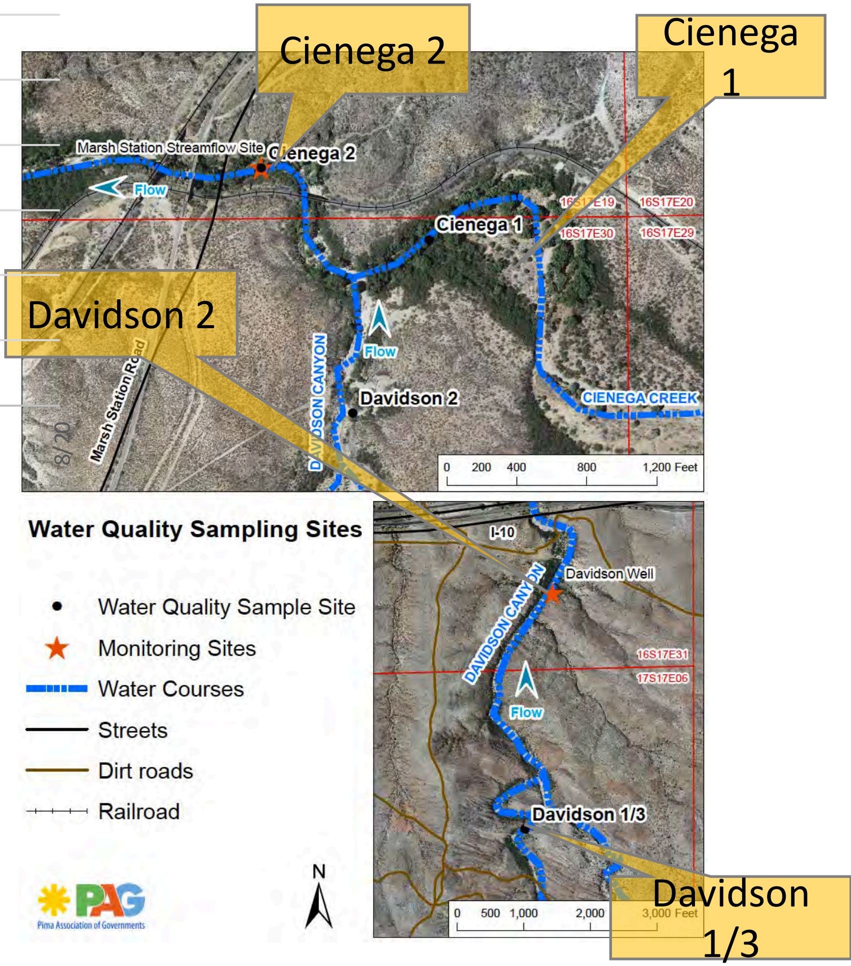

Source: Mead Mier, PAG and David Scalero, PC Flood Control District

Water quality – Pima County

Figure 11. Cienega Watershed Conductivity (2002 - 2015)

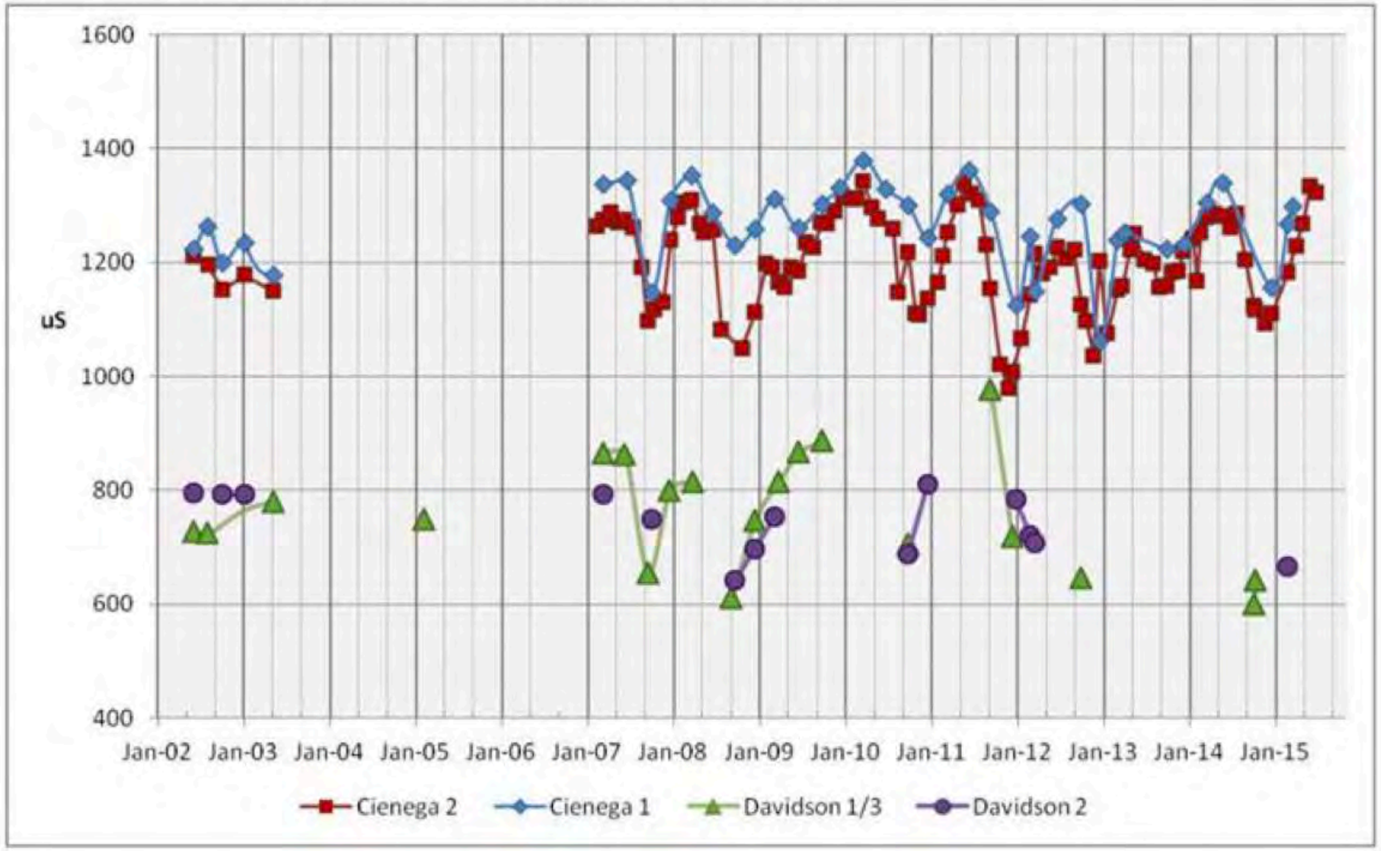

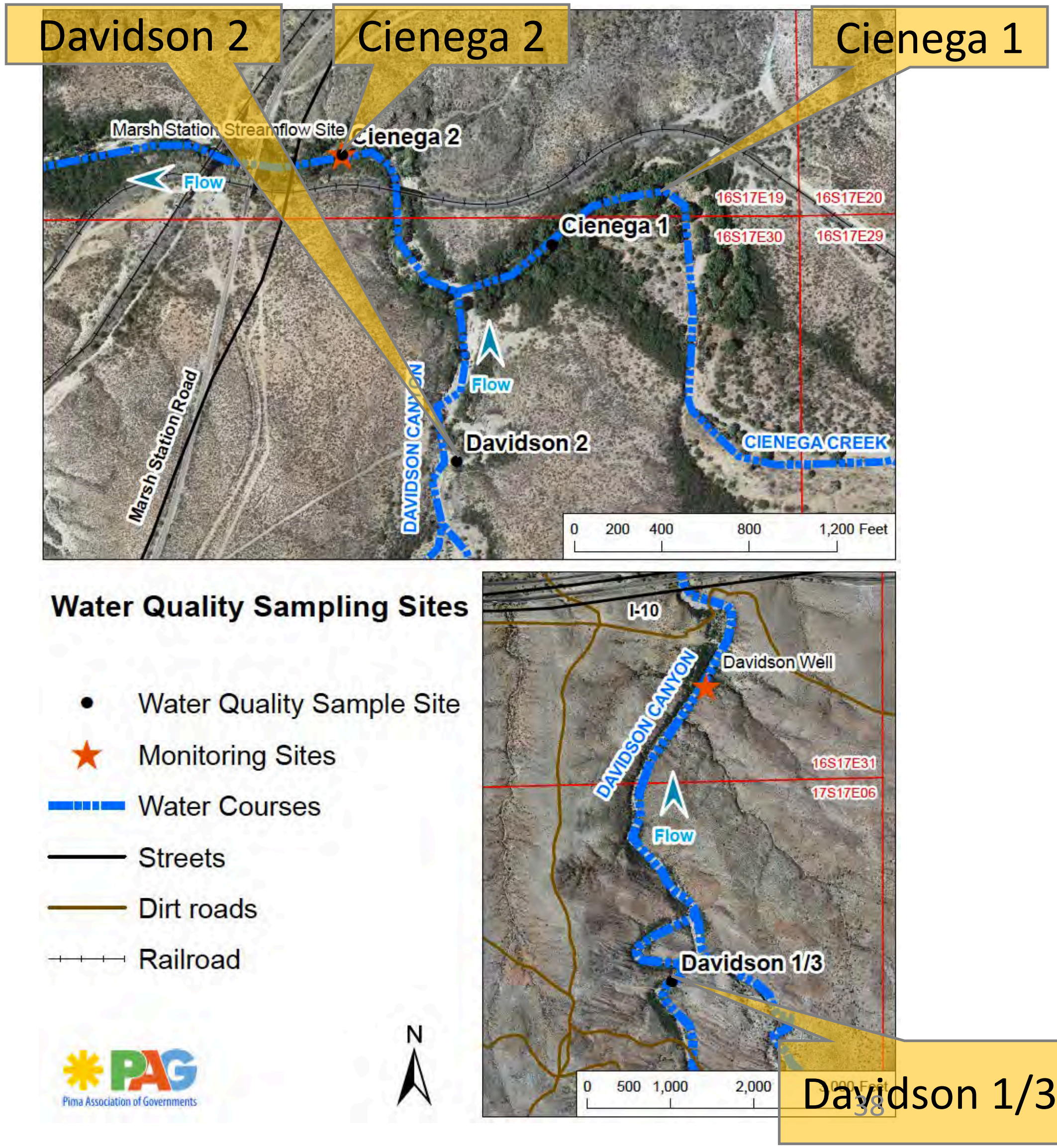

Source: Mead Mier, PAG

Water quality – Pima County

Figure 14. Cienega Watershed – Comparison of Average Conductivity of 2002-2003 to 2007-2011 Time Periods per Site

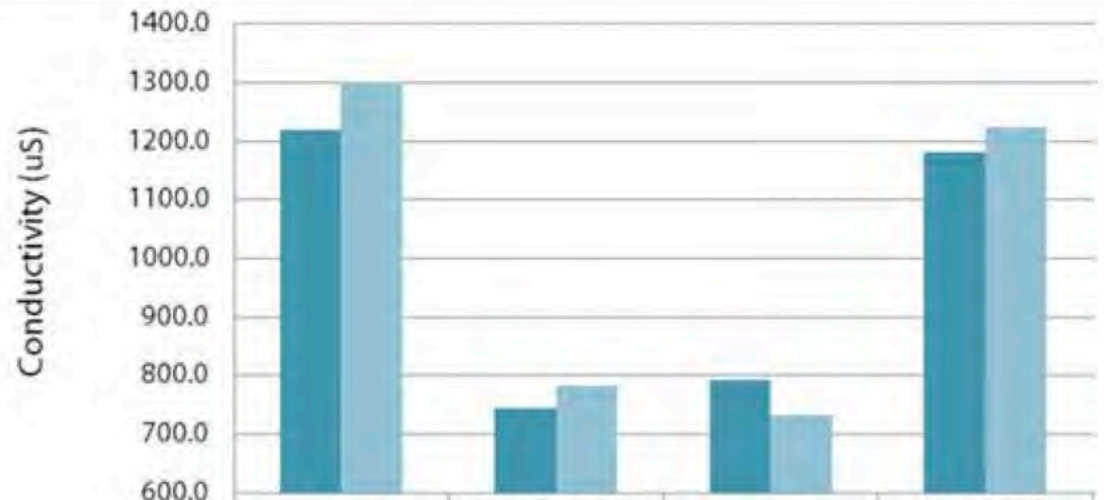

	Cienega 1 (Upstream of Confulence)	Davidson 1/3 (South/ Upstream of I-10)	Davidson 2 (Close to Confluence)	Cienega 2 (Marsh Station, Downstream)
Average Conductivity (uS) 2002-2003	1219.4	744.4	792.8	1180.7
Average Conductivity (uS) 2007-2011	1300.3	783.7	732.5	1225.7

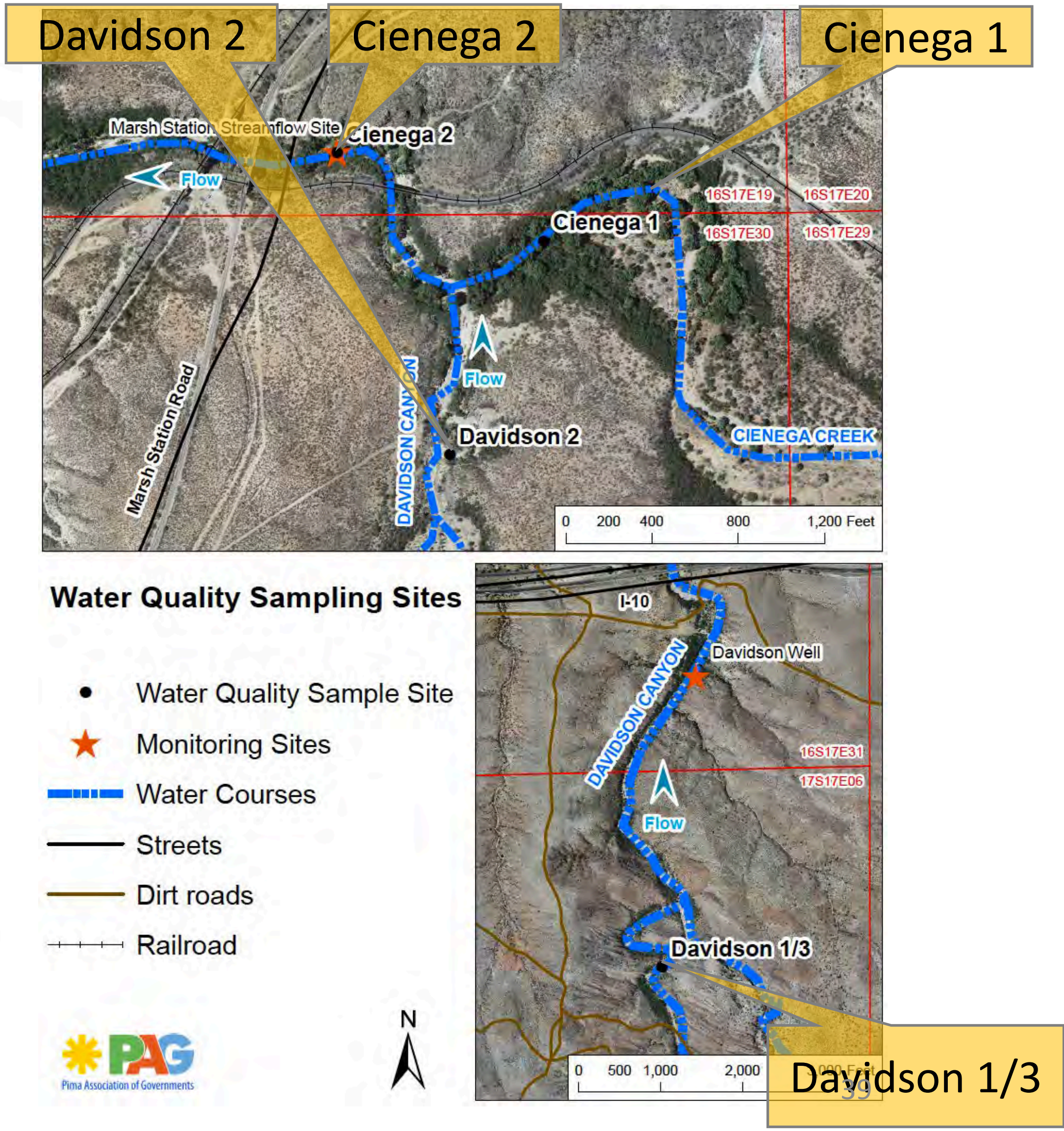

Source: Mead Mier, PAG

Water quality - BLM

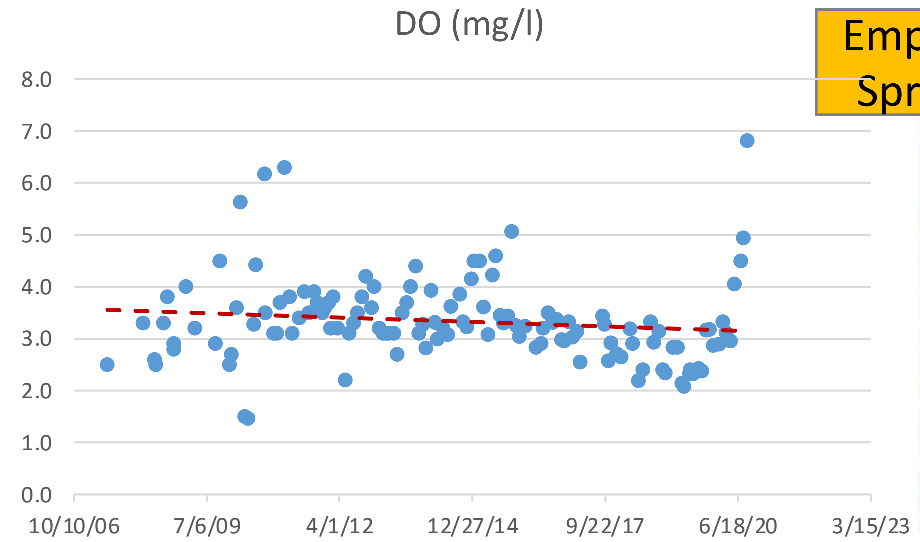

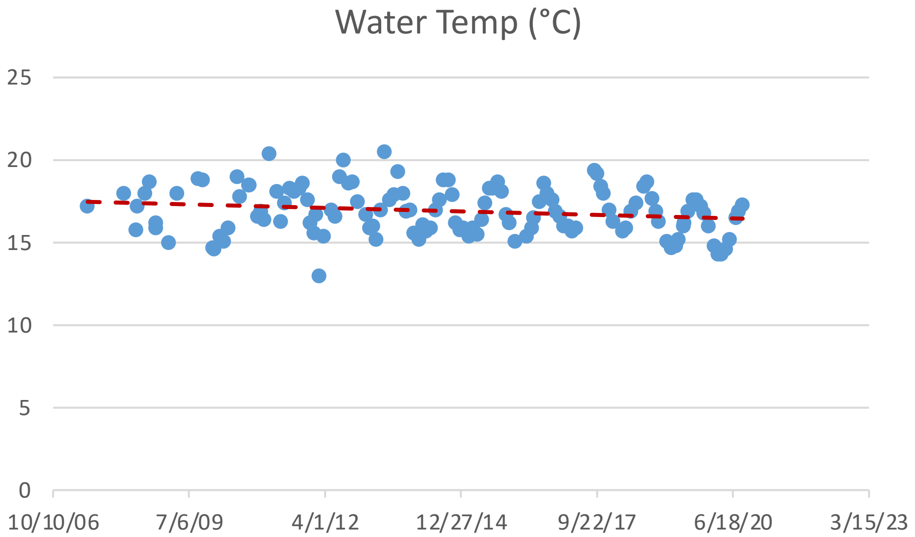

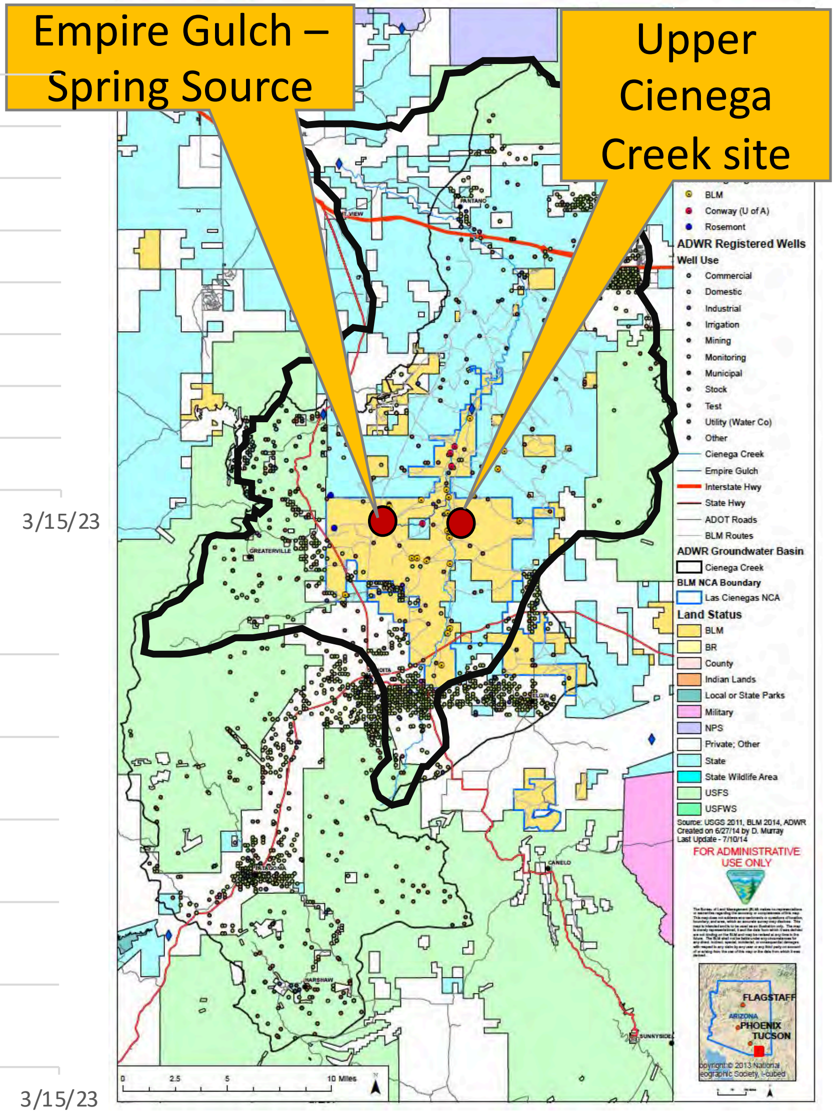

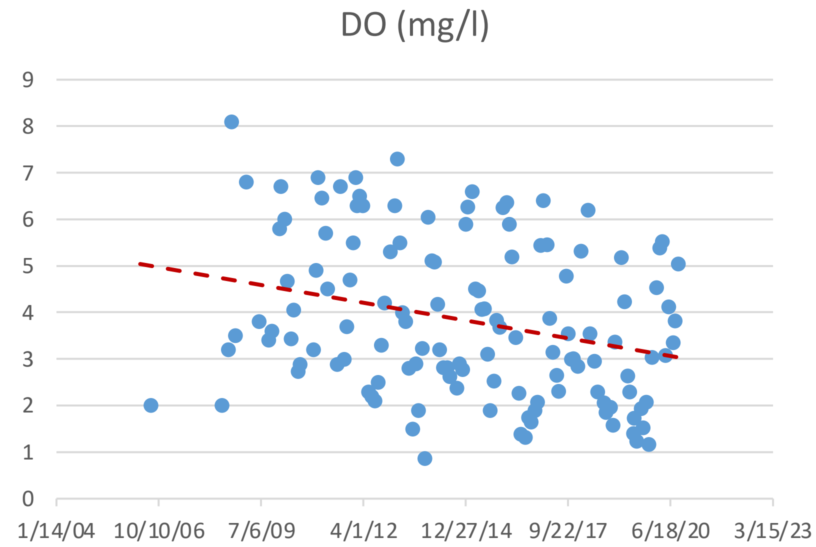

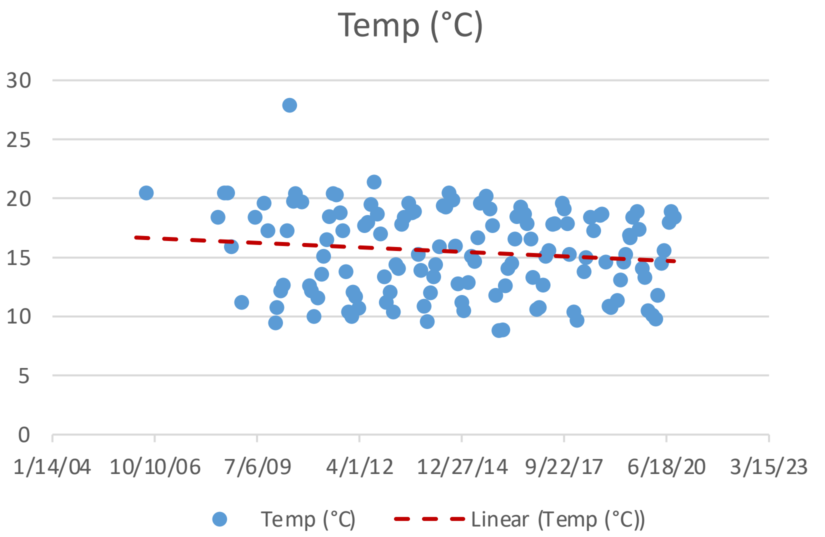

Source: Dave Murray, BLM

Water quality - BLM

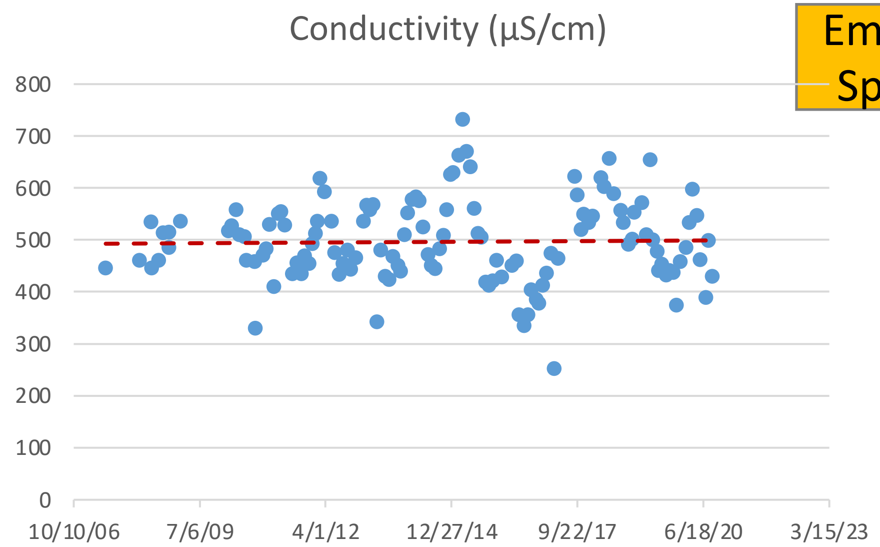

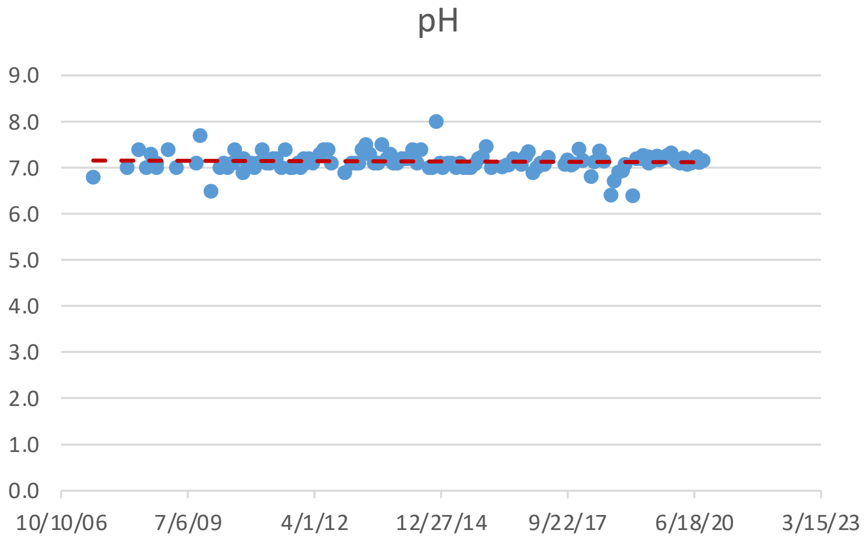

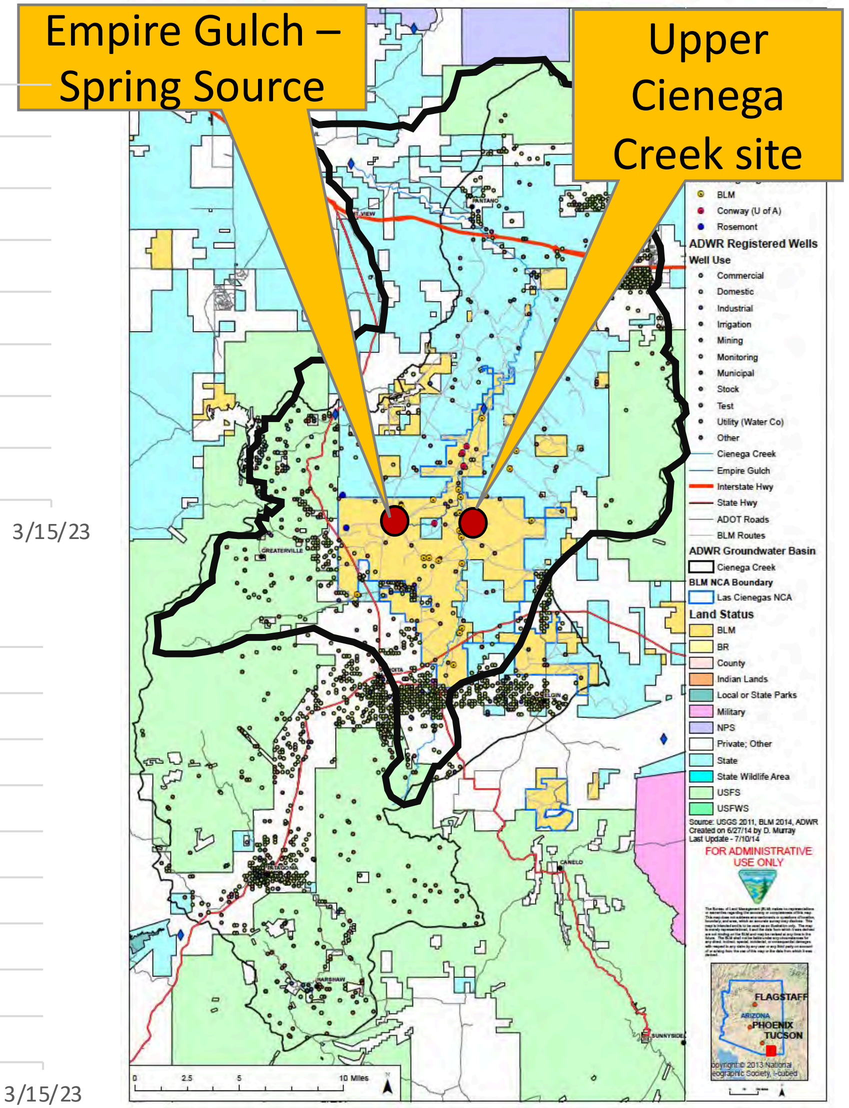

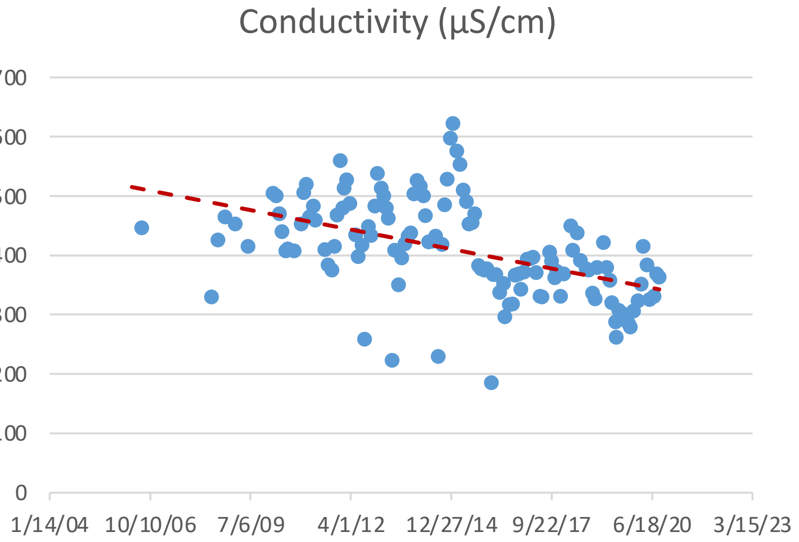

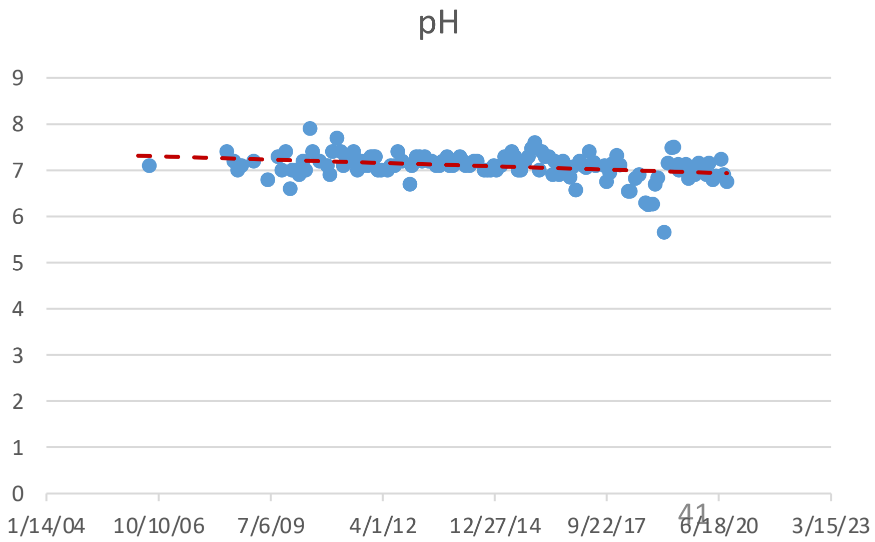

Source: Dave Murray, BLM

Water - general trends

- **Groundwater levels:**
  - Shallow wells show decrease in levels in the last 20 years
  - Deeper wells show slight decrease over time
- **Wetlands:**
  - Baseline created for upper watershed this year.
- **Wet-dry:**
  - Pima County: Significant decrease in minimum flow (June) since the 1980s, with slight recovery in 2016 but decrease since then.
  - BLM: Significant decrease in minimum flow (June) with slight recovery in 2015, but decrease since then.
- **Gauges:**
  - Pantano wash (Preserve): decrease in annual average stream flow over the past 25 years.
  - Cienega Creek (BLM): No trend

Water - general trends (cont.)

- **Winter stream flows:**
  - Preserve: Significant decrease in Marsh Station, slight increase in Titled Beds (after going dry during recent severe drought)
  - BLM: Slight increase in Empire Gulch, slight decrease in Upper Cienega Creek

- **Water quality:**
  - Pima County:
    - We see some seasonal variations but no major changes in annual trends
    - This baseline data is important to detecting impacts of any future change of land use (Rosemont Mine)
  - BLM:
    - Stable numbers in Empire Gulch, and decrease in all water quality indicators in Upper Cienega Creek

Ecological

Indicator	No.	Description
Veg. vol./comp./cover	9	Land cover
Wildlife	10	Pronghorn
	11	Fish
	12	Frogs
Fire	13	Coverage, severity

Vegetation composition – basal cover (BLM)

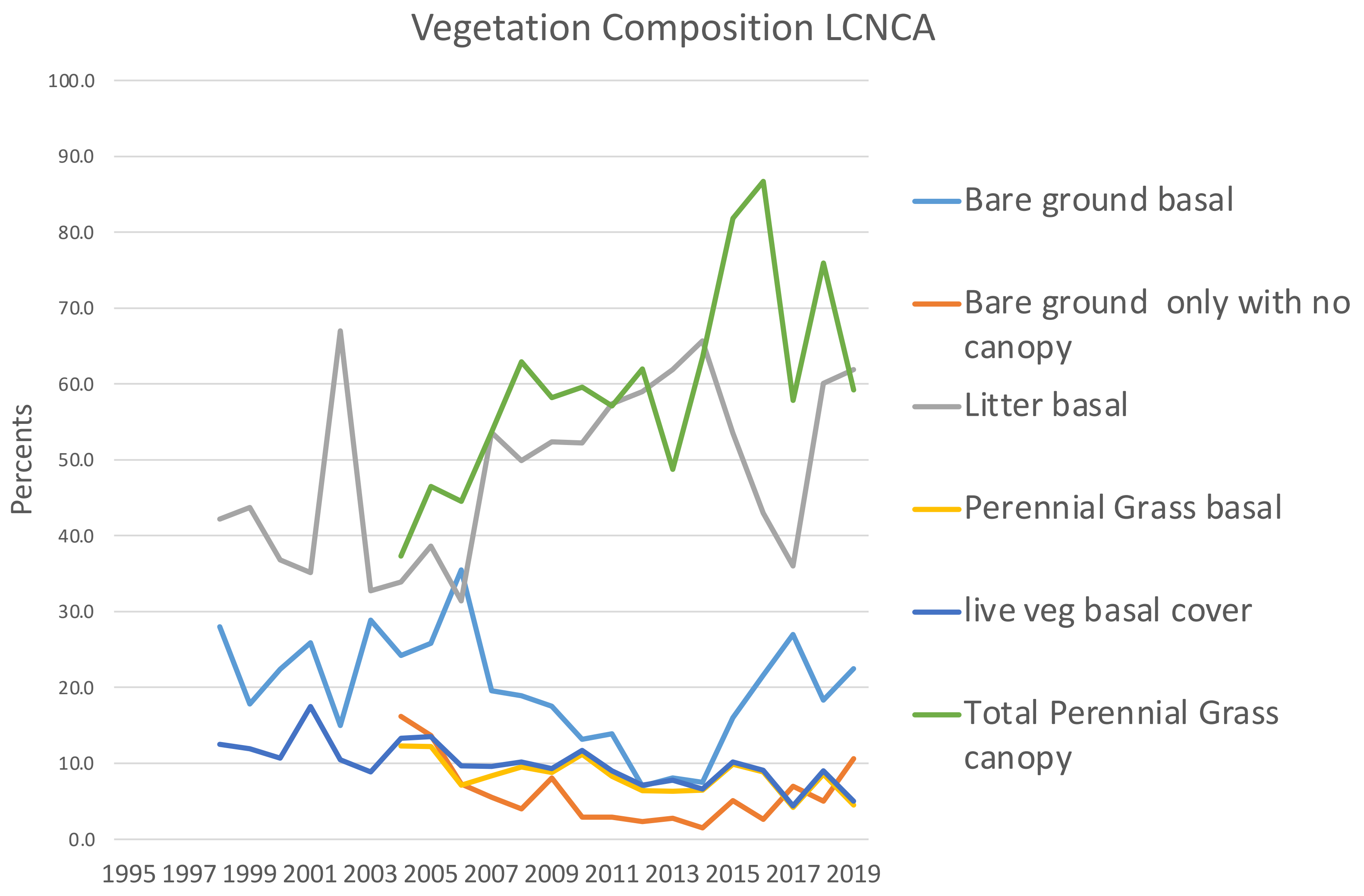

"2019 data is taken at random locations throughout LNCA, not at the exact locations where Gita's data was taken"

T. Condo, BLM

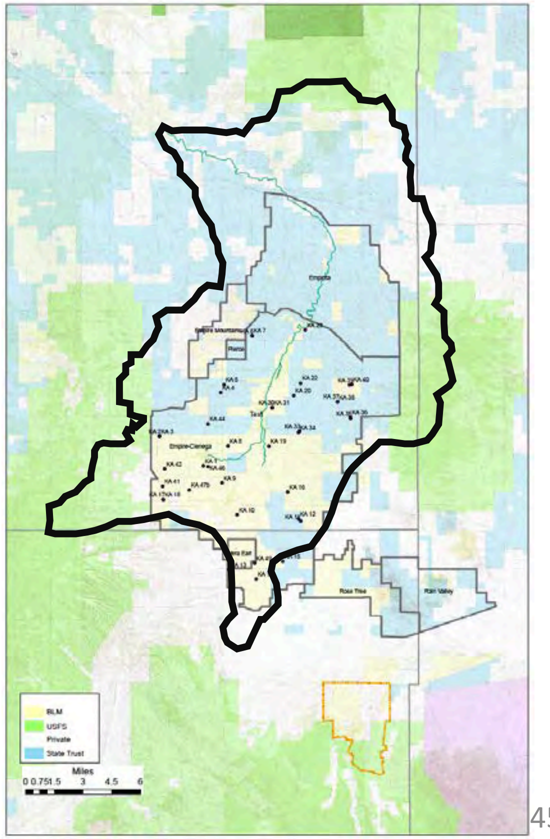

Source: Gita Bodner, The Nature Conservancy; Theresa Condo, BLM (for 2019)

Vegetation composition – basal cover and shrubs (BLM)

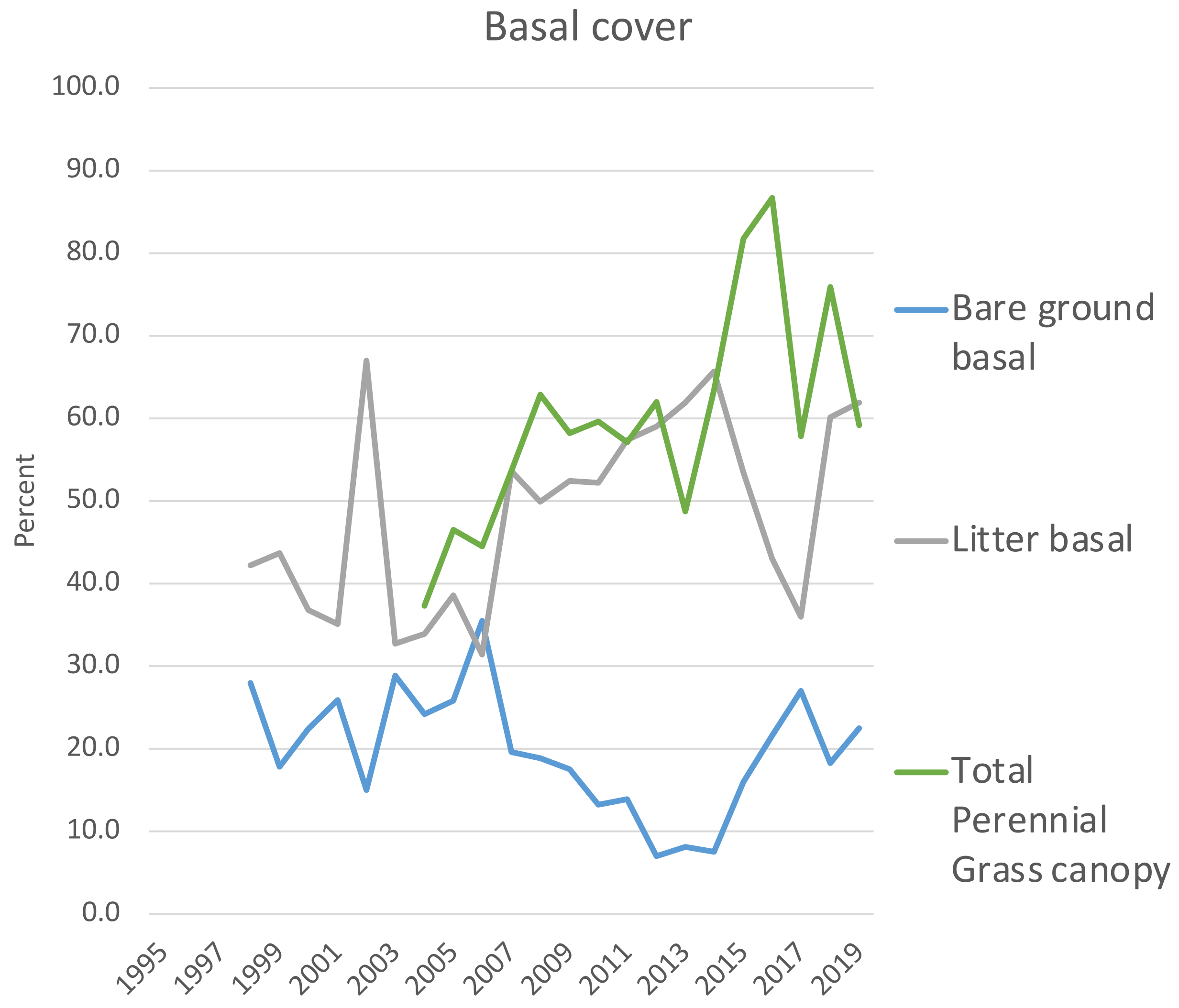

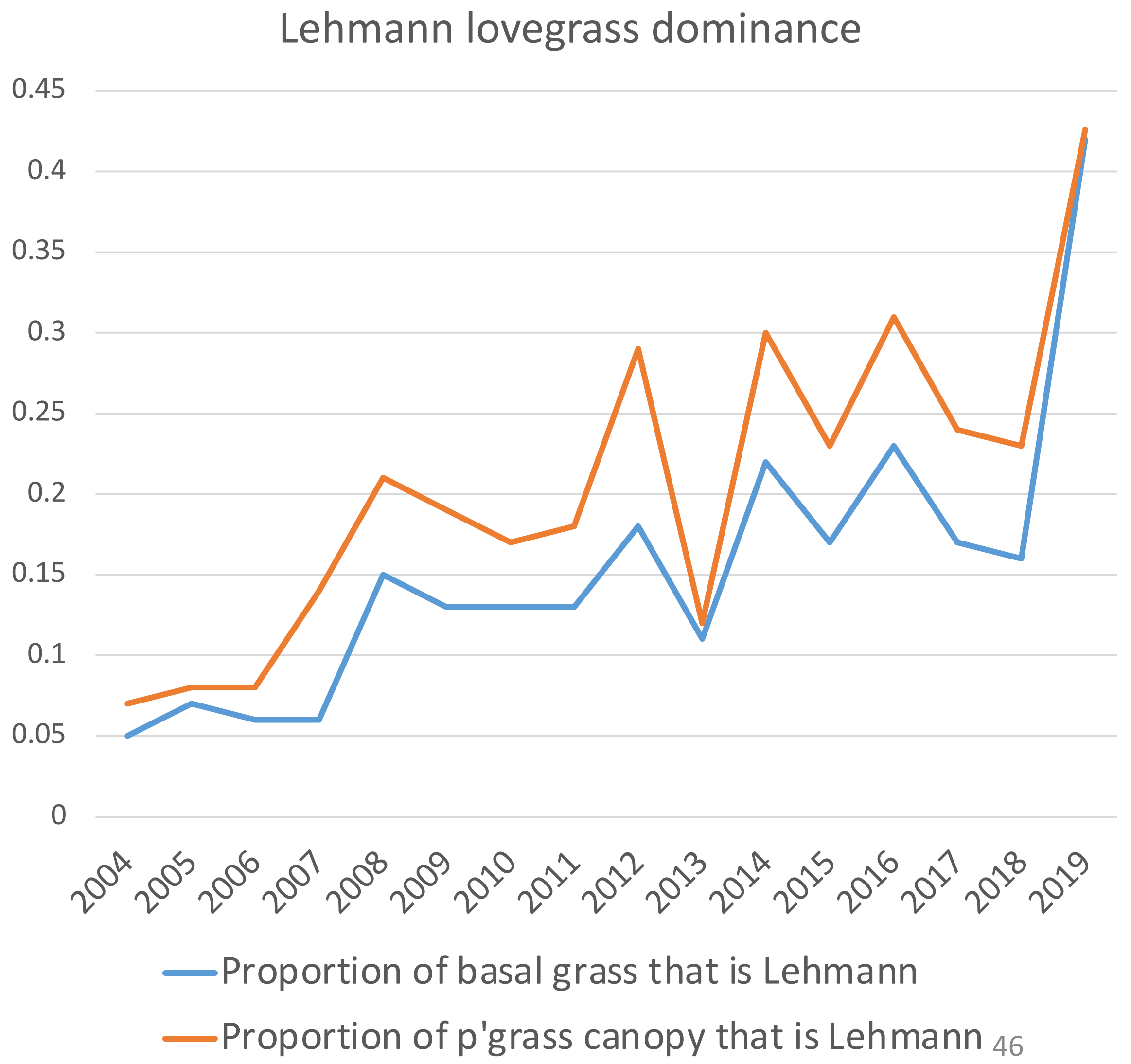

Source: Gita Bodner, TNC; Theresa Condo, BLM

Vegetation composition – Bar V Ranch, Pima County

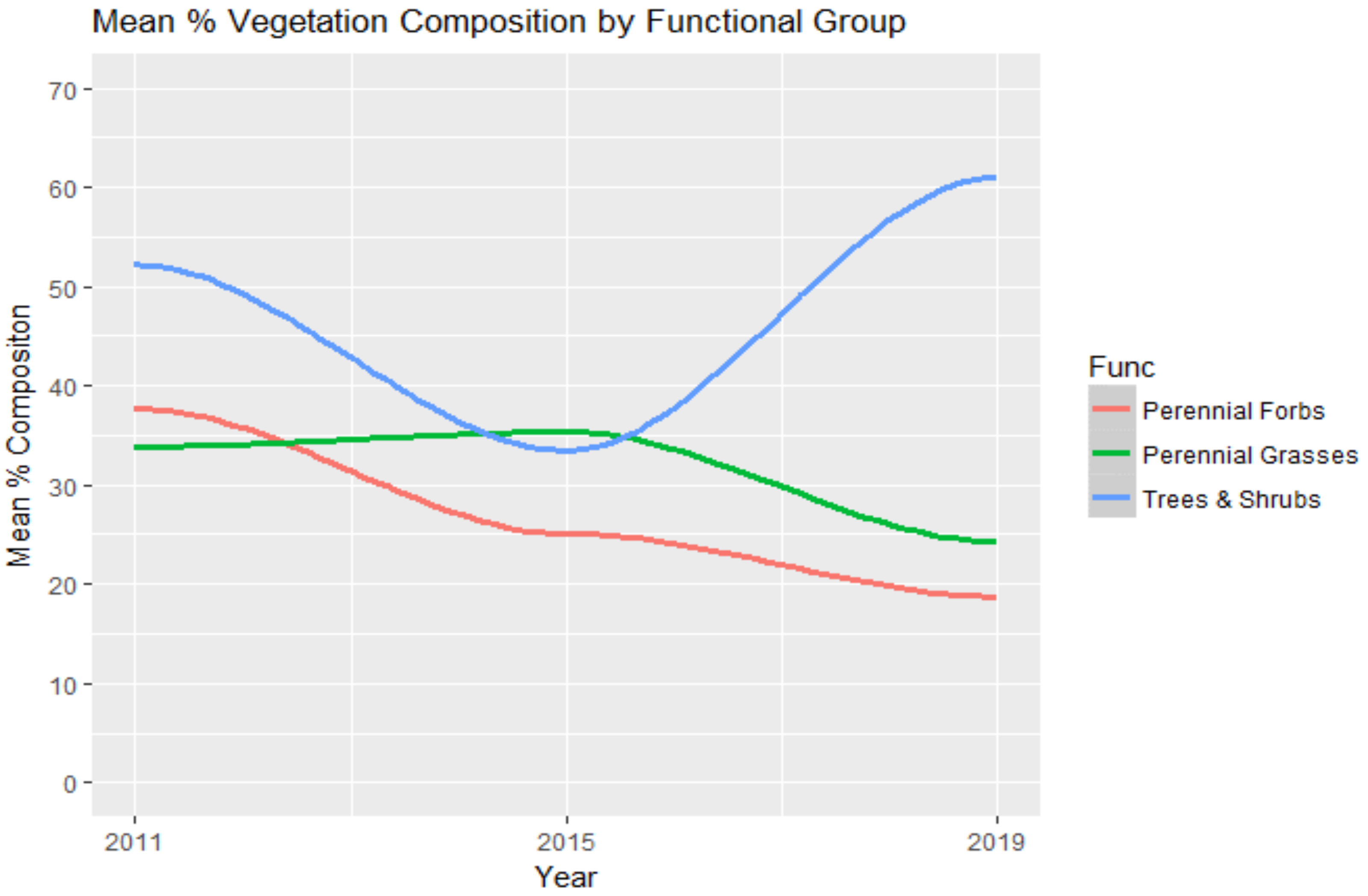

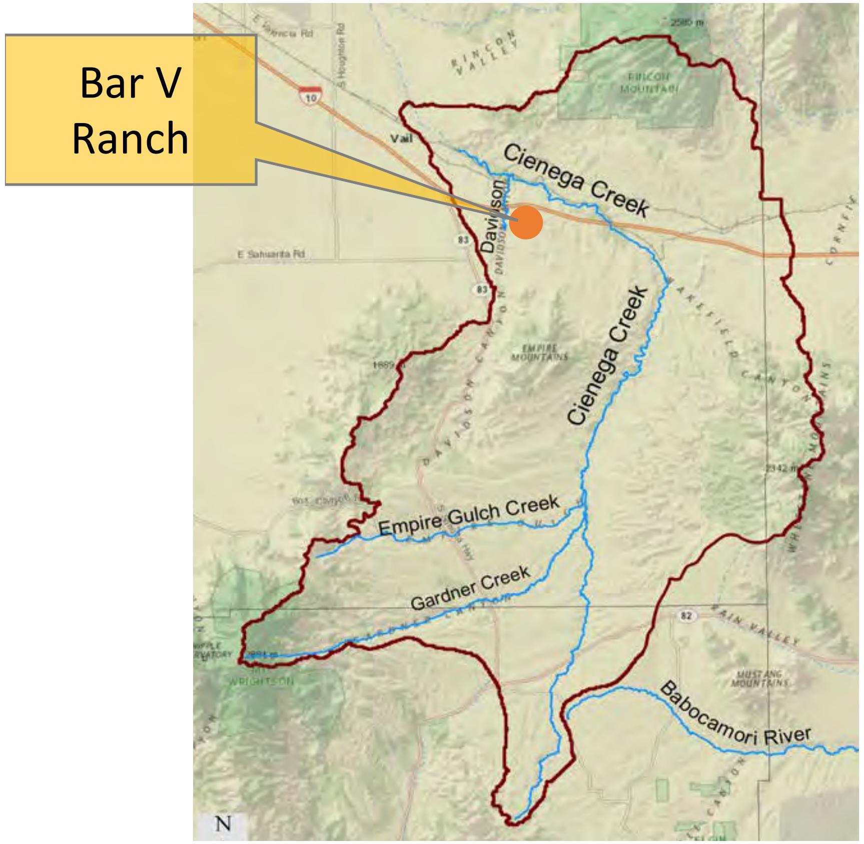

Source: Jeff Gicklhorn, Pima County Office of Sustainability and Conservation

Pronghorn

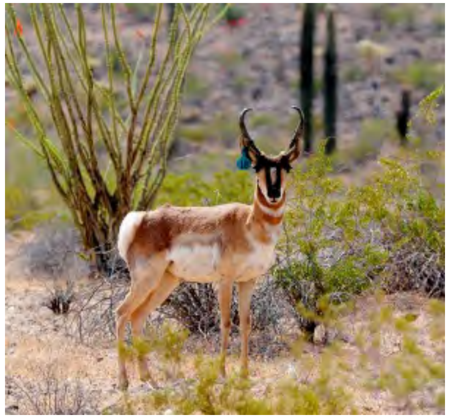

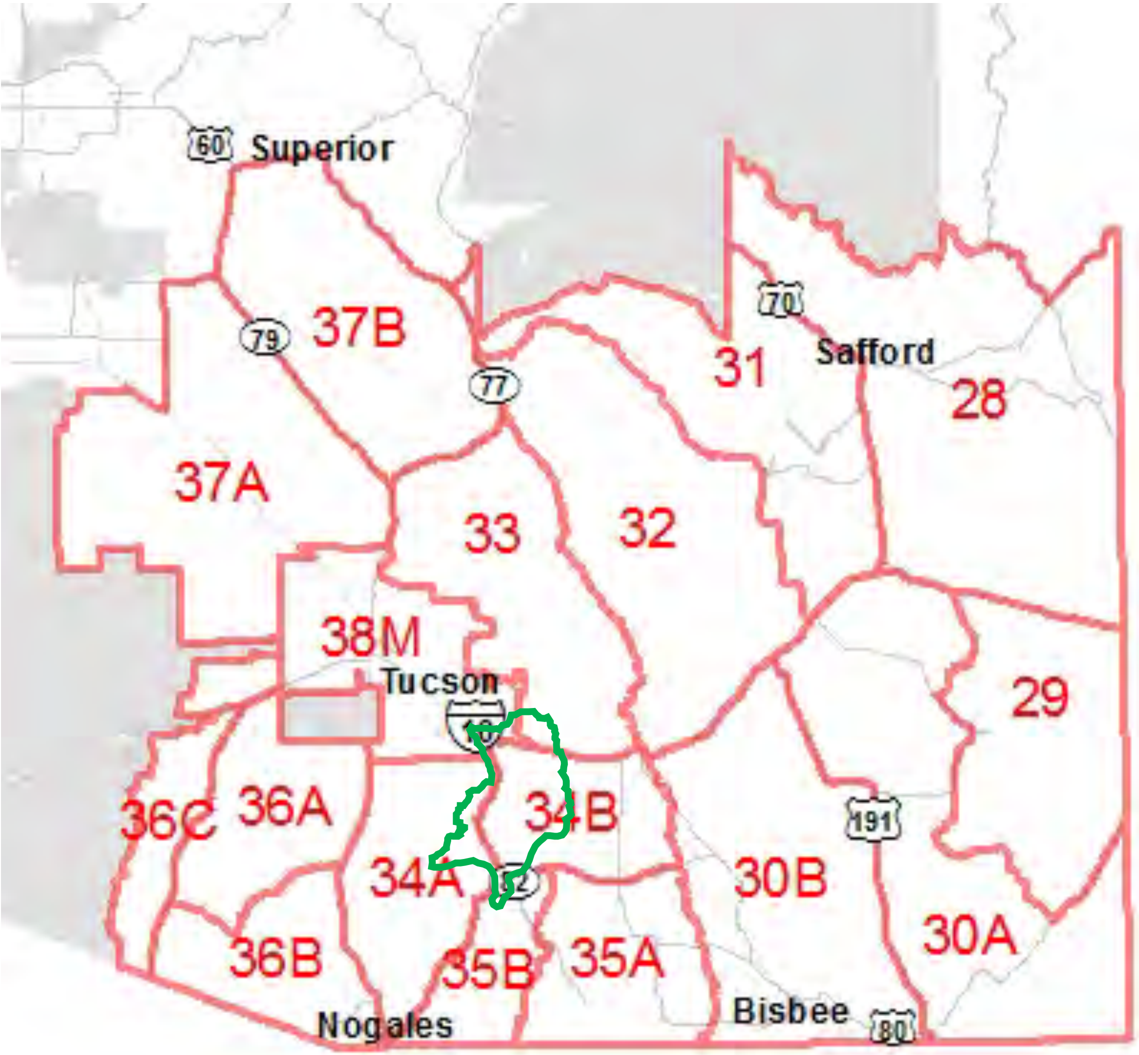

- The Cienega Watershed is mostly contained in section 34B

- Habitat fragmentation and mesquite encroachment are major threats to pronghorn.

Source: Karen Klima, Arizona Game and Fish Department

Pronghorn

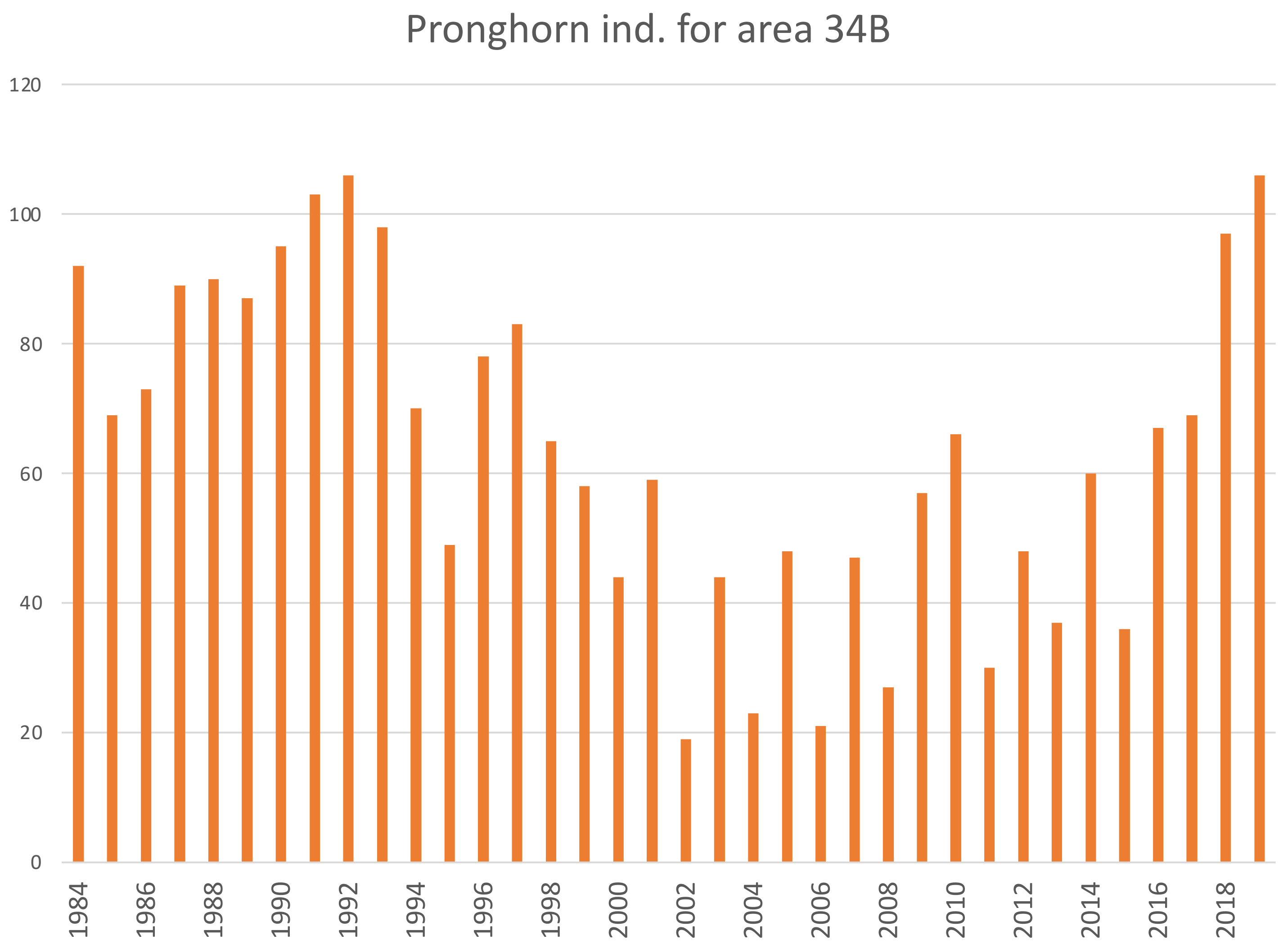

- Declines in population of pronghorn in 2002.
- Management responses:
  1. restricted hunting permits
  2. control measures on predator population (coyotes)
  3. install and maintain water tanks
  4. modify fences
- After management, pronghorn population in unit 34B appears to be slowly increasing
- In recent years, AZGFD has seen pronghorn moving across hwy 83 into polygon 34A (Vera Earl Ranch). These individuals are part of the same herd from 34B.

Source: Karen Klima and Rana Tucker, Arizona Game and Fish Department

Fish

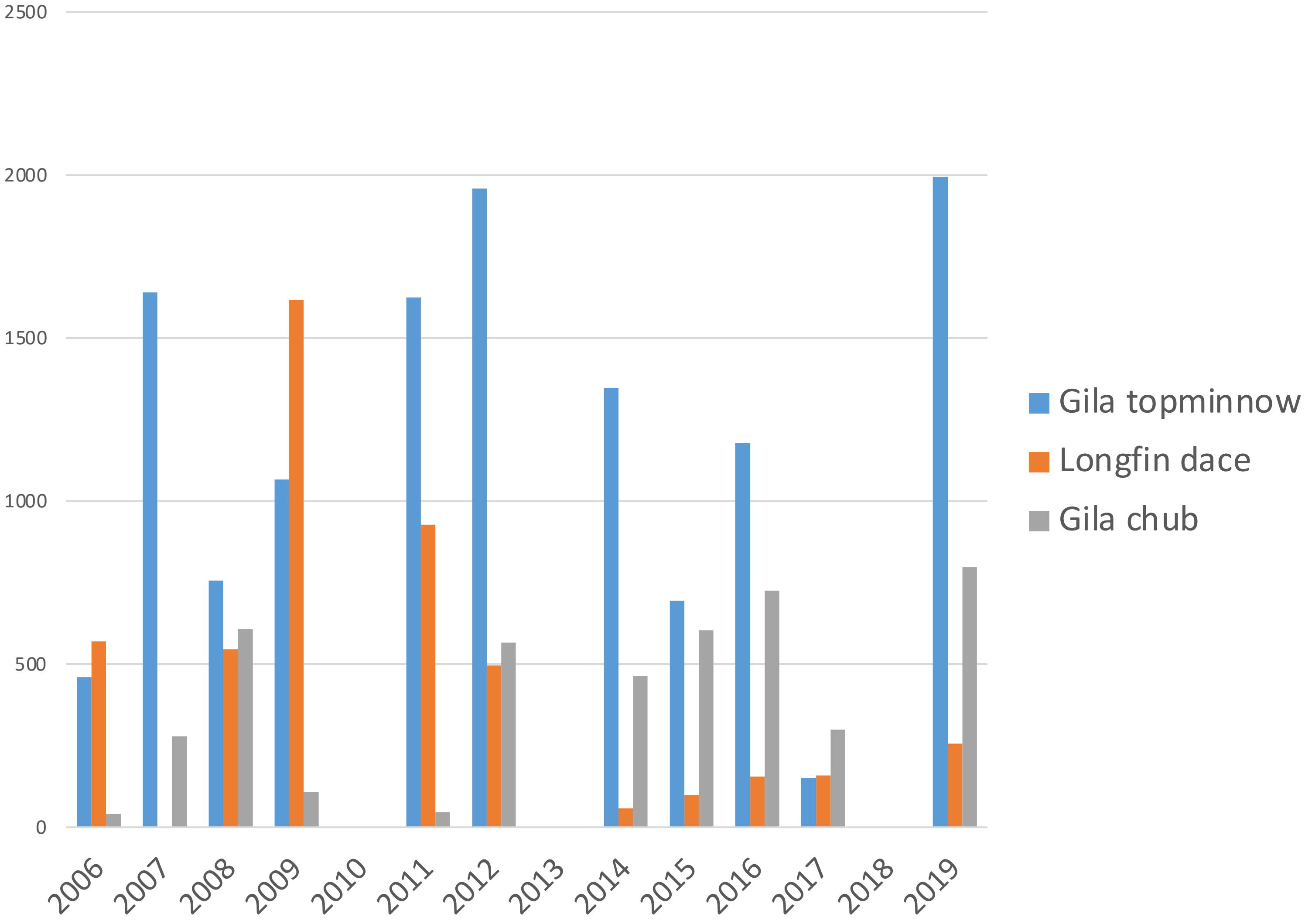

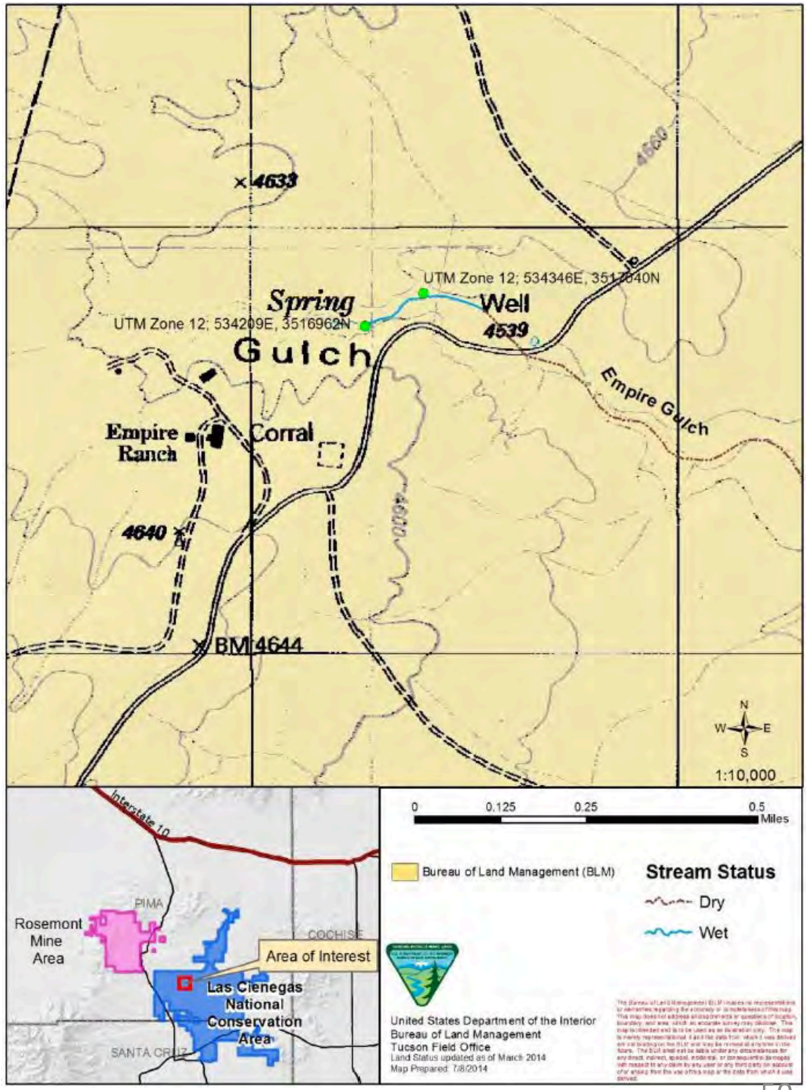

Source: Jeff Simms and Christina Pere from BLM

Frogs

- AZFWS introduced bullfrogs into Arizona from 1930's into early 1980's to increase sport fishing opportunities. By the late 1980's bullfrogs began to invade into native frog ranges and exclude native frog species by predation.
- Bullfrogs were first reported on Empire Ranch in the 1980's and this correlated with native frog declines in the area.
- By 2004 the Chiricahua leopard frog had declined throughout its range and was federally listed as a threatened species. Chiricahua leopard frog reduced to occupying only one site on the LCNCA.
- In 2010, intensive frog conservation efforts began in the area.

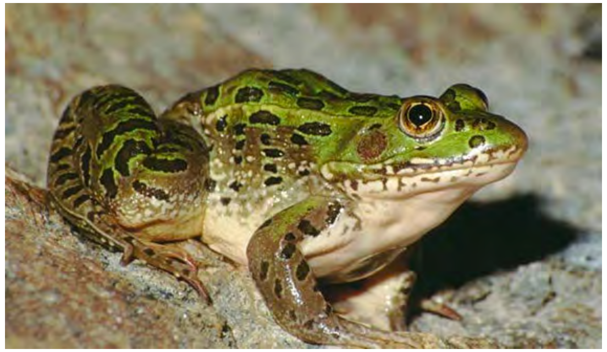

Chiricahua leopard frog – native threatened species

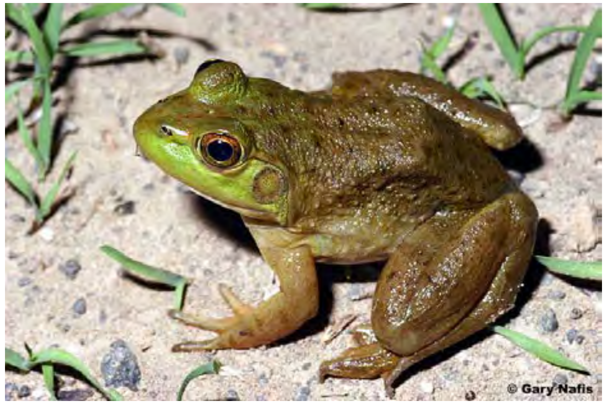

Bullfrog – non-native/invasive species

Source: David Hall, CWP-Frog Project

Frogs

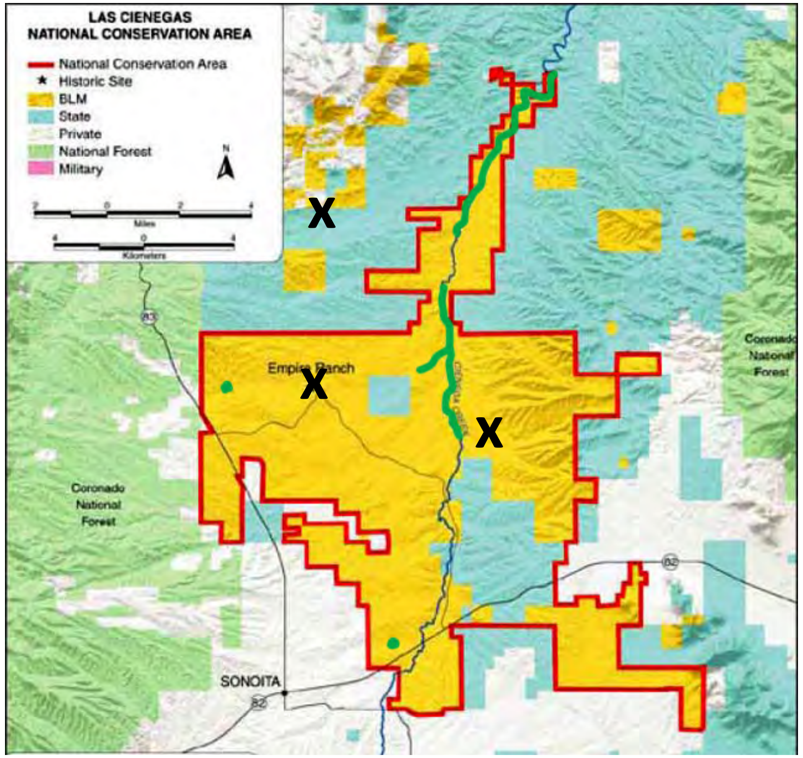

1980's Geographic distribution of Chiricahua Leopard frog (green line) Black Xs show bullfrog records

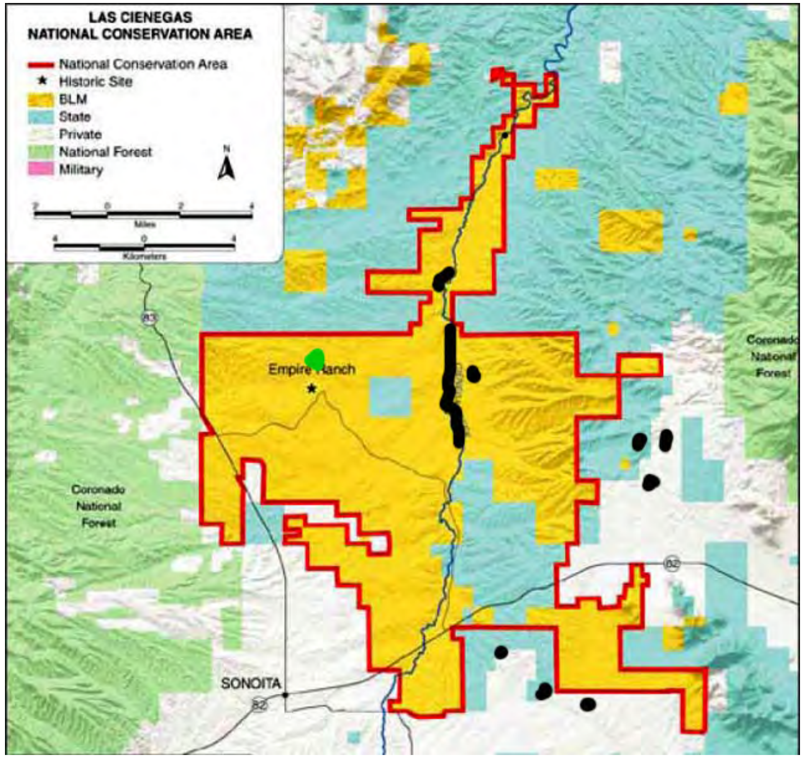

2010 Geographic distribution of Chiricahua Leopard frog (green point) Black lines show bullfrog records

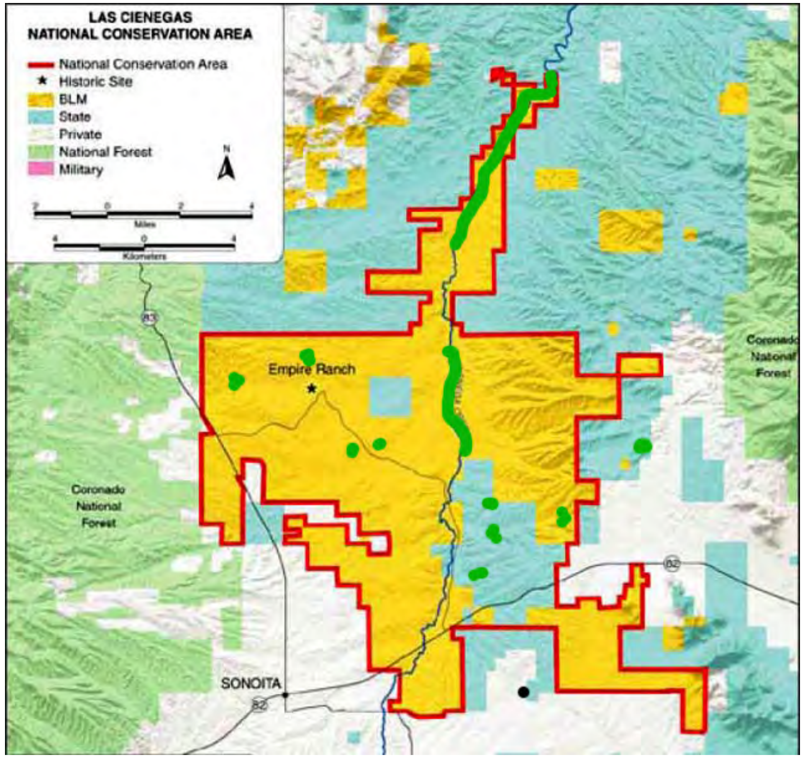

2016 Geographic distribution of Chiricahua Leopard frog (green line and points). Black point show current bullfrog distribution

Source: David Hall, CWP-Frog Project

Frogs

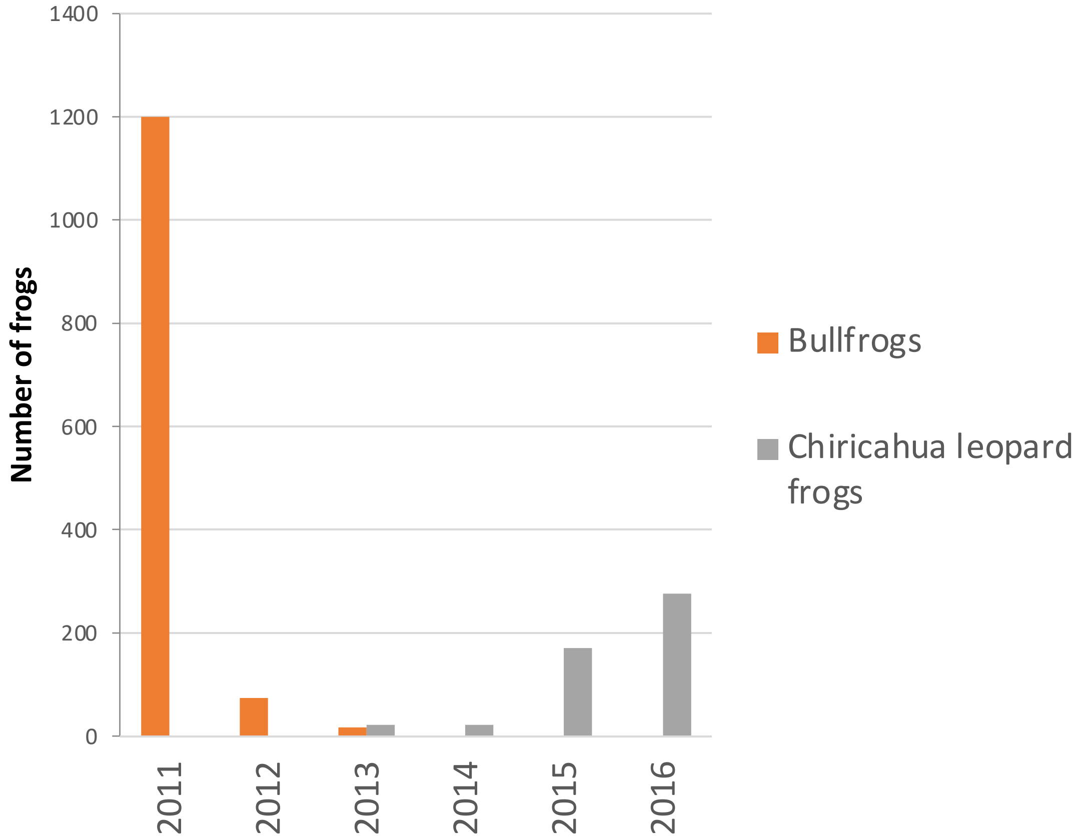

Management actions include:

- Removal of invasive species
- Creation of buffer zones that consist of stock water tanks that are wetted during the monsoon season. Then they monitor the tanks during spring and summer months and remove any bullfrogs found.
- Efforts involve the collaboration between federal agencies:
  - AZGFD
  - BLM
  - USFW
  - USFS
- Engagement with private land-owners to work in their land, or creating a buffer zone around it.

Source: David Hall, CWP-Frog Project

Wildfires

345.4 acres were burned in 2019

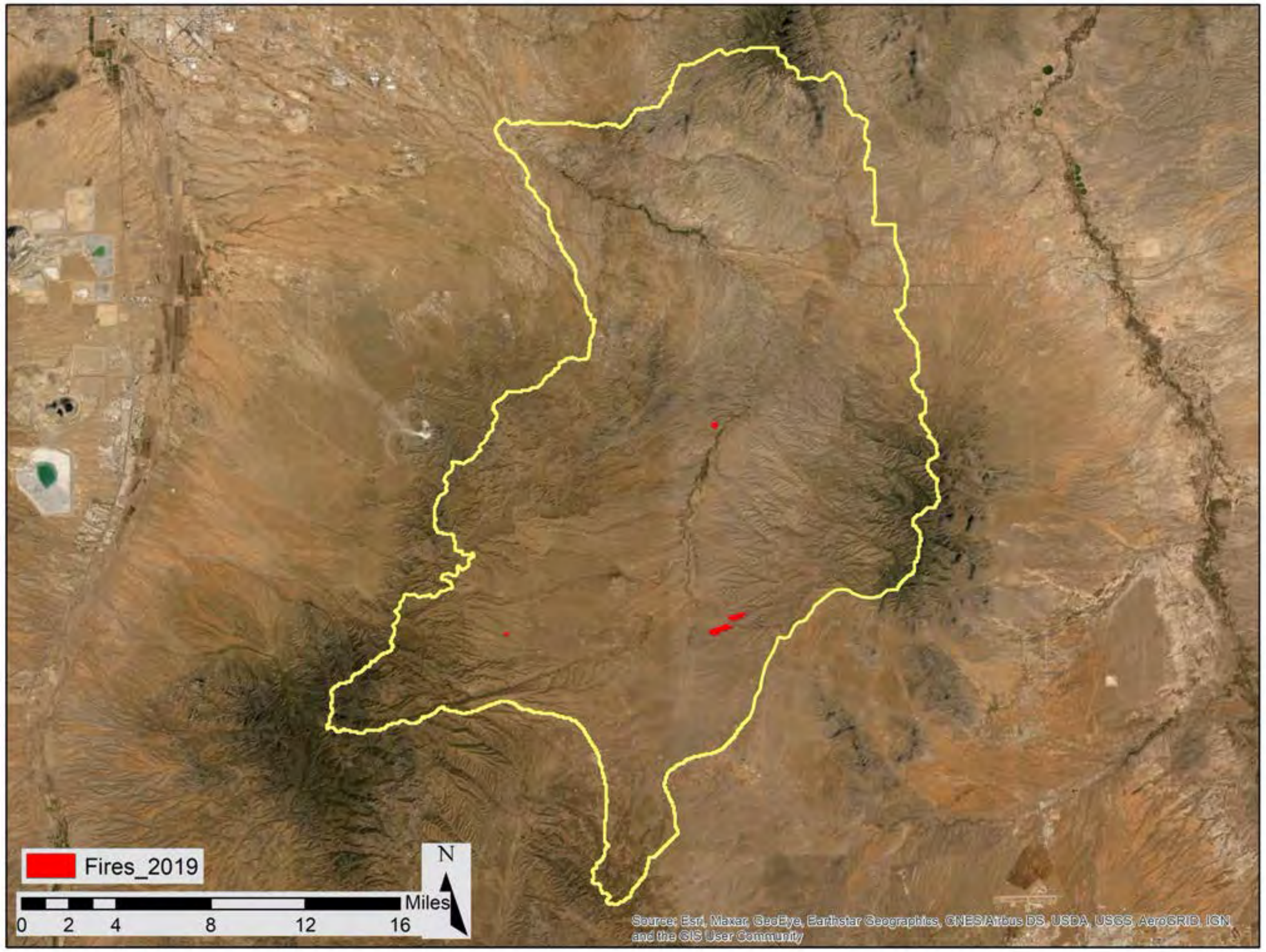

Source: http://wfdss.usgs.gov/wfdss/WFDSS_Data_Downloads.shtml
Fires from 2018 and 2019: Dan Quintana and Zach Driscoll - BLM

Wildfires

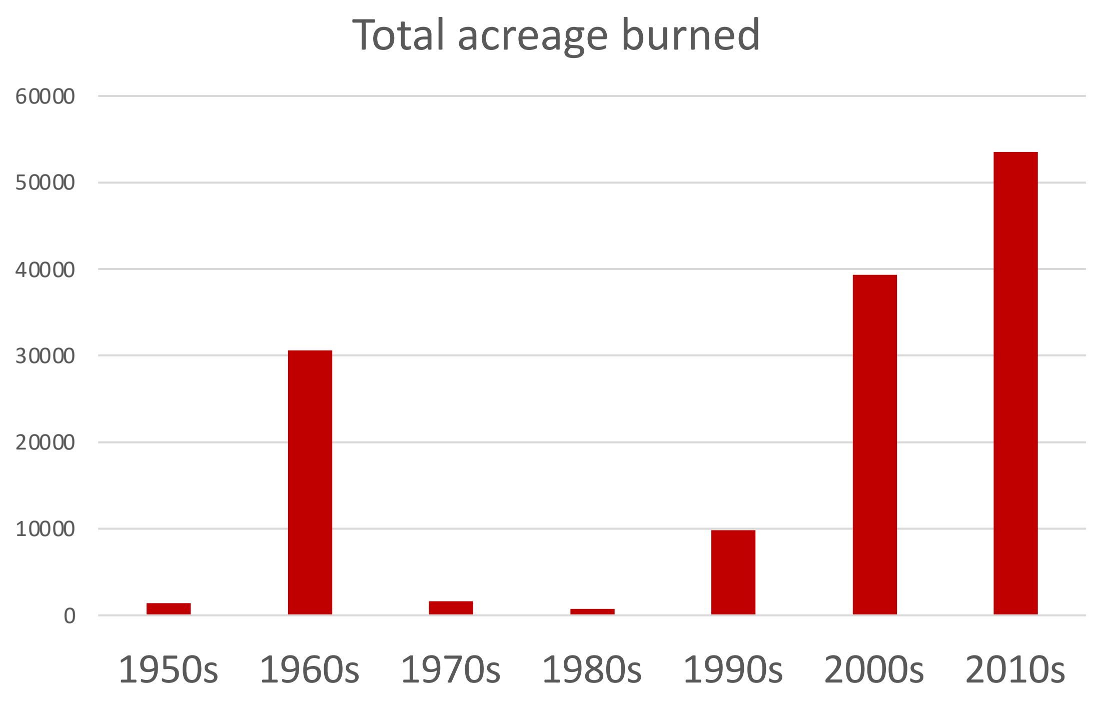

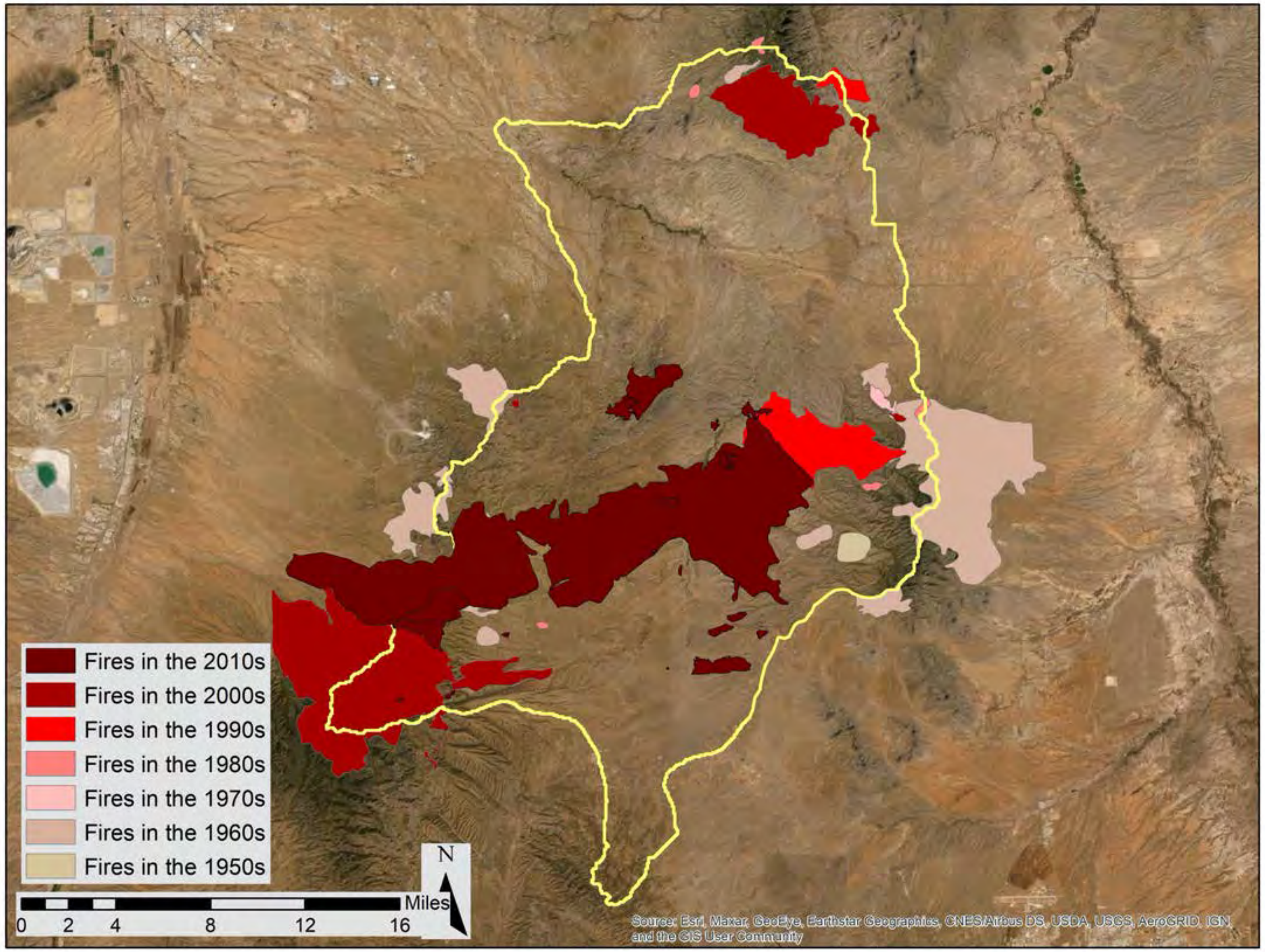

Source: http://wfdss.usgs.gov/wfdss/WFDSS_Data_Downloads.shtml
Fires from 2018 and 2019: Dan Quintana and Zach Driscoll - BLM

Ecological indicators - General trends

Vegetation composition

- **Perennial grass** basal cover has declined, but 2015 and 2016 have shown some recovery.
- **Invasive perennial grasses** (e.g., Lehman lovegrass) have shown increase. There is an unusual relationship between cover data: basal area is generally down, while canopy is generally up. This may be in large part due to Lehman lovegrass.
- **Bare ground inc**reased in 2017, while the amount of **litter** has decreased.

This means that

- Mesquite removal has been successful in decreasing shrub species cover and density
- The trend re perennial grass is unclear, but invasive species remain a threat
- Soil erosion may be decreasing because there is less bare ground and more litter

Ecological - General trends

Pronghorn:

- Earlier decline in pronghorn population (2002) has triggered management actions that have resulted in population recovery

Fish:

- Gila Topminnow has recovered significantly in 2015
- Gila chub also shows slow recovery
- Longfin dace has steadily increased

Frogs:

- Successful recovery of listed species and reduction of invasive species since 2013

Wildfires:

- Significant acreage burned in 1960s. Dramatic increase in acreage burned in 2000s. Damage from recent fires is significant and costly.

Socio-cultural

Indicator	No.	Description
**Economic vitality**	15	Median household income, median home values, unemployment, residents below poverty level
**Land-use land-cover change**	16	Land use and land cover change
**Number of wells**	17	Number of wells installed within the watershed and buffer area of 10 mi
**Archaeological site conditions**	18	Trend in site conditions, both human and natural-caused damage.
**Number of recreational permits**	19	Number of recreational permits over time
**Stewardship engagement programs**	20	Number of opportunities for active engagement

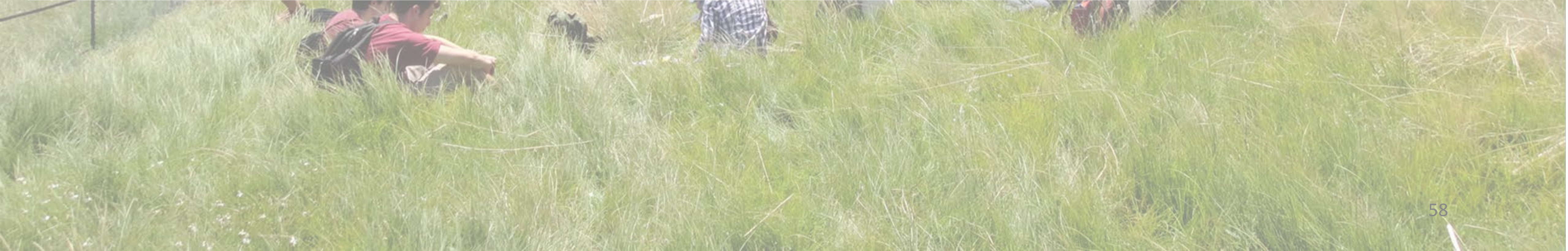

Population density and growth

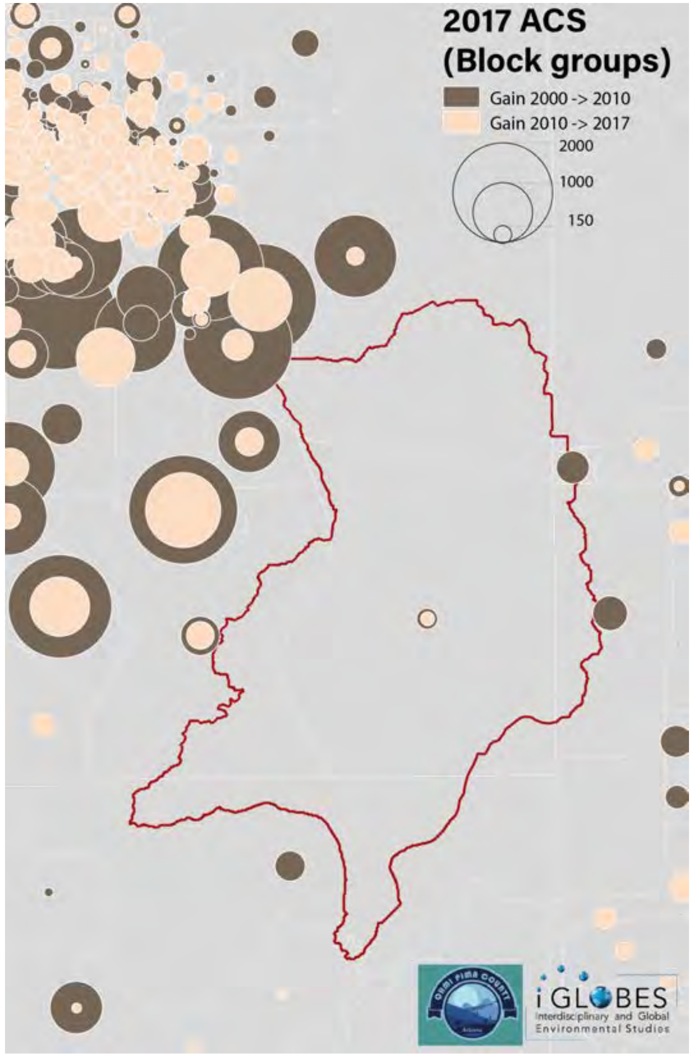

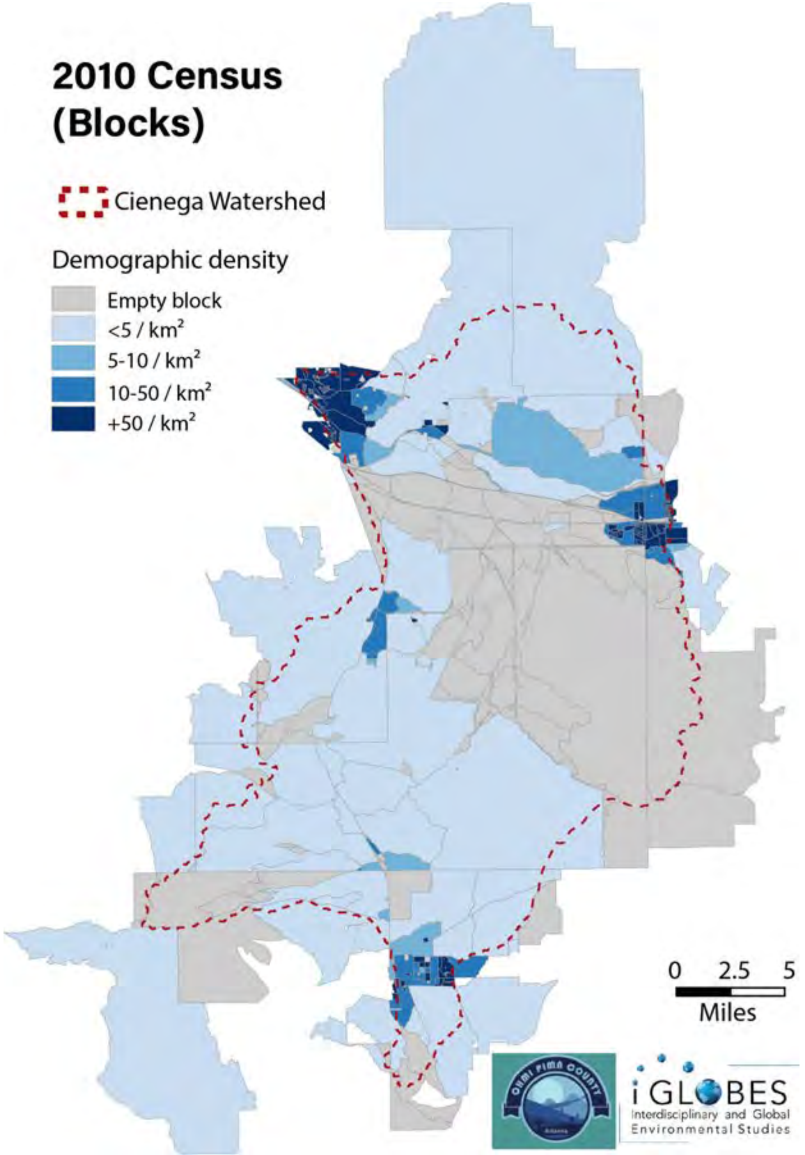

Source: Francois-Michel Le Tourneau, iGlobes OHMI Pima County

Land use – land cover

French team and Laura Norman (USGS) are working on Land Use in 2019

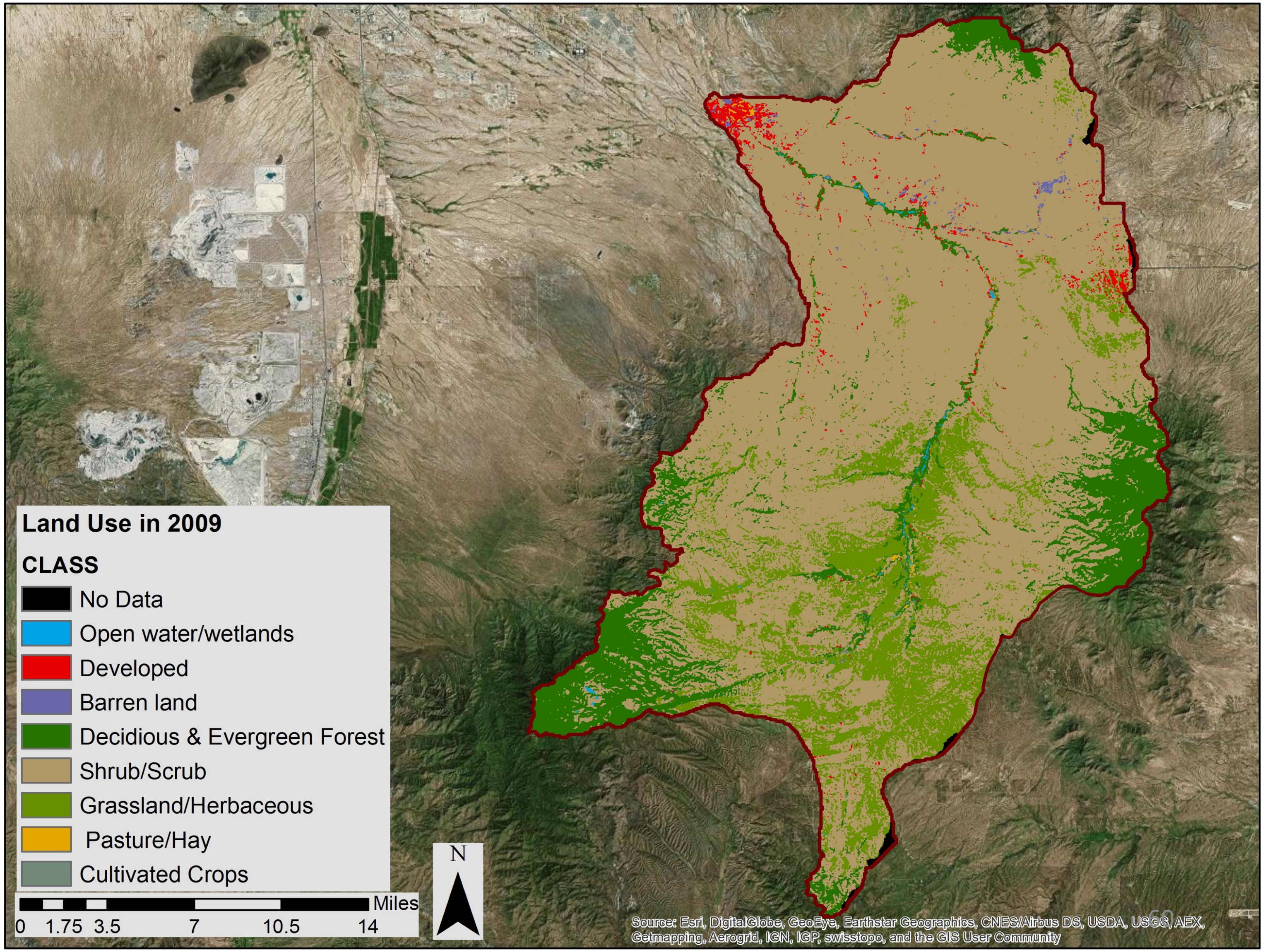

Source: Laura Norman, USGS.
https://pubs.usgs.gov/of/2011/1131/
Advise: Kyle Hartfield, ARSC

Land use – land cover

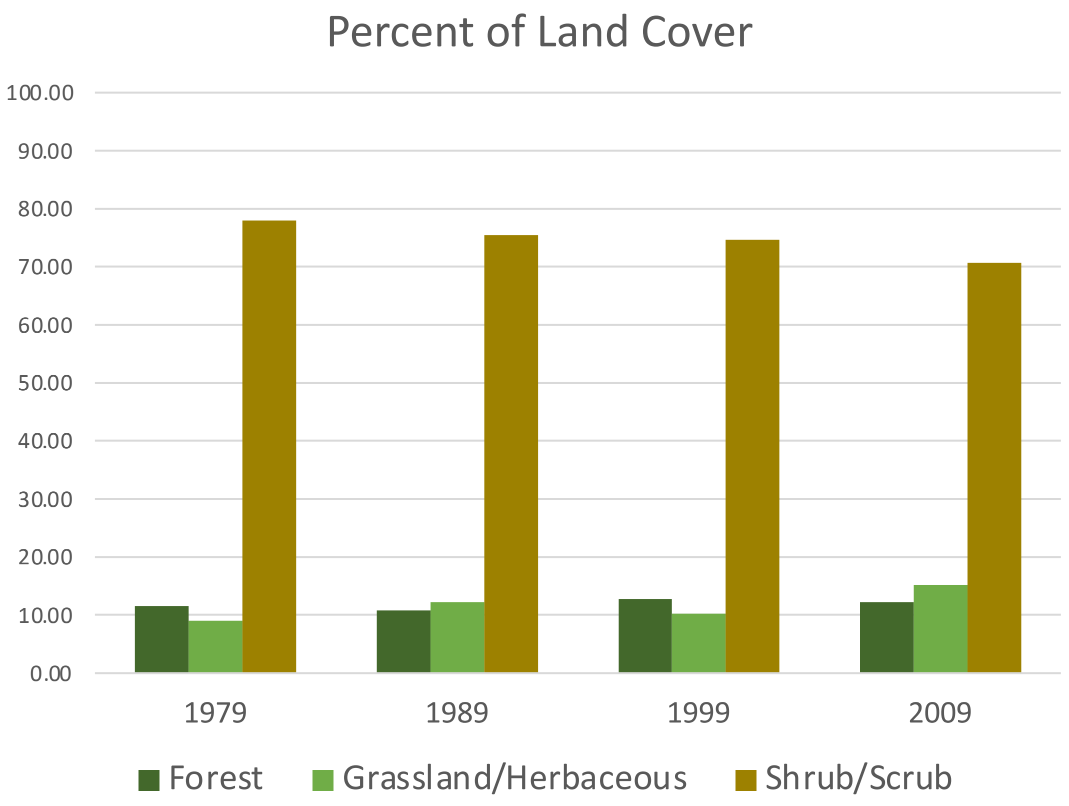

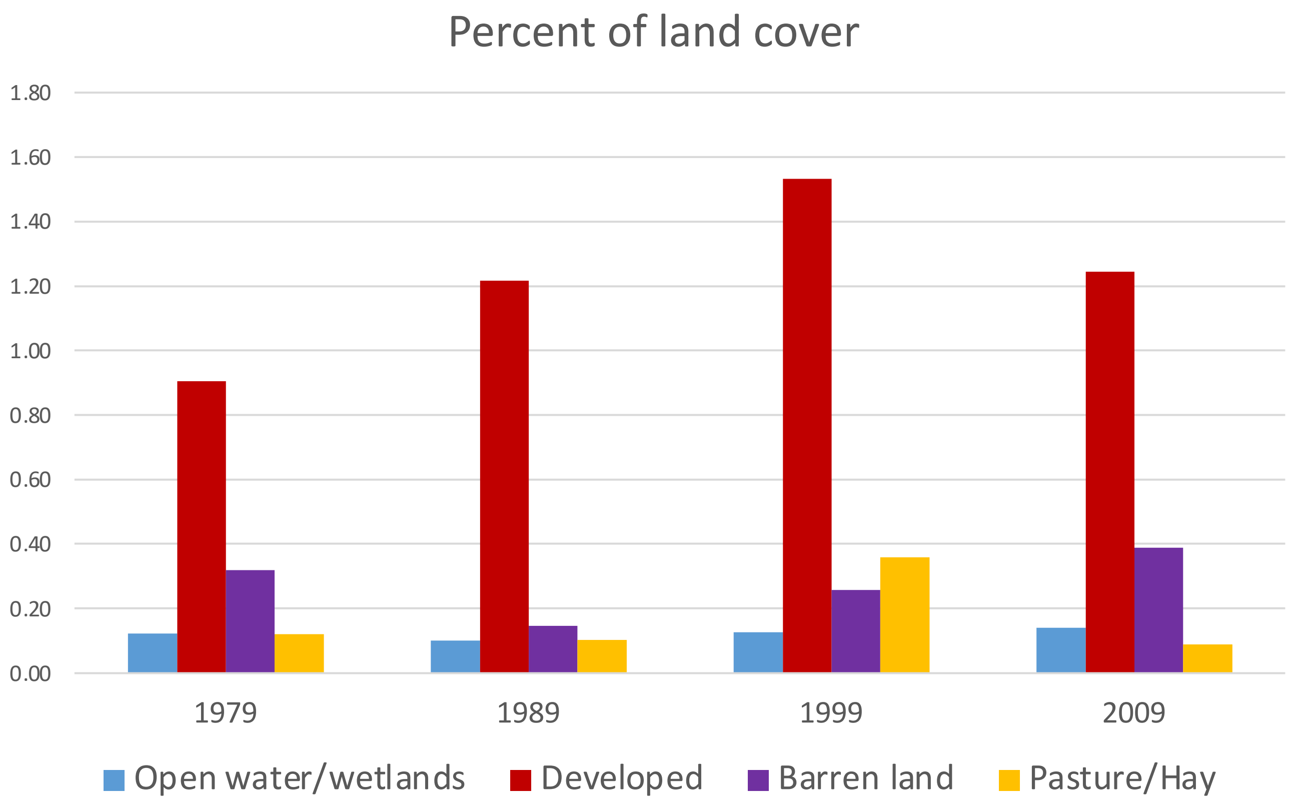

Source: Laura Norman, USGS. https://pubs.usgs.gov/of/2011/1131/
Advise: Kyle Hartfield, ARSC

Wells

25 new wells were installed in 2019

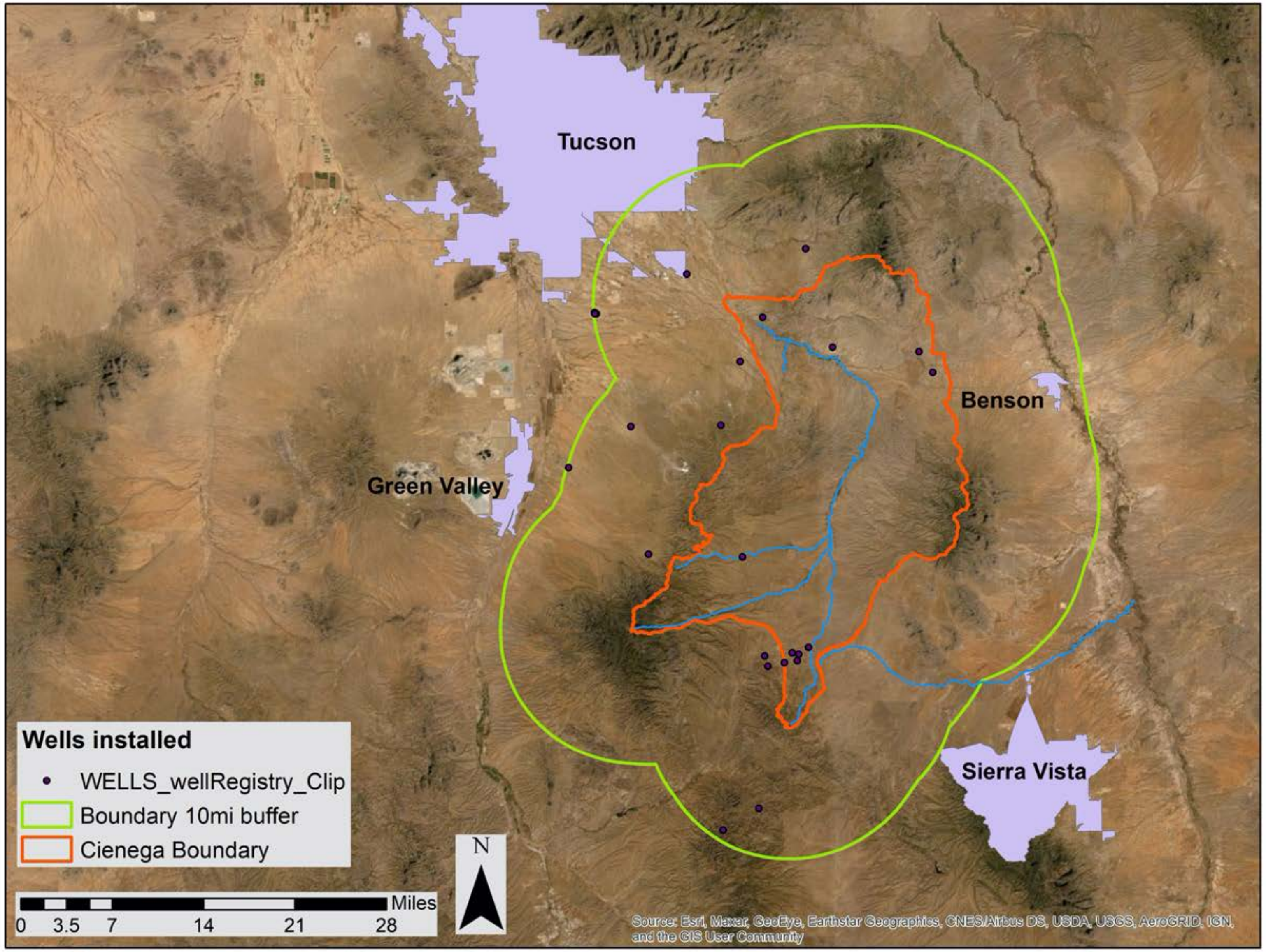

Source: Arizona Department of Water
Resources: http://www.new.azwater.gov/gis/
Advise: Kyle Hartfield, ARSC

Wells

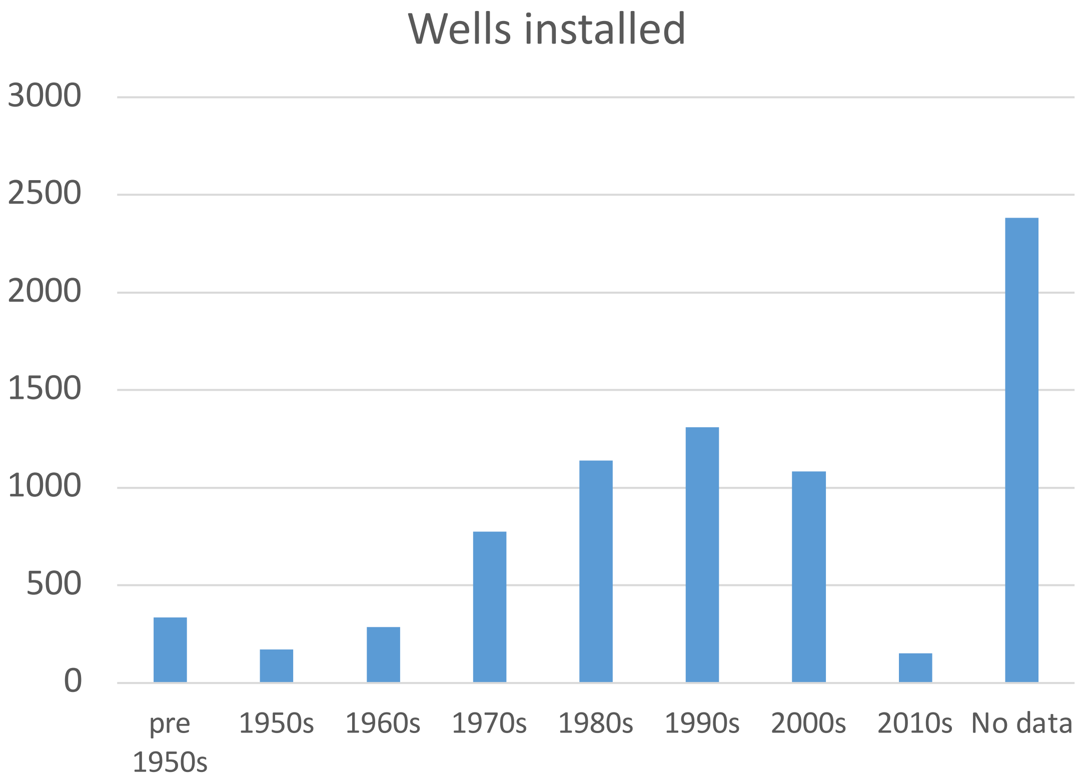

There are 7,640 wells in the Cienega Watershed

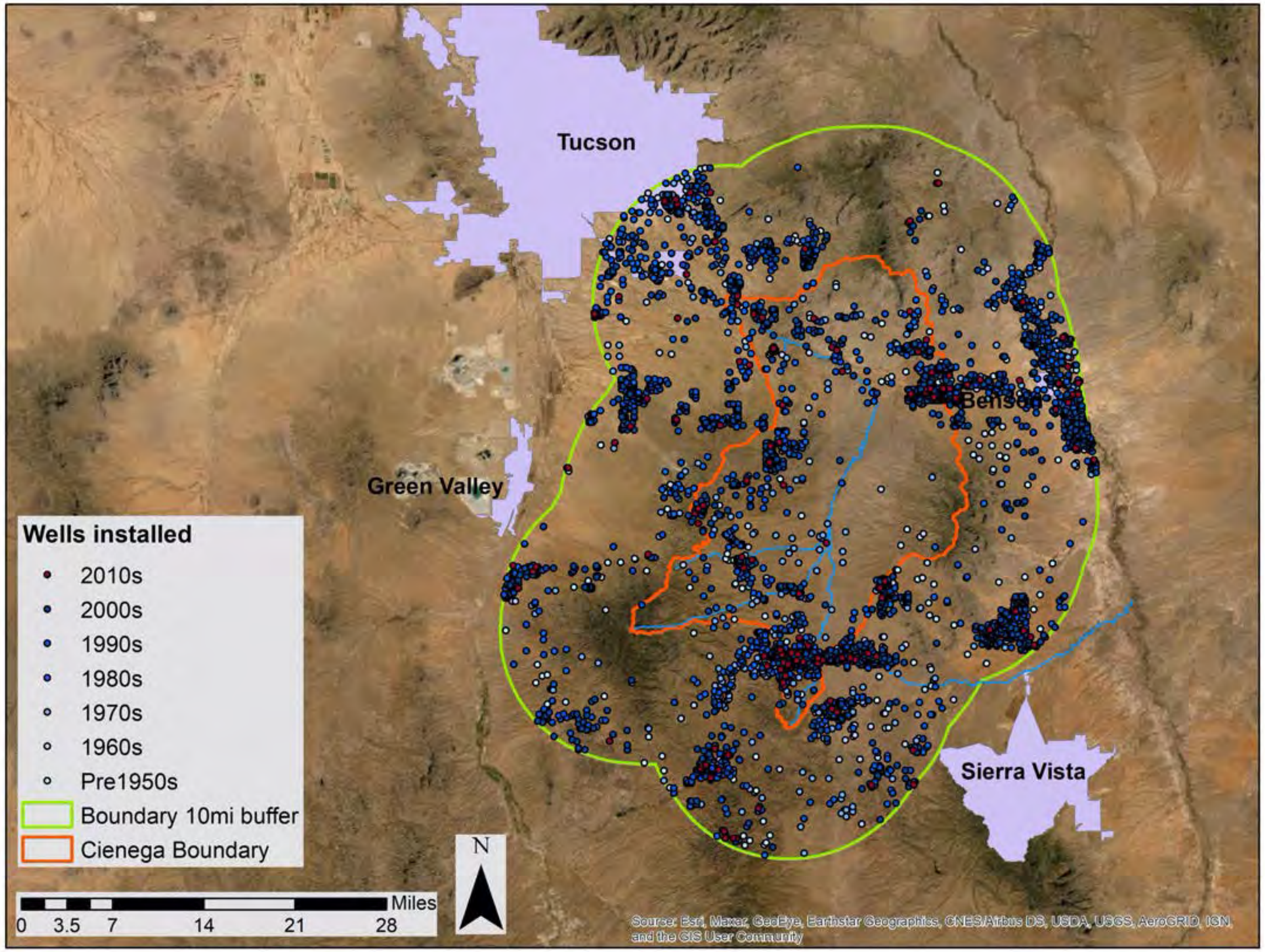

Source: Arizona Department of Water Resources: http://www.new.azwater.gov/gis/
Advise: Kyle Hartfield, ARSC

Archaeological Sites Condition

To establish trends in the conditions of selected sites, in 2017 watershed archaeologists began exploring shared standards for monitoring which identify both natural and human caused impacts.

Common characteristics to be collected and shared:

- Geographic zone
- Resource type
- National Register status
- Management status
- Resource condition
- Types of impacts
  - Human
  - Environmental

Heritage Resources

Cultural Site Monitoring Form

Site Number

Site UTMs

Date:
Prepared By:
Agency Affiliation:
Contact Phone/Email:

Resource Name:
Geographic Zone (please circle): Uplands/ Riparian/ Montane
Associated Watershed: Santa Cruz/ Gila/ San Pedro
(Circle One): Prehistoric; **Historic**; **Multicomponent**
Resource Type (describe):
National Register Status (please circle): **Listed**; **Eligible**; **Ineligible**; **Unevaluated**
Management Status (please circle): **Management Plan**; **Interpreted**; **Stabilized**; **Site Stewards**; **Other**

Resource Condition (circle one): **Good (stable)**; **Fair (deteriorating)**; **Poor (Imminently threatened)**; **Destroyed**
New Impacts: Present or Absent (circle one). Type of Impact:________________________

Human (check all that apply)

___Recent footprints
___Litter
___Road/ATV use
___Rock inscription
___Uncovered human remains
___Petroglyph disturbance
___Trails present
___Collector's piles
___Campfires
___Graffiti
___Vehicle tracks
___Construction
___Illegal excavation
___Affected fencing or signs
___Probe holes
___Bullet marks
___Other

Environmental/Animal (check all that apply)

___Burrows/Nests
___Lichen growth
___Spalling/Rock fall
___Livestock
___Erosion
___Vegetation abutment
___Other
___Sediment deposits
___Mineral deposits
___Water runoff
___Trails
___Deterioration

Form provided by Courtney Rose - Pima County

Archaeological Sites Condition

Monitor Date	Description	Eco. Zone(s)	Condition	Impacts	Human Impacts	Environmental Impacts
1/3/18	Empire Ranch	Upland & Riparian	**Fair-to-Good**	In-use interpretive site. Stabilization project on ranch house. Assorted preservation and repair efforts by ERF volunteers, school groups, etc.	Recent footprints, litter, road/ATV use, trails present, vehicle tracks, construction, affected fencing or signs, other (see notes).	Burrows/nests, livestock, vegetation abutment, water runoff, deterioration.
1/3/18	Prehistoric and historic artifact scatter.	Upland	**Good**	Recent tire tracks off main road into site towards east. Brush pile located SE of cattleguard, likely from 2017 Sawmill Fire activity. Existing main road bisects site.	Road/ATV use, vehicle tracks, other (brush pile).	Burrows/nests, livestock, trails.
1/3/18	Prehistoric habitation site and historic ranch.	Upland	**Poor-to-Fair**	Prehistoric artifacts (and subsurface cultural deposits) continue to erode out along road. Historic-age elements continue to deteriorate.	Recent footprints, road/ATV use, construction (see notes).	Burrows/nests, livestock, erosion, vegetation abutment (see notes).
1/3/18	Historic homestead	Upland	**Poor**	Livestock and vehicular use. Location is a livestock water and concentration area.	Road/ATV use, vehicle tracks.	Livestock (abundant), deterioration.

Source: Chris Schrager - BLM

Archaeological Sites Condition

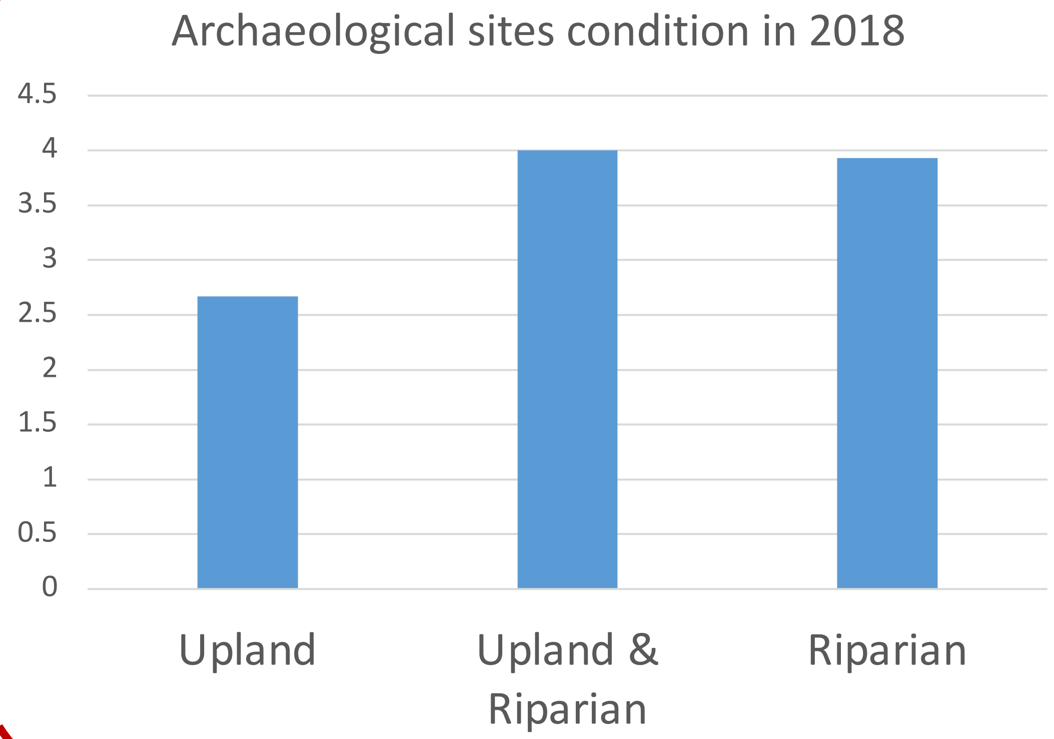

Key:
1= poor
2= poor-to-fair
3= fair
4= fair-to-good
5= good

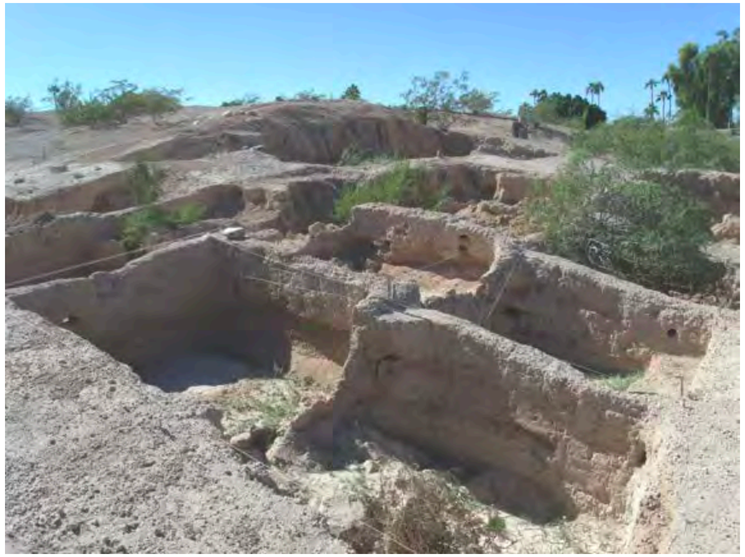

Source: Chris Schrager – BLM and Courtney Rose from Pima County

Archaeological Sites Condition

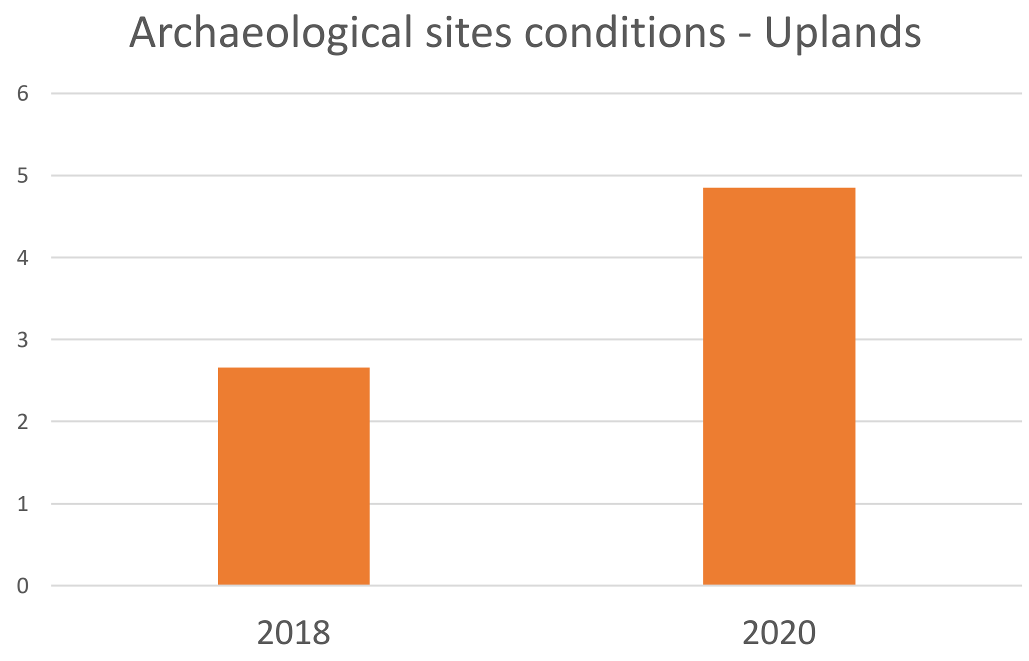

Key:
1= poor
2= poor-to-fair
3= fair
4= fair-to-good
5= good
6 = very good
7 = excellent

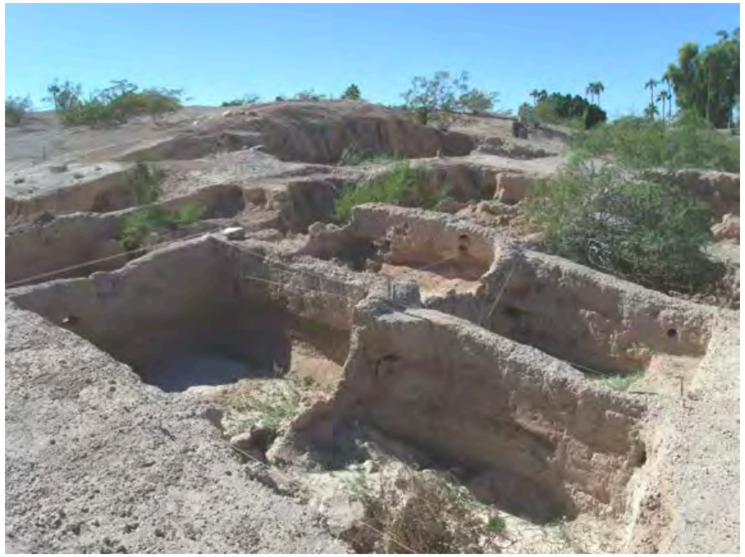

Source: Chris Schrager – Forest Service; Kim Ryan, BLM

Archaeological Sites Condition – Pima County

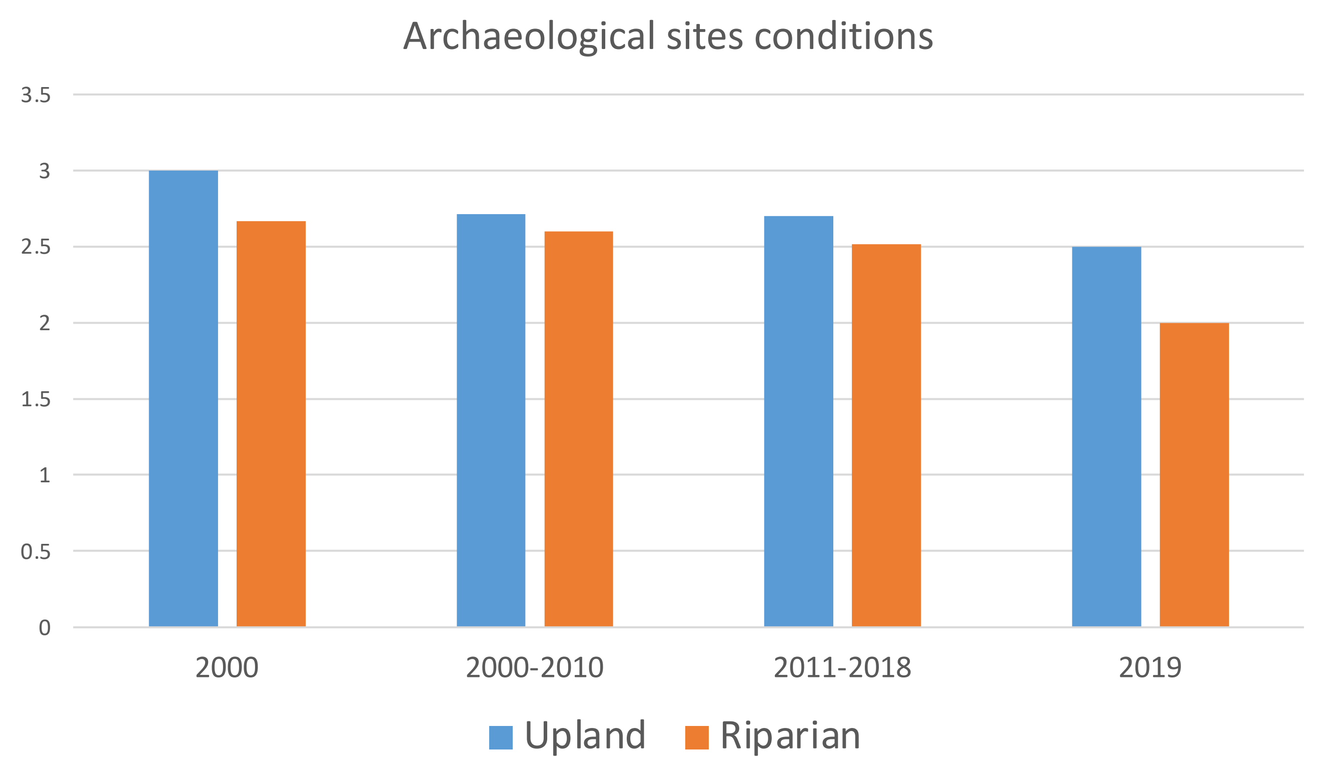

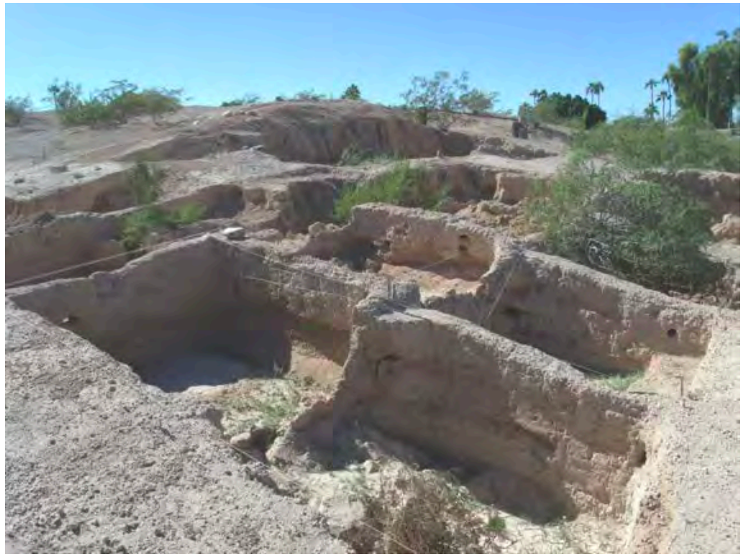

Baseline condition assessment is taken from Stevens (2001). Data between 2001 and 2019 were documented by site stewards.
Next year, we will have data from a survey of 750 acres of land in the CCNP.

Stevens, Michelle N. 2001. *Archaic and Early Agricultural Period Land Use in Cienega Valley, Southeastern Arizona.* PhD Dissertation, Department of Anthropology, University of Arizona, Tucson.

Source: Courtney Rose from Pima County

Archaeological Sites Condition – All sites

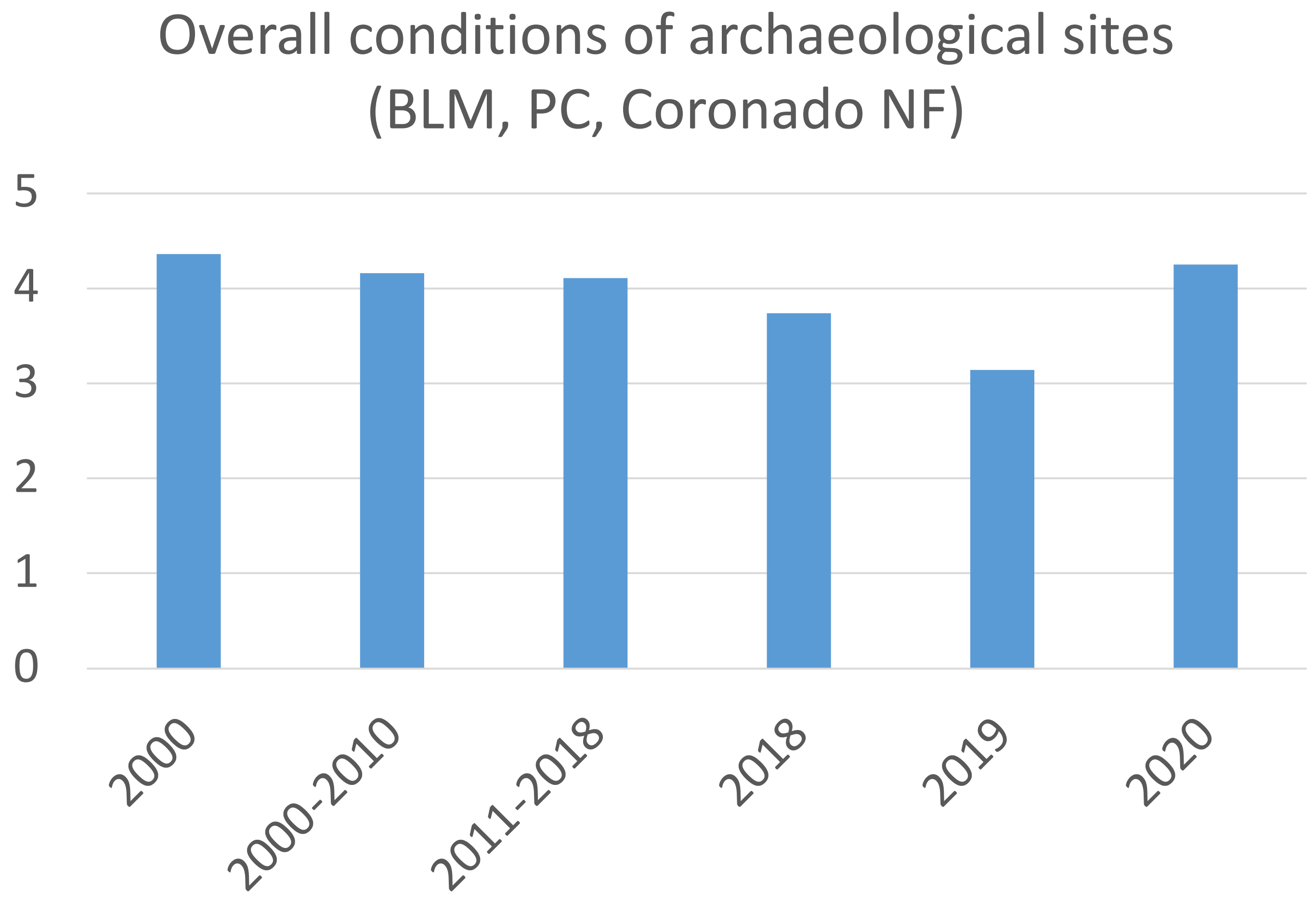

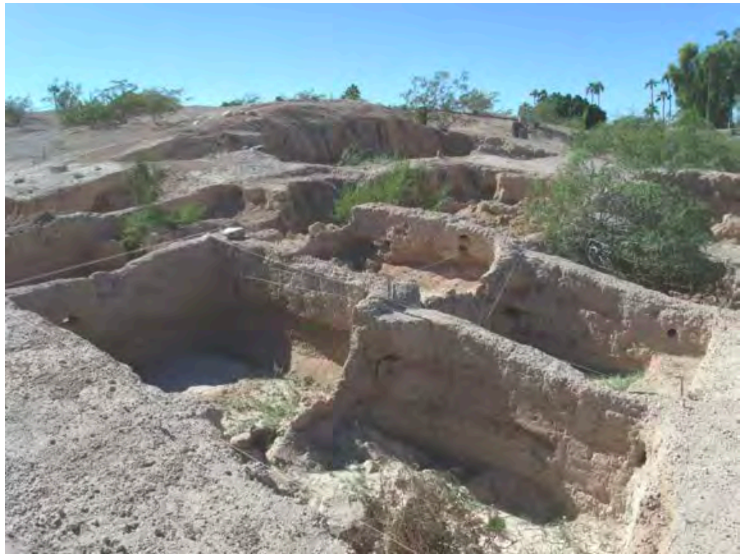

Source: Courtney Rose from Pima County, Chris Schrager from Forest Service, Kim Ryan from BLM

Number of recreational permits - Preserve

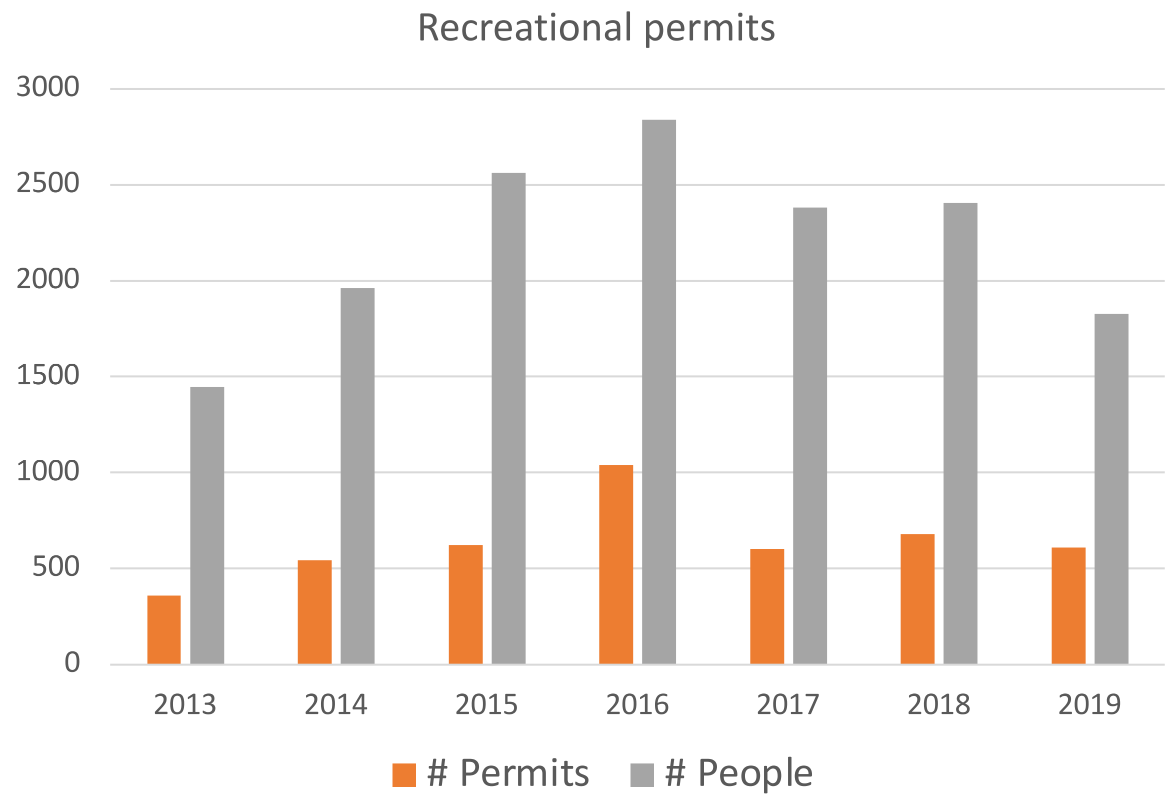

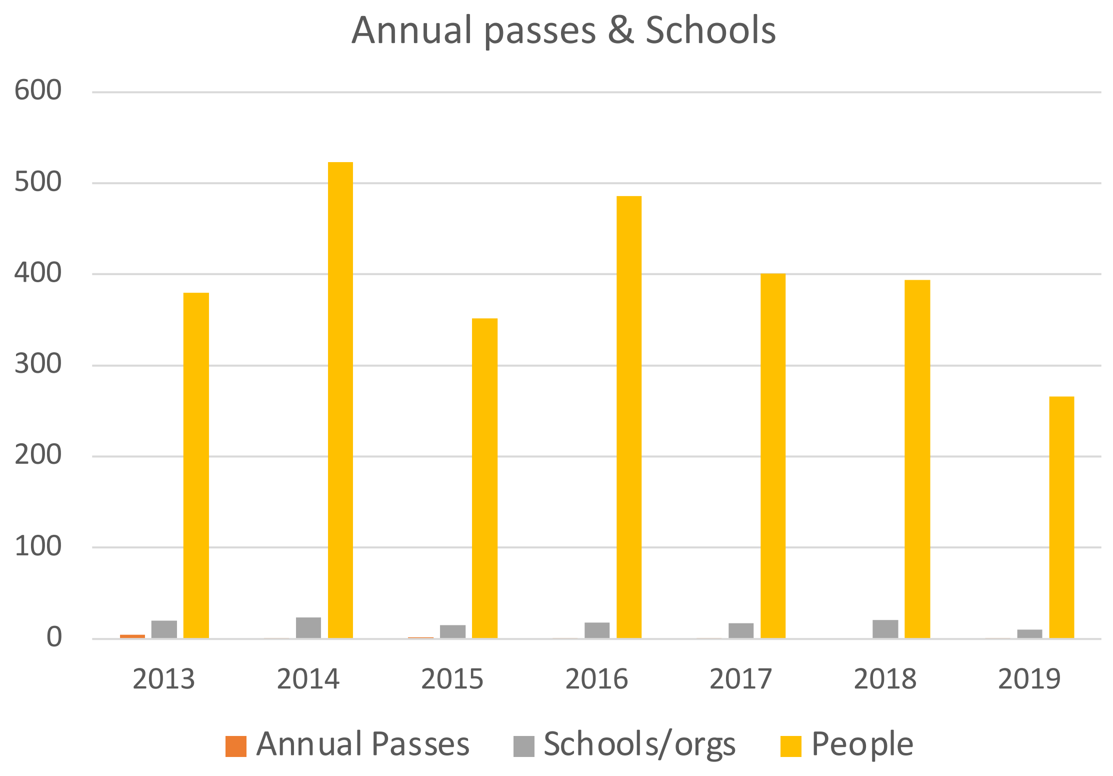

- A limiting factor for schools/orgs has been the lack of restroom facilities in some areas.

Source: MaryAnn Adams and Noemi Zazueta from Pima County Parks and Recreation

Number of recreational permits - LCNCA

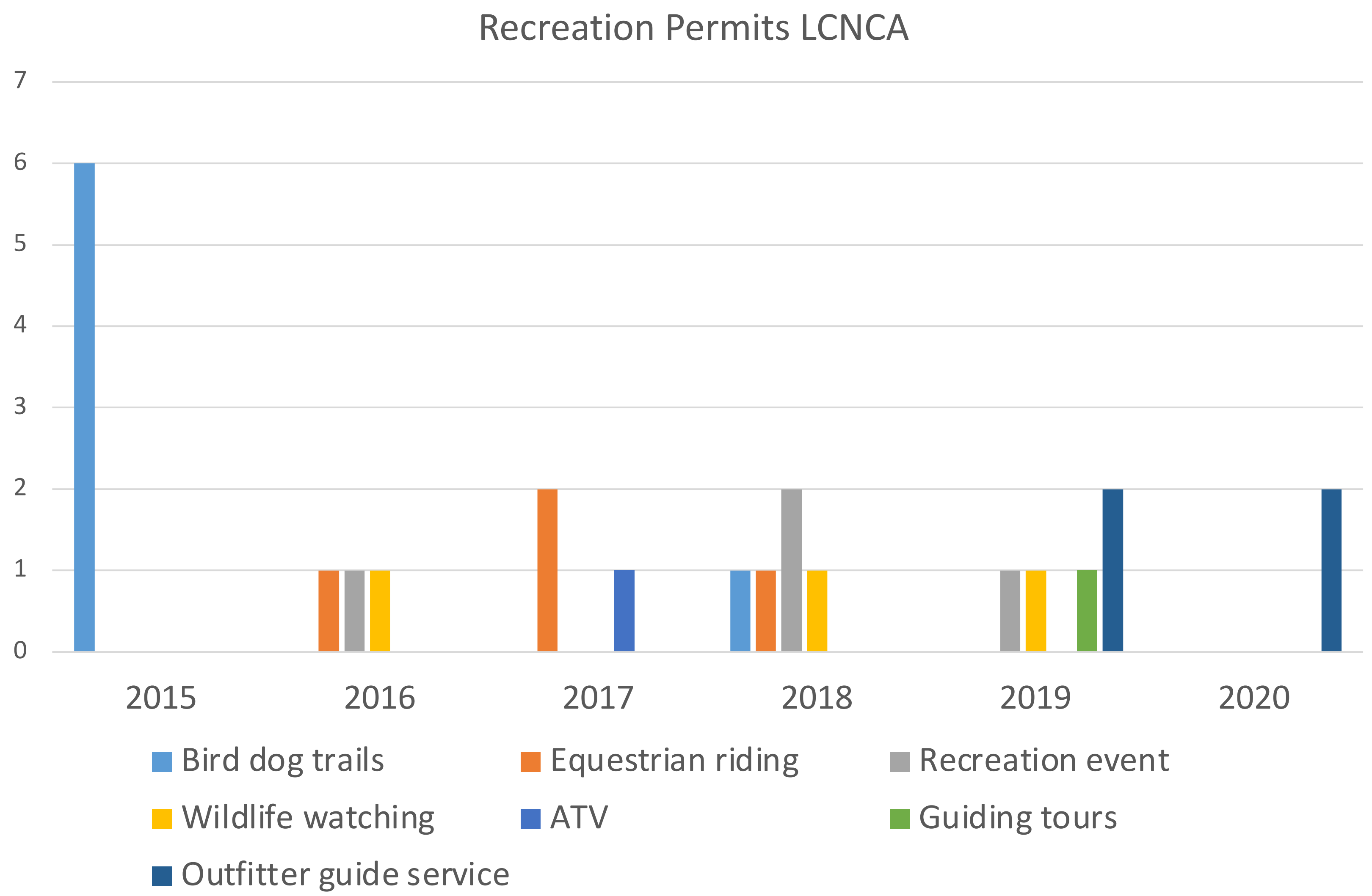

Source: Robert Walter from BLM

Stewardship engagement programs

	Org	Name of program	Started
1	State of Arizona	Arizona Site Stewards	1986
2	Forest Service	Friends of Kentucky Camp Caretakers	1995
3	Various scouting orgs	Scout and Youth Group Work Projects	1995
4	BLM/Forest Service	Camp Hosts	1995
5	Empire Ranch Foundation	Docent and restoration projects	1999
6	Arizona Trail Organization	Trail Volunteers	2000
7	BLM	Pascua Yaqui Youth in Wilderness	2004
8	Vail Preservation Society	Preservation Projects	2006
9	BLM/ERF	Wild about the Grasslands	2007
10	Antelope Foundation	Youth Habitat Improvement	2009
11	Vail School District	Empire High School Ecology Class	2009
12	CWP/BLM/ITE/EHS	Youth Engaged Stewardship (YES!)	2011
13	Sky Island Alliance	Sky Island Alliance Volunteers	2012

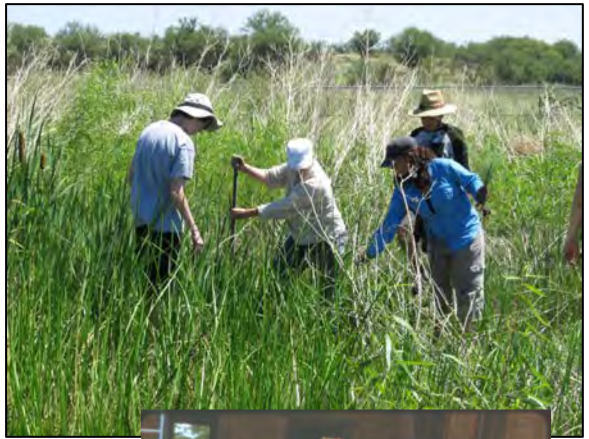

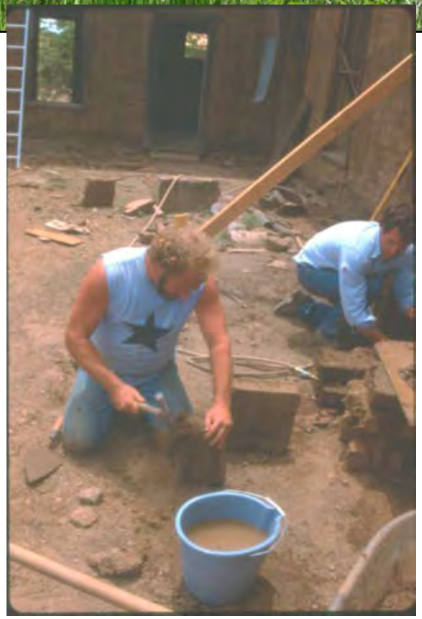

Programs in red are active or intended to be active in 2020 (except for Covid 19)
Programs in blue are known to be currently stopped due to virus restrictions
Programs in green are no longer active

Sources: Shela McFarlin, Dave Mehalic, Chris Schrager,

Socio-cultural - General trends

Economic vitality: Indicators show a decline in economic vitality

Land use – land cover:

- Increase in development with a peak in 1999 and slight decrease in 2009
- Downward trend in shrub cover since 1979

Wells: Dramatic increase in number of wells (cumulative)

Archaeological sites conditions:

- Baseline created for 2018

Recreational permits

- Increase in recreational permits
- Increase in number of visitors
- No clear trend in annual passes and schools/organizations

Stewardship engagement programs:

- Created a baseline for several organizations within the watershed

Conclusion of general trends

Category	No.	Indicator	Description	Ranking
Climate	1	Precipitation	Decrease in winter precipitation in the last 20 years	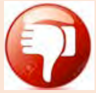
	2	Temperature	Dramatic increase in temperature since 1980	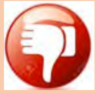
	3	Drought	Since the mid 1990s, we have been in a drought with wet swings	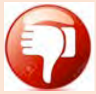
Water	4	Groundwater levels	Although there is a slight recent upward trend, current groundwater levels are still lower than they were prior to the 1980s.	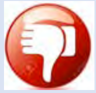
	5	Wetlands	Baseline produced	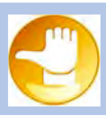
	6	Wet-dry	Slight increase recently, but wet lengths are lower than they were prior to 1999, and significantly lower than the early 1980s	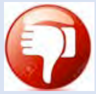
	7	Gauges	Significant decrease in annual average stream flow in Pantano gauge, slight increase in Cienega Creek gauge	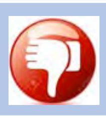
	8	Winter stream flows	Decrease in winter stream flows in CC2 (Preserve) and Upper Cienega Creek (BLM), but slight increase in the Empire Gulch (spring source at BLM)	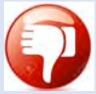
	9	Water quality	Preserve - Decrease in TDS, decrease in PH (except in Davidson 2)<br>BLM – no clear trend	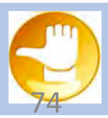

Conclusion of general trends

Category	No.	Indicator	Description	Ranking
Ecological	10	Veg. composition/cover	Shrub cover has been decreasing due to mesquite removal and prescribed-fire projects<br>Perennial grass basal cover has declined, but 2015 and 2016 have shown some recovery<br>Invasive perennial grasses (e.g., Lehman lovegrass) have shown increase<br>Bare ground has decreased, while litter has increased	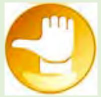
	11	Pronghorn	Slight recovering after a decline in population numbers in 2002	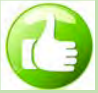
	12	Fish	Endangered species recovering since 2014	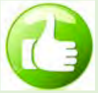
	13	Frogs	Recovery of endangered species and reduction of invasive species since 2013	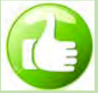
	14	Wildfire	Significant acreage burned in the 1960s. Dramatic increase in acreage burned in the 2000s, resulting in significant damage to vegetation and infrastructure	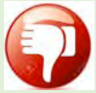

Conclusion of general trends

Category	No.	Indicator	Description	Ranking
Socio-cultural	15	Economic vitality	Baseline created for 2016. Median household income is $47K, median house/condo value is $227K, unemployment is 4.88%, residents below poverty level is 6.22%	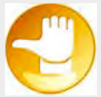
	16	Land use land cover change	Downward trend in shrub cover since 1979. Increase in development with a peak in 1999 and slight decrease in 2009. No clear trend in other land cover classifications	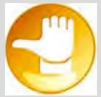
	17	Number of wells	Peak in number of wells installed in the 1990s and decline in more recent decades. Dramatic upward trend since pre 1990s (cumulative)	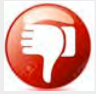
	18	Archaeological site conditions	Intra-agency collaboration to monitor site conditions consistently. First assessment indicate poor to fair conditions.	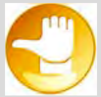
	19	Number of recreational permits	Increase in recreational permits and visitors since 2013, less so in number of schools and orgs	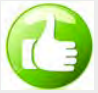
	20	Stewardship engagement programs	13 stewardship engagement programs were identified. The earliest program started operations in the mid 1980s.	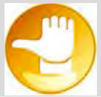

Conclusion of general trends

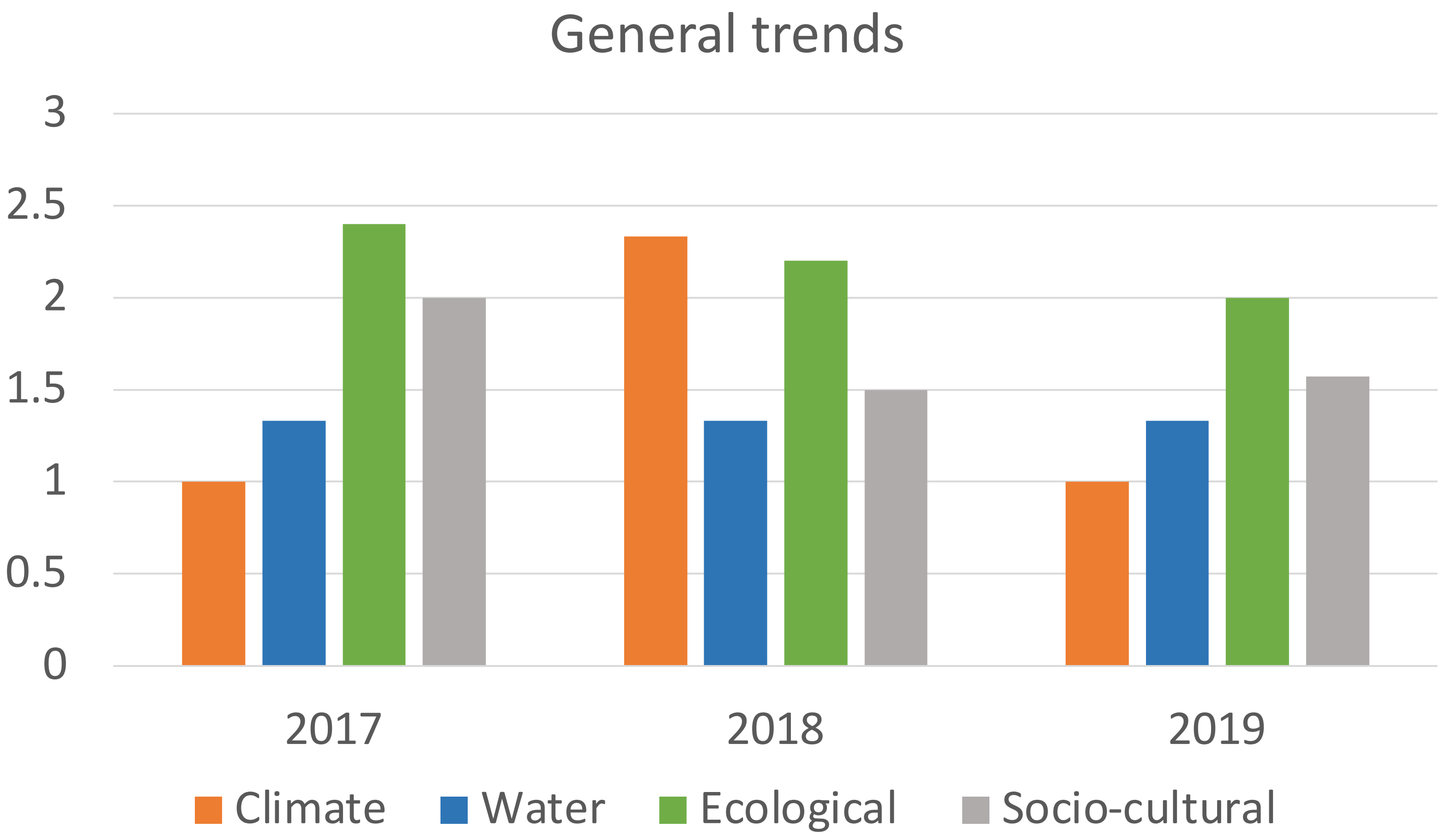

Key:

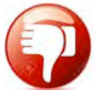
thumbs down = 1

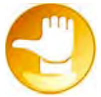
neutral = 2

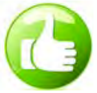
thumbs up = 3

Next steps

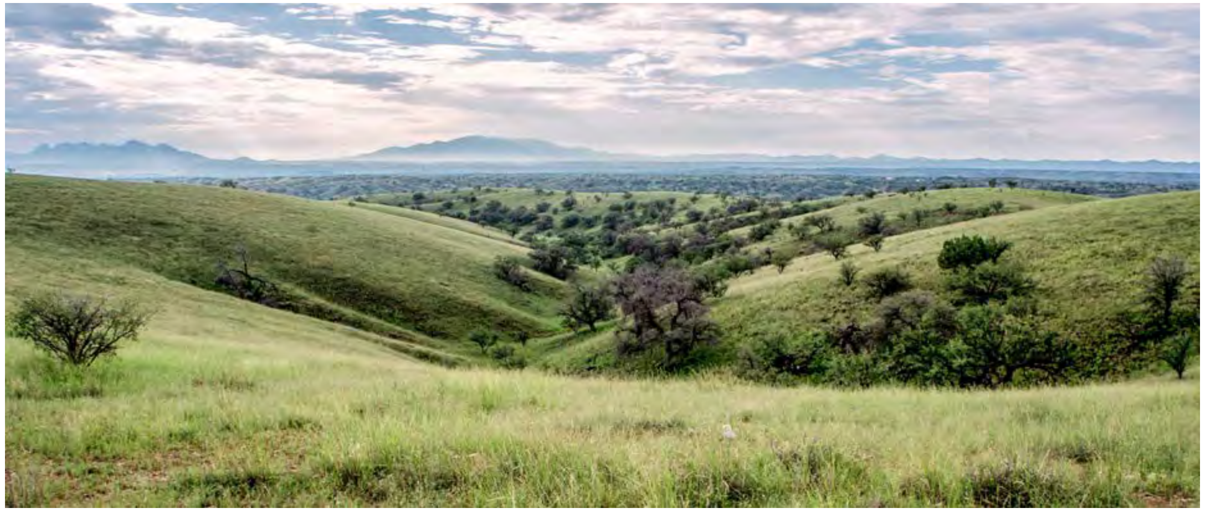

- Critical feedback and suggestions
  - On the indicators and presentation
    - How effective are these indicators?
    - What's missing? What can be eliminated?
    - How can we improve the quality of the presentation?
    - How best to share this with partners and with the public?
  - On responses:
    - What does this information tell us about the State of the Cienega Watershed – trends, concerns, recommendations?

- Following today's session, we will refine, post, and publish

- CWP will continue to work with partners in using these insights to develop appropriate activities and programs

OHMI PIMA COUNTY Arizona

iGLOBES
Interdisciplinary and Global Environmental Studies

CIENEGA WATERSHED
Partnership

Thank you!

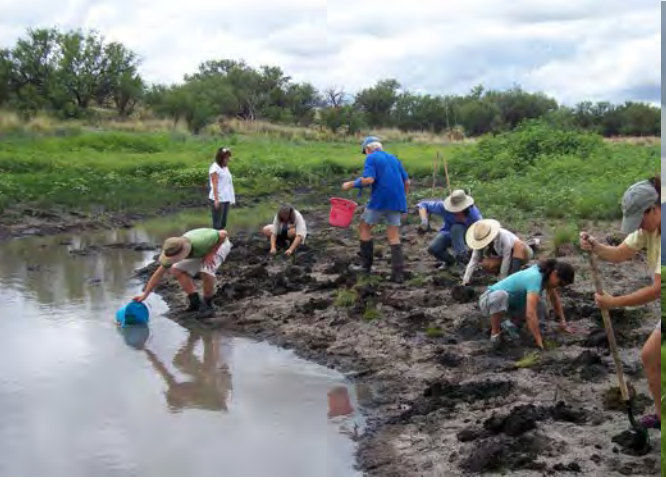
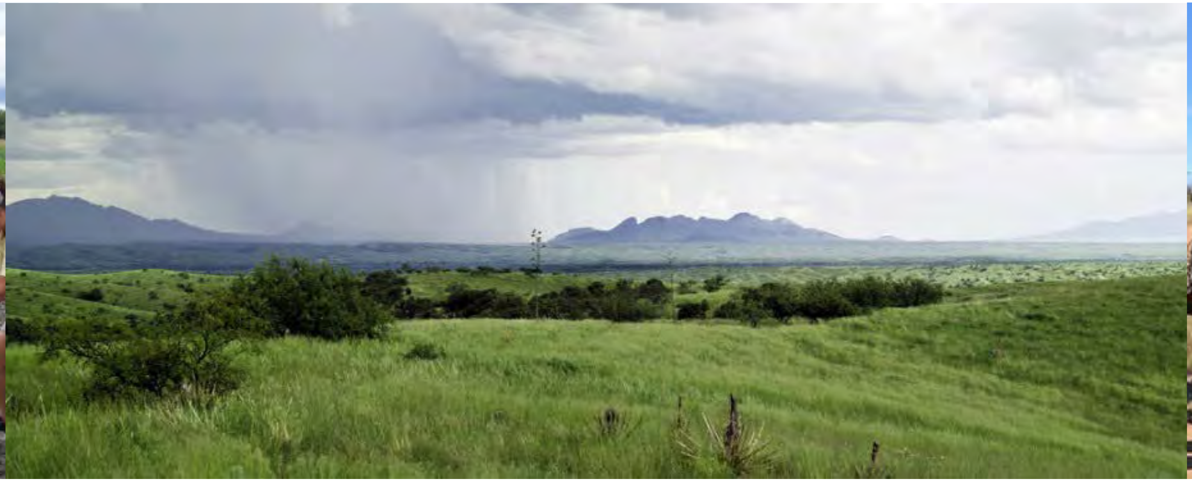
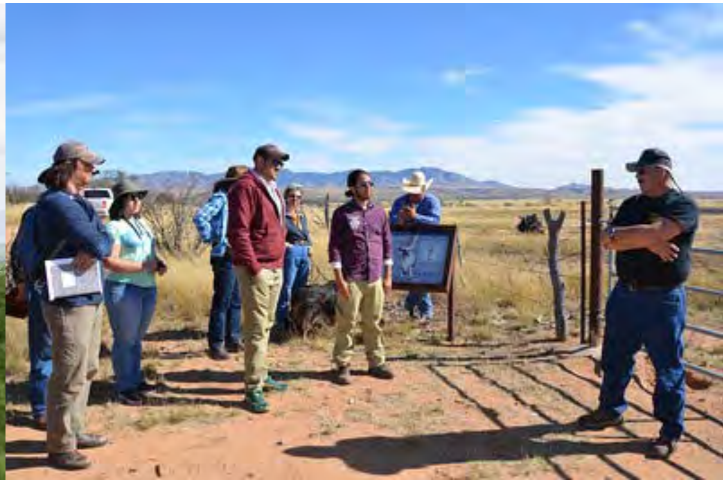

Supplement: Supplementary file 1 — Supplementary file1 (PDF 9962 KB) [file 10661_2021_9741_MOESM1_ESM.pdf]
